# Polygenic background modifies penetrance of monogenic variants for tier 1 genomic conditions

**DOI:** 10.1038/s41467-020-17374-3

**Published:** 2020-08-20

**Authors:** Akl C. Fahed, Minxian Wang, Julian R. Homburger, Aniruddh P. Patel, Alexander G. Bick, Cynthia L. Neben, Carmen Lai, Deanna Brockman, Anthony Philippakis, Patrick T. Ellinor, Christopher A. Cassa, Matthew Lebo, Kenney Ng, Eric S. Lander, Alicia Y. Zhou, Sekar Kathiresan, Amit V. Khera

**Affiliations:** 1 Center for Genomic Medicine, Department of Medicine, Massachusetts General Hospital, Boston, MA USA; 2 Division of Cardiology, Department of Medicine, Massachusetts General Hospital, Boston, MA USA; 3 Department of Medicine, Harvard Medical School, Boston, MA USA; 4 Cardiovascular Disease Initiative, Broad Institute of MIT and Harvard, Cambridge, MA USA; 5 Program in Medical and Population Genetics, Broad Institute of MIT and Harvard, Cambridge, MA USA; 6 Color Genomics, Burlingame, CA USA; 7 Division of Genetics, Brigham and Women’s Hospital, Harvard Medical School, Boston, MA USA; 8 Laboratory for Molecular Medicine, Partners HealthCare Personalized Medicine, Boston, MA USA; 9 Center for Computational Health, IBM Research, Cambridge, MA USA; 10 Department of Biology, MIT, Cambridge, MA USA; 11 Department of Systems Biology, Harvard Medical School, Boston, MA USA; 12 Verve Therapeutics, Cambridge, MA USA

**Keywords:** Genetic association study, Medical genomics, Cardiovascular genetics, Cancer genetics

## Abstract

Genetic variation can predispose to disease both through (i) monogenic risk variants that disrupt a physiologic pathway with large effect on disease and (ii) polygenic risk that involves many variants of small effect in different pathways. Few studies have explored the interplay between monogenic and polygenic risk. Here, we study 80,928 individuals to examine whether polygenic background can modify penetrance of disease in tier 1 genomic conditions — familial hypercholesterolemia, hereditary breast and ovarian cancer, and Lynch syndrome. Among carriers of a monogenic risk variant, we estimate substantial gradients in disease risk based on polygenic background — the probability of disease by age 75 years ranged from 17% to 78% for coronary artery disease, 13% to 76% for breast cancer, and 11% to 80% for colon cancer. We propose that accounting for polygenic background is likely to increase accuracy of risk estimation for individuals who inherit a monogenic risk variant.

## Abstract (web summary)

Genetic variation predisposes to disease via monogenic and polygenic risk variants. Here, the authors assess the interplay between these types of variation on disease penetrance in 80,928 individuals. In carriers of monogenic variants, they show that disease risk is a gradient influenced by polygenic background.

## Introduction

For a range of common heritable diseases, a small subset of the population inherits a rare monogenic variant that causes a large increase in disease risk by disrupting a specific physiological pathway^1^. More recently, polygenic scores have been developed that integrate the effects of many common genetic variants on disease risk^2–5^. While the common variants have small individual effects on disease risk, they can cumulatively have large effects—producing, in some individuals, risks equivalent to the strong monogenic variants^6,7^. A key question is how monogenic and polygenic risk interact: can disease risk from a monogenic variant that causes major disruption to a specific pathway be meaningfully modified by polygenic risk factors that involve small perturbations to a wide range of cellular pathways?

Recent work has suggested that common variant background modifies the age of disease onset among carriers of high-risk trinucleotide repeats predisposing to Huntington’s disease, the p.Gln368Ter *MYOC* variant predisposing to glaucoma, and continuous measures, such as height, body mass index, and cholesterol levels among those with rare monogenic mutations^6,8–10^. With respect to common diseases such as cancer, the Consortium of Investigators of Modifiers of BRCA1/2 (CIMBA) studied a large number of *BRCA1/2* monogenic risk variant carriers recruited from cancer genetics clinics, noting a relationship between a polygenic score comprised of genome-wide significant loci and the risk of breast, ovarian, and prostate cancer^11,12^.

Here, we set out to confirm and extend these prior observations for the three tier 1 genomic conditions highlighted by the U.S. Centers for Disease Control and Prevention—familial hypercholesterolemia, hereditary breast and ovarian cancer, and Lynch syndrome^13^. About 1% of asymptomatic adults carry a pathogenic or likely pathogenic variant related to any of these conditions^14,15^. Although such variants are associated with several-fold increased risk of disease, it has long been recognized that they have incomplete penetrance and variable expressivity. For example, in one U.S. healthcare system, more than 50% of familial hypercholesterolemia variant carriers and more than 70% of female hereditary breast and ovarian cancer variant carriers remained free of disease well into middle age^16,17^. We hypothesized that this incomplete penetrance could be partly explained by polygenic background—a topic that has both scientific implications about disease physiology and clinical implications for genetic counseling.

We designed two case-control studies from the UK Biobank and Color Genomics commercial testing laboratory and performed analysis of an independent cohort from the UK Biobank. The two case-control studies increase statistical power by enriching for disease cases, whereas the independent UK Biobank cohort enables assessment of outcomes for both carrier and noncarriers of monogenic risk variants within the context of contemporary medical care^18–20^. Moreover, we compute newer generation polygenic scores for coronary artery disease, breast cancer, and colorectal cancer, which enhance risk prediction over prior scores^7,21,22^.

## Results

### Coronary artery disease

We first studied the interplay of monogenic risk variants and polygenic scores in coronary artery disease. To identify individuals with monogenic variants causal for familial hypercholesterolemia, we sequenced the three genes related to the condition—*LDLR, APOB*, and *PCSK9*—in 6432 coronary artery disease cases and 6420 controls derived from the UK Biobank (Table 1)^18^. Each of the observed genetic variants was reviewed by a laboratory geneticist blinded to any phenotype data and classified according to current clinical guidelines (Supplementary Table 1)^23^. A total of 28 distinct genetic variants were classified as pathogenic or likely pathogenic; they were present in 43 (0.67%) cases and 13 (0.20%) controls. The presence of a familial hypercholesterolemia variant conferred a 3.21-fold increased risk of coronary artery disease (95% confidence interval [CI] 1.72–5.99) when assessed in a logistic regression model adjusted for age, sex, and the first four principal components of ancestry.

**Table 1:** Baseline characteristics of coronary artery disease case-control study participants.

	Cases with coronary artery disease (*n* = 6432)	Controls (*n* = 6420)
Age, mean (SD), yr	68.3 (7.2)	68.3 (7.2)
Female sex, *n* (%)	2248 (34.9)	2234 (34.8)
Race, *n* (%)		
White	5963 (92.7)	6188 (96.4)
Black	72 (1.1)	58 (0.9)
Asian	254 (3.9)	90 (1.4)
Other	143 (2.2)	84 (1.3)
Hypertension, *n* (%)	4565 (71.0)	2011 (31.3)
Diabetes, *n* (%)	1553 (24.1)	356 (5.5)
Chronic kidney disease, *n* (%)	413 (6.4)	47 (0.7)
Current or former smoking, *n* (%)	4256 (66.6)	2862 (44.7)
Body mass index, mean (SD), kg m^−2^	29.60 (5.4)	27.26 (4.4)
Family history of heart disease, *n* (%)	1986 (39.3)	1345 (26.6)

We next examined the effect of participants’ polygenic background on risk of coronary artery disease, by computing a previously validated polygenic score in all cases and controls^7^.

Even among carriers of a familial hypercholesterolemia variant, the observed risk varied substantially according to the polygenic score (Supplementary Tables 2 and 3). Odds ratio per standard deviation increment in the polygenic score were 2.31 (1.16–4.57) and 1.74 (1.68–1.81) for carriers and noncarriers, respectively. Within the limitations of statistical power, we did not observe a significant interaction between polygenic score and familial hypercholesterolemia variant status (*p*-interaction 0.60, Wald Test and Methods).

**Fig. 1:**
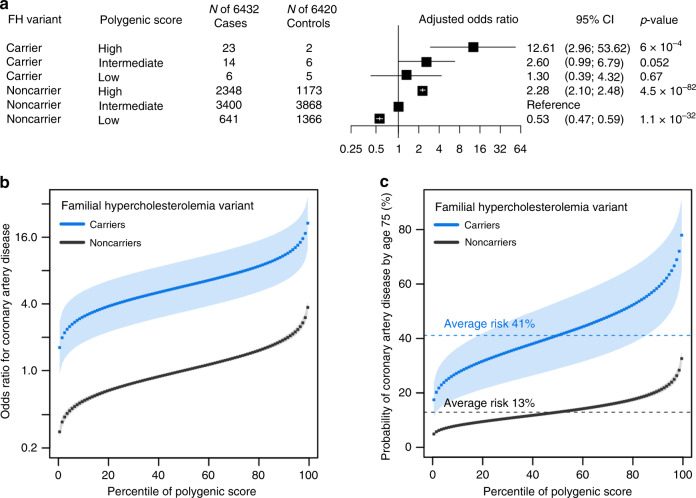
Interplay of monogenic and polygenic risk for coronary artery disease. **a** Risk of coronary artery disease by monogenic and polygenic risk strata (case-control study; *n* = 12,852). Carriers and noncarriers were stratified into three groups according to their polygenic score—low, intermediate, or high defined as the lowest quintile, the middle three quintiles, and the highest quintile of the polygenic score distribution, respectively. The odds ratio was assessed in a logistic regression model with age, sex, and the first four principal components of ancestry as covariates. Noncarriers with intermediate polygenic score served as the reference group. The black boxes indicate the adjusted odds ratio. The horizontal lines around the black boxes indicate the 95% confidence intervals. **b** Predicted odds ratio for coronary artery disease in each percentile (dots) of the polygenic score distribution for carriers (blue) and noncarriers (black) of familial hypercholesterolemia variants in the cohort study derived from the UK Biobank (*n* = 48,812). Noncarriers with median polygenic score served as the reference group. **c** Predicted probability of coronary artery disease by age 75 years in each percentile (dots) of the polygenic score distribution for carriers (blue) and noncarriers (black) of familial hypercholesterolemia variants in the cohort study derived from the UK Biobank (*n* = 48,812). The shaded area around the dots represents the 95% confidence interval. The horizontal dashed lines show the probability of disease for people with average polygenic risk score. FH familial hypercholesterolemia. *p*-values in the figure were estimated by the Wald Test. Statistical significance was set at *p* < .05, and two-sided *p* values were used.

We classified individuals as having low polygenic score (lowest quintile), intermediate polygenic score (middle three quintiles), or high polygenic score (highest quintile). Compared to non-carriers with intermediate polygenic score, the risk among mutation carriers ranged from 1.30-fold (95% CI 0.39–4.32) for those in the lowest quintile of the polygenic score distribution to 12.61 (95% CI 2.96–53.62) in the highest quintile (Fig. 1a). To test the hypothesis that the risk gradient among monogenic variant carriers according to the polygenic score operated via pathways largely unrelated to the monogenic variants, we conducted two additional sensitivity analyses. First, we removed all variants from the polygenic score located within 1 megabase of the three familial hypercholesterolemia genes. Second, we sought to remove the impact of the polygenic score on LDL cholesterol by using the residuals from a linear regression model that additionally included a previously derived polygenic score for LDL cholesterol. In each case, effect estimates were minimally changed (Supplementary Table 4 and Methods). These results suggest that the risk for coronary artery disease captured by the polygenic score—among both carriers and noncarriers of a monogenic variant—is largely independent of LDL cholesterol pathways.

We next examined an independent cohort of 48,812 unrelated UK Biobank participants. A laboratory geneticist identified a familial hypercholesterolemia variant in 130 (0.27%) of these participants, 18 (0.87%) cases, and 112 (0.24%) controls, which conferred a 5.08-fold (95% CI 3.01–8.57) increased risk of coronary artery disease at the time of enrollment (Supplementary Tables 5–7). The polygenic score for coronary artery disease was normally distributed in the population and strongly associated with disease—odds ratio per standard deviation increment of 1.64 (95% CI 1.57–1.72). Within a logistic regression model, the relationship between the polygenic score and prevalent disease conformed to a linear model (Supplementary Fig. 1 and Supplementary Table 8).

Joint modeling of monogenic variant carrier status and polygenic score indicated substantial gradients in risk of coronary artery disease according to inherited DNA variation that can be assessed from the time of birth. Odds ratio for coronary artery disease in monogenic variant carriers—as compared to noncarriers with median polygenic score—ranged from 1.62 to 21.44 across percentiles of the polygenic score (Fig. 1b). Modeling the probability of disease by age 75 years using a Cox regression model suggested striking gradients in risk, ranging from 4.9% for noncarriers in the lowest percentile of the polygenic score to 77.9% for monogenic risk variant carriers in the highest polygenic score percentile (Fig. 1c).

### Breast cancer

We next set out to apply the same analysis to breast cancer. We first identified monogenic risk variants by sequencing the *BRCA1* and *BRCA2* genes in 1920 breast cancer cases and 17,344 controls, all female, from the Color Genomics commercial testing laboratory (Table 2)^20^. A pathogenic or likely pathogenic variant was identified in 174 (9.1%) cases and 671 (3.9%) controls, corresponding to a 3.48-fold (95% CI 2.81–4.21) increased risk of breast cancer in variant carriers. We then calculated polygenic risk for breast cancer, using a previously validated polygenic score^21^. As we saw for coronary artery disease, breast cancer risk was strongly affected by polygenic background even for those who carried a pathogenic variant (Supplementary Tables 2 and 3). Within the limits of statistical power, the impact of the polygenic score appeared similar in carriers and noncarriers, odds ratio per standard deviation increment of 1.44 (1.19–1.74) and 1.57 (1.49–1.65), respectively, *p*-interaction = 0.94 (Wald Test and Methods).

**Table 2:** Baseline characteristics of breast cancer case-control study participants.

	Cases with breast cancer (*n* = 1920)	Controls (*n* = 17,344)
Age, mean (SD), yr	57.4 (12.5)	45.9 (13.5)
Female sex, *n* (%)	1920 (100)	17,344 (100)
Race, *n* (%)		
White	1375 (71.6)	12,365 (71.3)
Black	30 (1.5)	410 (2.4)
Asian	83 (4.3)	695 (4.0)
Other	432 (22.6)	3874 (22.3)
Body mass index, mean (SD), kg m^−2^	26.5 (6.7)	27.2 (6.8)
Family history of breast cancer, *n* (%)	855 (44.5)	7497 (43.2)

**Fig. 2:**
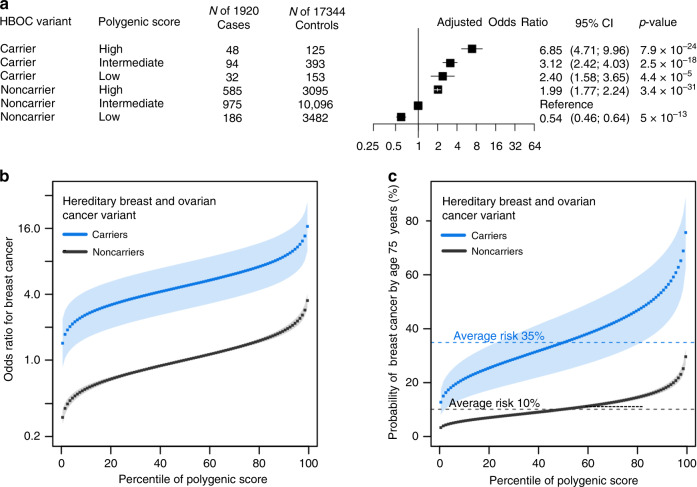
Interplay of monogenic and polygenic risk for breast cancer. **a** Risk of breast cancer by monogenic and polygenic strata (case-control study; *n* = 19,264). Carriers and noncarriers were stratified into three groups according to their polygenic score—low, intermediate, or high defined as the lowest quintile, the middle three quintiles, and the highest quintile of the polygenic score distribution, respectively. The odds ratio was assessed in a logistic regression model with age and the first four principal components of ancestry as covariates. Noncarriers with intermediate polygenic score served as the reference group. The black boxes indicate the adjusted odds ratio. The horizontal lines around the black boxes indicate the 95% confidence intervals. **b** Predicted odds ratio for breast cancer in each percentile (dots) of the polygenic score distribution for carriers (blue) and noncarriers (black) of hereditary breast and ovarian cancer variants in the cohort study derived from the UK Biobank (*n* = 26,597). Noncarriers with median polygenic score served as the reference group. **c** Predicted probability of coronary artery disease by age 75 years in each percentile (dots) of the polygenic score distribution for carriers (blue) and noncarriers (black) of hereditary breast and ovarian cancer variants in the cohort study derived from the UK Biobank (*n* = 26,597). The shaded area around the dots represents the 95% confidence interval. The horizontal dashed lines show the probability of disease for people with average polygenic risk score. HBOC hereditary breast and ovarian cancer. *p*-values in the figure were estimated by the Wald Test. Statistical significance was set at *p* < .05, and two-sided *p* values were used.

Compared to noncarriers with intermediate polygenic score, increased risk among carriers ranged from 2.40-fold (95% CI 1.58–3.65) for those in the lowest quintile of the polygenic score distribution to 6.85-fold (95% CI 4.71–9.96) in the highest quintile (Fig. 2a).

We again extended these results into the UK Biobank participants, this time focusing only on the 26,597 female participants (Supplementary Table 9). A laboratory geneticist reviewed all observed genetic variants in the *BRCA1* and *BRCA2* genes (Supplementary Table 5), identifying 115 carriers of pathogenic or likely pathogenic variants. These variants conferred a 4.47-fold increased risk of breast cancer (95% CI 2.76–7.24). The polygenic score was associated with an odds ratio per standard deviation increment of 1.61 (95% CI 1.52–1.70), with a linear relationship to the risk of breast cancer (Supplementary Fig. 1 and Supplementary Table 8).

Joint modeling of both monogenic variant status and polygenic score estimated that the risk for breast cancer among carriers of a *BRCA1* or *BRCA2* variant ranges from 1.43-fold to 16.68-fold increased risk across percentiles of the polygenic score (Fig. 2b). When modeled as probability of disease by age 75 years, risk among monogenic variant carriers ranged from 12.7% to 75.7% and risk among noncarriers ranged from 3.3% to 29.6% (Fig. 2c).

### Colorectal cancer

**Fig. 3:**
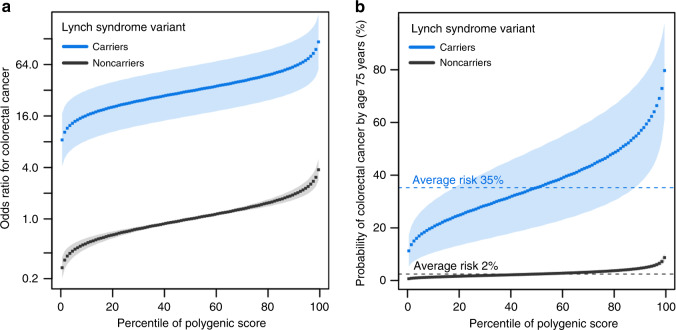
Interplay of monogenic and polygenic risk for colorectal cancer. **a** Predicted odds ratio for colorectal cancer in each percentile (dots) of the polygenic score distribution for carriers (blue) and noncarriers (black) of Lynch syndrome variants in the cohort study derived from the UK Biobank (*n* = 48,812). Noncarriers with median polygenic score served as the reference group. **b** Predicted probability of colorectal cancer by age 75 years in each percentile (dots) of the polygenic score distribution for carriers (blue) and noncarriers (black) of Lynch syndrome variants in the cohort study derived from the UK Biobank (*n* = 48,812). The shaded area around the dots represents the 95% confidence interval. The horizontal dashed lines show the probability of disease for people with average polygenic risk score.

We studied a third disease, colorectal cancer, in the same set of 48,812 UK Biobank participants used above (Supplementary Tables 6 and 10). A pathogenic or likely pathogenic Lynch syndrome variant in any of four genes (*MLH1*, *MSH2*, *MSH6*, and *PMS2*) was identified in 76 (0.15%) individuals (Supplementary Table 5), conferring an odds ratio for colorectal cancer of 27.86 (95% CI 14.35–54.10). The odds ratio per standard deviation increment in the colorectal cancer polygenic score was 1.65 (95% CI 1.48–1.85). Joint modeling of monogenic variants and the polygenic score—using noncarriers with median polygenic score as the reference group—noted odds ratios ranging from 8.41 to 117.80 for carriers of monogenic variants and 0.27 to 3.76 for noncarriers across polygenic score percentiles (Fig. 3a). Estimated absolute risk of colorectal cancer by age 75 years ranged from 11.3% to 79.7% for carriers and 0.7% to 8.7% for noncarriers (Fig. 3b).

## Discussion

Our analysis of the interplay between monogenic risk variants and polygenic background—for the three CDC tier 1 genomic conditions: familial hypercholesterolemia, hereditary breast and ovarian cancer syndrome, and Lynch syndrome—has at least two implications.

First, we showed that risk conferred by monogenic risk variants, which act by perturbing a specific molecular pathway, can be substantially modified by polygenic background, which appears to act by affecting a diverse set of physiological processes. Taking familial hypercholesterolemia as an example, monogenic variants predispose to premature coronary artery disease through dysregulation of clearance of LDL cholesterol from the circulation. By contrast, only a small minority (~20%) of common DNA variants that predispose to coronary artery disease operate via cholesterol-related pathways, with the remainder affecting non-cholesterol-related pathways (such as inflammation, cellular proliferation, vascular tone) and many additional pathways yet to be discovered^24^. Similarly, pathway analyses indicate that <20% of common variants linked to breast or colorectal cancer affect genes involved in the DNA repair pathways perturbed by the monogenic hereditary breast and ovarian cancer or Lynch syndrome variants^22,25^. From a physiological standpoint, additional study is needed to understand how the major disruptions caused by monogenic variants can be offset by other factors. Yet, the risk for monogenic variant carriers with the lowest polygenic risk scores approached the population average. These results are largely consistent with a liability threshold model, whereby the probability that any given pathogenic variant carrier crosses the threshold into disease is influenced by the underlying liability conferred by the polygenic background^26^. Understanding the physiological basis of how polygenic score modifies the penetrance of monogenic variants may suggest therapeutic strategies for monogenic variant carriers in general.

Second, our findings indicate that accounting for polygenic background is likely to increase the accuracy of risk estimation for individuals who inherit a monogenic risk variant. An important example is the decision about the timing and intensity of lipid-lowering therapy for individuals with familial hypercholesterolemia. Here, we find a broad spectrum of risk of coronary artery disease in those with a monogenic risk variant across percentiles of the polygenic score that may better inform shared decision making—odds ratios compared to noncarriers ranging from 1.62 to 21.44 and an absolute risk at age 75 years ranging from 17.5% to 77.9%. Refined risk estimates may also help with counseling and clinical decision making for hereditary cancer. In breast cancer, carriers of pathogenic *BRCA1* or *BRCA2* variants are faced with the decision about whether to undergo prophylactic mastectomy as opposed to serial imaging^27^. At present, up to 50% of women opt for prophylactic mastectomy, but rates are highly variable^28,29^. For Lynch syndrome, the current guidelines recommend initiating screening colonoscopy at age 20–25 years and repeating every 1–2 years^30^. Aspirin has been shown to reduce the risk of colon cancer in Lynch syndrome, but the optimal dose and duration is unknown^31^. Many experts advocate for prophylactic colectomy in a subset of patients, but this remains controversial^32^. More accurate risk estimation could better inform the onset and frequency of serial colonoscopies, the risk–benefit ratio for aspirin, or the decision for prophylactic colectomy.

In a clinical setting, assessing both rare monogenic risk variants and polygenic risk requires high-coverage sequencing of genes associated with monogenic risk and an approach to assay common variants across the genome. At present, this can be accomplished by various approaches, including (i) high-coverage whole genome sequencing^33^, (ii) whole exome sequencing and genotyping array, or (iii) targeted high-coverage sequencing of individual genes and low-coverage sequencing across the genome^34^. Ongoing efforts to improve the cost and accessibility of these technologies will improve the feasibility of incorporating this information into routine clinical practice.

Our results should be interpreted in the context of potential limitations. First, UK Biobank participants tend to be healthier than the general population, and Color Genomics participants are likely to be enriched for genetic disease compared to the general population^35^. Within any target population, a comparison of predicted and observed disease rates can allow for recalibration of risk estimates, as recently performed for a breast cancer prediction tool^36^. Second, our analysis focused on the role of monogenic variants in the nine genes associated with the three CDC tier 1 genomic conditions; additional efforts are needed to include variants in other known or newly discovered genes in the future. Representative examples of such genes include those encoding lipoprotein lipase (*LPL*), where variants increase risk of coronary artery disease via perturbation of triglyceride metabolism, and checkpoint kinase 2 (*CHEK2*), which increases risk of breast cancer via perturbation of DNA repair pathways^37,38^. Third, we aggregated together all pathogenic and likely pathogenic variants for each monogenic condition across the known causal genes. Although this approach is consistent with current clinical treatment guidelines—which recommend similar treatment strategies for each condition regardless of which gene is implicated—risk is known to vary somewhat according to specific gene or variant (Supplementary Figs. 2–4)^39–41^. Fourth, further efforts are needed to understand how best to disclose integrated genomic risk assessments to patients and treating clinicians and how to integrate them with existing lifestyle or clinical risk factors^42–44^.

Finally, we continue to highlight an important equity issue. Our knowledge concerning the monogenic risk variants and the development of the polygenic scores has been based primarily on patients of European ancestry, which affects the utility for patients of other ancestries^33,45,46^. In particular, the polygenic scores are known to be less precise for other ancestry groups^46^. It is important for the biomedical community to invest in the development of more diverse population allele frequency databases^47,48^, disease association studies in other ancestral backgrounds, new computational algorithms that better account for ancestral background^49^, and new technology or machine-learning algorithms to enable unbiased high-throughput functional assessments of variants^50,51^.

## Methods

### Study populations

The study populations consisted of two case-control studies and one national biobank cohort. First, a coronary artery disease case-control study of 12,852 participants (6432 cases and 6420 controls) was derived from the UK Biobank, a prospective national biobank study that enrolled middle-aged adult participants between 2006 and 2010^18^. Coronary artery disease cases were defined centrally based on self-report at enrollment, hospitalization records, or death registry records (http://Biobank.ndph.ox.ac.uk/showcase/showcase/docs/alg_outcome_mi.pdf). Controls included participants free of any self-reported or documented history of coronary artery disease. Only independent samples were used by removing one from a pair of related individuals whenever genetic relationship was closer than second-degree.

Second, a breast cancer case-control study consisted of 19,264 women (1920 cases and 17,344 controls) who underwent clinical grade genetic testing for hereditary breast and ovarian cancer syndrome at a commercial testing laboratory (Color Genomics; Burlingame, CA). Breast cancer case ascertainment was based on self-report at the time of enrollment^20^. Since participants derived from commercial genetic testing companies tend to be enriched for relatedness, participants that were related (up to second degree) and participants ascertained through Color Genomics’ cascade screening program were not included in this study.

Third, a cohort derived from the UK Biobank consisting of 48,812 participants that underwent exome sequencing at Regeneron Genetics Center was used^19^. There was no overlap between this cohort and the 12,852 participants of the case-control study. Extensive clinical data including diagnosis of coronary artery disease and cancer are available on all participants. Coronary artery disease was defined based on self-report of heart attack/myocardial infarction, hospitalization records confirming a diagnosis of acute myocardial infarction or ischemic heart disease, coronary revascularization procedures (coronary artery bypass graft surgery or percutaneous angioplasty/stent placement), or death registry data indicating ischemic heart disease or myocardial infarction as a cause of death. Breast and colorectal cancer were each defined based on self-report of the diagnosis, hospitalization records, cancer registry data specifying type of cancer, and death registry. For each of the three diseases, we considered the earliest date at which the diagnosis was ascertained as the diagnosis date. All participants diagnosed at dates prior to enrollment in the UK Biobank were considered prevalent at baseline, while participants diagnosed after enrollment were considered incident (Supplementary Table 6). Additional details on ascertainment of each of the three disease states are provided in Supplementary Table 11. Only independent samples were used by removing one from a pair of related individuals whenever genetic relationship was closer than second-degree.

Informed consent was obtained from all participants. Analysis of UK Biobank data was performed under application number 7089 and approved by the Partners Healthcare institutional review board. The commercial testing laboratory cohort was approved by the Western Institutional Review Board (protocol number 20150716).

### Gene sequencing

Whole exome sequencing on 12,909 samples from the coronary artery disease case-control study derived from the UK Biobank was performed at the Broad Institute of MIT and Harvard (Cambridge, MA) as described previously^52^. Libraries were constructed and sequenced on an Illumina HiSeq sequencing using 151 bp pair-end reads^53^. An Illumina Nextera Exome Kit was used for in-solution hybrid selection. Sequencing reads were aligned to the human reference genome build GRCh37.p13 using the Burrows–Wheeler Aligner algorithm^54^, and aligned non-duplicate reads were locally realigned and base quantiles were recalibrated using the Genome Analysis Toolkit software^55,56^. Variants were jointly called using the HaplotypeCaller module of the Genome Analysis Toolkit. We removed samples with contamination >10% (*n* = 0), samples with <80% of target bases at 20× coverage (*n* = 0), putative sex chromosome aneuploidy (*n* = 17), outliers for heterozygozity (*n* = 4), genotype call rate <95% (*n* = 6), and samples for which there is no genotyping array data (*n* = 3). There were also 27 individuals excluded due to sample relatedness. Variants from the remaining 12,852 unrelated samples were carried forward for further analysis. The mean target coverage was 75×, and 91.1% of target bases were captured at >20× sequencing depth.

For the 19,264 samples from the breast cancer case-control cohort, target-enrichment sequencing was performed at the laboratory of Color Genomics (Burlingame, CA) as previously described^20^. The Color Genomics laboratory is in compliance with Clinical Laboratory Improvement Amendments (number 05D2081492) and College of American Pathologists (number 8975161). In brief, sequencing reads were aligned to the human reference genome build GRCh37.p12 using the Burrows–Wheeler Aligner algorithm, and duplicated and low-quality reads were discarded. Variants were then jointly called using the HaplotypeCaller module of GATK3.4 and SAMtools version 1.8^20^. A no template control and two positive controls containing a set of known variants were included in every batch of samples. Strict coverage requirements (20 unique reads for each base) were used, and median coverage ranged between 200× and 300×.

Finally, whole exome sequencing of 49,960 UK Biobank participants was performed at the Regeneron Genetics Center using 75 base pair paired-end reads with two 10 base pair index reads on the Illumina NovaSeq 6000 platform^19^, and sequencing reads were aligned to the human reference genome build GRCh38 using the Burrows–Wheeler Aligner algorithm. Coverage exceeded 20× at 94.6% of sites on average. Variant calls through two separate pipelines, an SPB pipeline that used WeCall (GenomicsPLC) and GLnexus software and a functional equivalence (FE) pipeline, were made available by the UK Biobank for 49,960 samples^19,57,58^. We included variants from the FE pipeline that were also present in the SPB pipeline. We excluded 222 samples for which there were no genotyping data available (*n* = 51) or that failed additional sample quality control using genotyping data: heterozygous missingness outlier (*n* = 112), putative sex chromosome aneuploidy (*n* = 56), and discordance between reported and genetic sex (*n* = 20). There were also 926 individuals excluded due to sample relatedness. The remaining variants on 48,812 unrelated participants were carried forward for further analysis. We converted PLINK-formatted files to VCFs and performed a liftover from GRCh38 to GRCh37.p13^59^.

### Variant quality control

In the 12,852 samples from the coronary artery disease case-control cohort, the analysis was limited to the protein-coding regions and canonical splice sites of three familial hypercholesterolemia genes (*LDLR*, *APOB,* and *PCSK9*). We then filtered the observed variants to a candidate list of variants that excludes synonymous variants or variants present at allele frequency of >0.005 in each racial subpopulation of the gnoMAD Genome Aggregation Database, a publicly available population allele frequency database of 141,456 human exomes and genomes^47^.

Variant quality control for the 19,264 samples from the breast cancer case-control cohort limited the analysis to rare and high-quality variants in the protein-coding regions and canonical splice sites of *BRCA1* and *BRCA2* genes^20^.

In the 48,812 participants form the UK Biobank cohort, the analysis was limited to the protein-coding regions and canonical splice sites of nine genes for any of the three genomic conditions: familial hypercholesterolemia (*LDLR*, *APOB*, and *PCSK9*), hereditary breast and ovarian cancer syndromes (*BRCA1* and *BRCA2*), and Lynch syndrome (*MLH1*, *MSH2*, *MSH6*, and *PMS2*). We filtered the observed variants to a candidate list of variants that excludes synonymous variants or variants present at allele frequency of >0.005 in any racial subpopulation of the gnomAD Genome Aggregation Database^47^. We also performed additional variant quality control filters to exclude variants that fall in low complexity regions, variants that fall in regions with segmental duplications, or variants that did not pass the threshold for the random forest algorithm of gnomAD^47,60^. No individual with more than one pathogenic or likely pathogenic variant was identified in any of the three study populations.

### Variant classification

For the 12,852 coronary artery disease case-control study and the 48,812 UK Biobank cohort, candidate variants were filtered to select variants meeting clinical criteria of pathogenicity (pathogenic or likely pathogenic) based on American College of Medical Genetics and Genomics (ACMG)/Association of Molecular Pathology (AMP) criteria^23^, by an American Board of Genetics and Genomics (AMBGG)-certified clinical geneticist, blinded to the phenotype of the participants, at the Partners HealthCare Laboratory of Molecular Medicine (Boston, MA). In summary, the ACMG/AMP criteria for classifying variant pathogenicity look at the effect of the variant on the gene, any previous reports of pathogenicity of the variant, functional studies supporting the damaging effect of the gene, and the prevalence of the variant in cases with the disease and controls^23^.

Similarly, in the 19,264 breast cancer case-control study from Color Genomics, variant classification was reviewed and signed out by an American Board of Genetics and Genomics (AMBGG)-certified clinical geneticist following criteria for pathogenicity for hereditary breast and ovarian cancer syndromes based on ACMG/AMP criteria^23^.

For each of the three tier 1 genomic conditions, the association of monogenic carrier status by gene with disease was calculated using a logistic regression model with age, sex (except for breast cancer), and the first four principal components of ancestry (Supplementary Figs. 2–4).

### Polygenic score derivation and ancestry correction

We used three previously validated polygenic scores for coronary artery disease, breast cancer and colorectal cancer containing 6,630,150, 3820, and 95 variants, respectively^7,21,22^. Imputed genotype array data available through the UK Biobank was used to calculate the three polygenic scores in all the UK Biobank participants (*n* = 486,477) using the PLINK2.0 score function^52^.

We included individuals of all ancestries and used a previously described method to minimize variance in polygenic score distributions across genetic ancestries. Briefly, we fit a linear regression model using the first four principal components of ancestry to predict each of the three polygenic scores (PS *~* PC1 + PC2 + PC3 + PC4). We then used the residuals from these models as the ancestry-corrected polygenic score and created reference distributions for each phenotype based on the ancestry-corrected scores (Supplementary Fig. 5)^33^. We determined the percentiles for each individual based on these reference distributions for each disease separately.

The breast cancer and colorectal cancer polygenic scores were derived from independent datasets^21,22^. The coronary artery disease polygenic score was derived from the UK Biobank, but it had equal performance in the testing and validation datasets which reassured the absence of model overfitting^7^.

In the breast cancer case-control study of 19,264 participants from Color Genomics, low-coverage whole genome sequencing to a minimum depth of 0.2× was performed and variants were imputed for calculation of the polygenic score^34^. Ancestry-corrected polygenic scores were similarly calculated to minimize variance in score distribution based on genetic ancestry: we fit a linear regression model that uses the first four principal components of ancestry to predict an individual’s raw polygenic score for breast cancer. We then used residuals from this model to create an ancestry reference distribution for each individual (Supplementary Fig. 3). Percentiles were defined based on the distribution of the residuals of the 19,264 participants.

We conducted two sensitivity analyses to test the hypothesis that the risk gradient according to the coronary artery disease polygenic score operated via pathways largely unrelated to cholesterol. First, we removed all variants from the coronary artery disease polygenic score that are within 1 megabase of the three familial hypercholesterolemia genes (*LDLR, APOB*, and *PCSK9*). A total of 14,669 variants were removed and the new score consisted of 6,615,481 variants. We then obtained the odds ratio of disease per standard deviation of the modified score in carriers and noncarriers of familial hypercholesterolemia variants, using a logistic regression model adjusted for age, sex, and the first four principal components of ancestry. Second, we sought to remove the impact of the polygenic score on LDL cholesterol by using the residuals from a linear regression model that additionally included a previously derived polygenic score for LDL cholesterol consisting of 2,013,592 variants (PS_CAD_ *~* PS_LDL_ + PC1 + PC2 + PC3 + PC4)^61^. Similarly for this residualized score, we calculated the odds ratio of coronary artery disease per standard deviation in carriers and noncarriers of rare variants, using a logistic regression model adjusted for age, sex, and the first four principal components of ancestry.

### Assessment of the linearity between polygenic score and disease risk

To evaluate whether the relationships between polygenic scores and disease risk are linearly associated in the 48,812 participants of the UK Biobank, we used two approaches. First, we used a likelihood-ratio test to assess whether including nonlinear terms can better explain the risk model by comparing a logistic regression model with ancestry-corrected polygenic score as a single linear predictor (Model 1) to a polynomial model with additional higher degree of nonlinear terms (square and cube) of ancestry-corrected polygenic score for each phenotype, separately (Model 2) (Supplementary Table 8). Second, we checked model goodness-of-fit by visualizing the predicted risk with the observed risk through the Hosmer–Lemeshow method (Supplementary Fig. 1). We plotted the observed to expected probability of prevalent disease in 20 groups of ancestry-corrected polygenic score percentiles (5% each).

### Statistical analysis

In the coronary artery disease and breast cancer case-control studies, participants were stratified into three groups according to their polygenic score—low, intermediate, or high defined as the lowest quintile, the middle three quintiles, and the highest quintile of the polygenic score distribution, respectively, as we and others have performed previously^62–64^. For carriers and noncarriers in each polygenic score group (coded as an indicator variable with six levels, 2 carrier status × 3 polygenic score groups), the odds ratio for disease was calculated in a logistic regression model with age, sex, and the first four principal components of ancestry as covariates. In the case of breast cancer, only females were included and sex was not a covariate. Non-carriers with intermediate polygenic scores served as the reference group.

The data from the coronary artery disease and breast cancer case-control studies suggested roughly additive effects between monogenic risk variants and polygenic risk, consistent with prior reports^9,11^. Formal tests of interaction were performed by including an interaction term—polygenic score × monogenic mutation carrier status—within logistic regressions models. These results should be interpreted within the context of limited statistical power to detect non-additive interaction given the number of individuals carrying monogenic risk variants. To assess this power, we performed a post hoc power calculation using the R package *waffect*^65^. Within this framework, we fixed the study size and effect size of the polygenic score in noncarriers to the observed values, and subsequently simulated variable polygenic score effect size in the monogenic variant carriers. For coronary artery disease, given an observed odds ratio per standard deviation increase in the polygenic score in noncarriers of 1.74, we had 80% power to detect a different effect size in carriers if the odds ratio was <0.71 or >5.79 at an alpha of 0.05. In this study, the observed odds ratio per standard deviation of polygenic core in monogenic variant carriers was 2.31, corresponding to a *p*-value for interaction of 0.60 (Wald Test). For breast cancer, given an observed odds per standard deviation increase in the polygenic score in noncarriers of 1.57, we had 80% power to detect a different effect size in carriers if the odds ratio was <1.25 or >2.01. In this study, the observed odds ratio per standard deviation of polygenic core in monogenic variant carriers was 1.44, corresponding to a *p*-value for interaction of 0.94 (Wald Test). Within the cohort of 48,812 UK Biobank participants, the *p*-values for interaction were similarly non-significant—0.07, 0.53, and 0.49 (Wald Test) for coronary artery disease, breast cancer, and colorectal cancer, respectively.

Within the cohort of 48,812 UK Biobank participants, to estimate the odds ratio of disease by monogenic variant status and polygenic score percentile for each of the three diseases, we calculated the odds ratio of prevalent disease using a logistic regression model with enrollment age, sex, and the first four principal components of ancestry as covariates—except for breast cancer analyses, which were restricted to females. The predicted odds ratios were then calculated by referencing the risk of noncarriers with median polygenic score and conditioning on the mean value of each other covariate.

A second analysis estimated the probability of disease by age 75 years as a function of monogenic variant carrier status and polygenic score. We fit a Cox proportional hazards model with age as the time-scale, defining the time-to-event as the age at which the diagnosis was first ascertained in cases and the age at the most recent follow-up in controls^66,67^. These models included carrier status, polygenic score, sex, and the first four principal components of ancestry as covariates—except for breast cancer analyses, which were restricted to females. To estimate the probability of disease, we used the monogenic and polygenic effects from the model and standardized the remaining covariates at their mean. The probability of disease by time *t* was estimated by *F*(*t*) = 1−*S*(*t*), where *S*(*t*) is the survivor function, estimated by the *survfit* function from the R *survival* package.

Statistical analyses were performed using R software, version 3.5 (R Project for Statistical Computing). Statistical significance was set at *p* < 0.05, and two-sided *p* values were used.

### Reporting summary

Further information on research design is available in the Nature Research Reporting Summary linked to this article.

## Supplementary information

Supplementary Information

Reporting Summary

## Data Availability

Phenotypes derived as part of this manuscript—including calculated polygenic scores, monogenic variant carrier status, and disease status endpoints—will be returned to the UK Biobank for dissemination to approved investigators. Further information on obtaining approval for access to the UK Biobank data is available at: https://www.ukbiobank.ac.uk/researchers. We also included Supplementary Table 11 with the codings of all phenotypes so that they can be replicated by other investigators from raw phenotypes in the UK Biobank. Criteria used to support pathogenicity assessment for monogenic risk variants are provided in Supplementary Tables 1 and 5. The raw weights for calculating the coronary artery disease polygenic score are available for download from the Broad Institute Cardiovascular Disease Knowledge Portal (http://www.broadcvdi.org). The raw weights for calculating the breast cancer and colorectal cancer polygenic scores are available from the original publications^21,22^. Aggregate summaries of the Color Genomic data are available at: https://data.color.com. The Genome Aggregation Database (gnoMAD) is publicly available at: http://gnomad.broadinstitute.org.
