# Supplementary material for: Polygenic background modifies penetrance of monogenic variants for tier 1 genomic conditions
Source: Nat Commun. 2020 Aug 20;11:3635. doi: 10.1038/s41467-020-17374-3 (PMC7441381; doi:10.1038/s41467-020-17374-3)
Supplement: Supplementary file 1 — Supplementary Information [file 41467_2020_17374_MOESM1_ESM.pdf]

Supplementary Information

Fahed, *et al*, "Polygenic background modifies penetrance of monogenic variants for tier 1 genomic conditions"

Supplementary Figures

Supplementary Figure 1: Plots of observed vs. predicted risk of disease using the Hosmer-Lemeshow method

Supplementary Figure 2: Association of pathogenic or likely pathogenic FH carrier status with risk of coronary artery disease stratified by gene

Supplementary Figure 3: Association of pathogenic or likely pathogenic HBOC carrier status with risk of breast cancer stratified by gene

Supplementary Figure 4: Association of pathogenic or likely pathogenic Lynch variant carrier status with risk of colorectal cancer stratified by gene

Supplementary Figure 5: Distributions of the polygenic score across racial groups in the UK Biobank

Supplementary Figure 6: Distributions of the breast cancer polygenic score across racial groups in the Color genomics study

Supplementary Tables

Supplementary Table 1: Evidence in support of pathogenic or likely pathogenic assertions for familial hypercholesterolemia variants in the 12,852 coronary artery disease case-control study participants derived from UK Biobank

Supplementary Table 2: Effect of the polygenic score among carriers and noncarriers of monogenic risk variants in the case-control studies

Supplementary Table 3: Odds ratio of disease comparing carriers of monogenic risk variants with high vs. low polygenic score

Supplementary Table 4: Effect of non-cholesterol polygenic background on risk of coronary artery disease in familial hypercholesterolemia

Supplementary Table 5: Evidence in support of pathogenic or likely pathogenic assertions for three genomic conditions in 48,812 UK Biobank participants

Supplementary Table 6: Baseline characteristics of the 48,812 UK Biobank participants

Supplementary Table 7: Baseline characteristics of the 48,812 UK Biobank participants by coronary artery disease status

Supplementary Table 8: Likelihood-ratio test for linearity assessment of polygenic score-disease relationships

Supplementary Table 9: Baseline characteristics of the 26,597 female UK Biobank participants by breast cancer status

Supplementary Table 10: Baseline characteristics of the 48,812 UK Biobank participants by colorectal cancer status

Supplementary Table 11: Phenotype definitions of coronary artery disease, breast cancer, and colorectal cancer in the cohort study from the UK Biobank

Supplementary Figure 1: Plots of observed vs. predicted risk of disease using the Hosmer-Lemeshow method

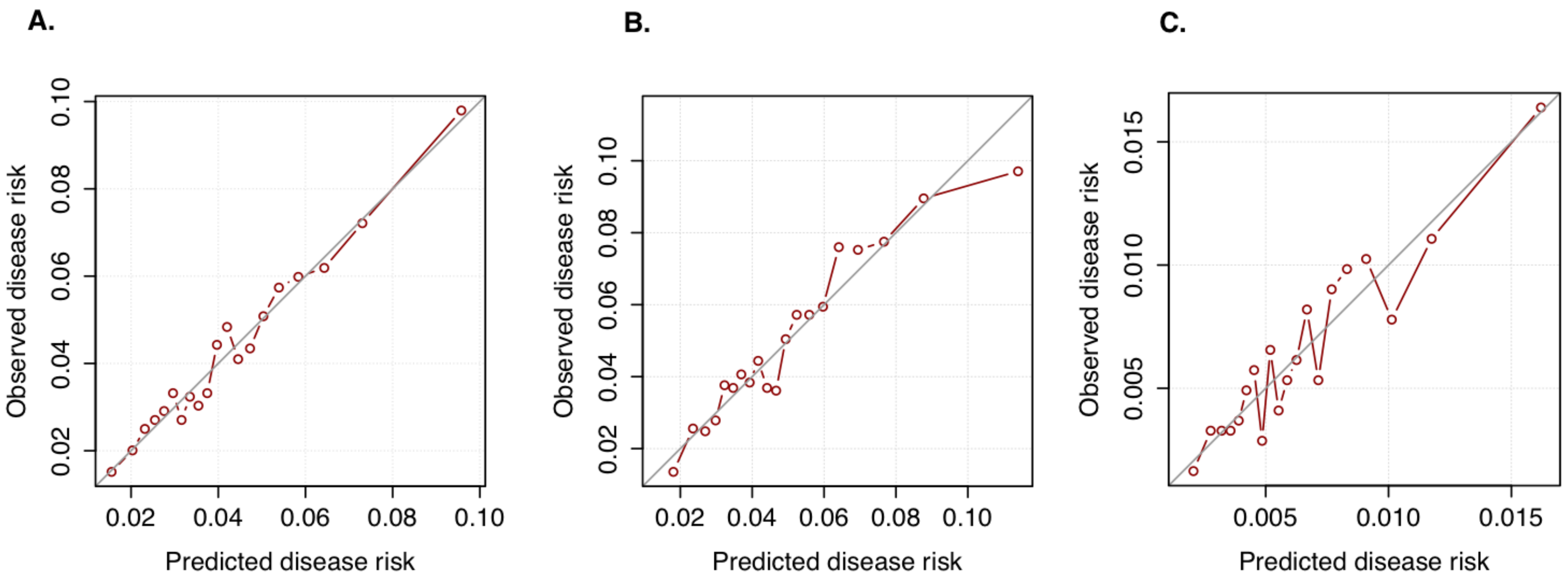

For UK Biobank participants (N=48,812), shown here is model goodness-of-fit evaluation of polygenic background to disease risk by Hosmer-Lemeshow method for **A.** coronary artery disease, **B.** breast cancer, and **C.** colorectal cancer. A single linear predictor of ancestry-corrected polygenic risk score was used to predict the disease risk by a logistic regression model for each disease separately. Each plot shows the observed to predicted probability of disease in 20 groups of polygenic score percentiles (5% each).

Supplementary Figure 2: Association of pathogenic or likely pathogenic FH carrier status with risk of coronary artery disease stratified by gene

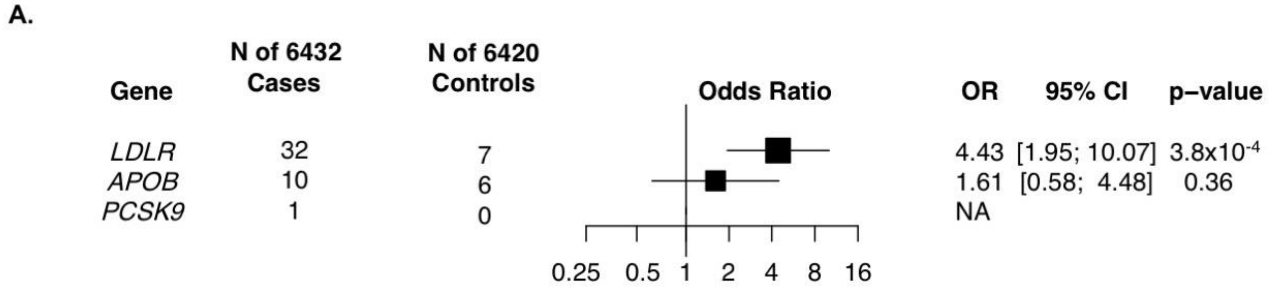

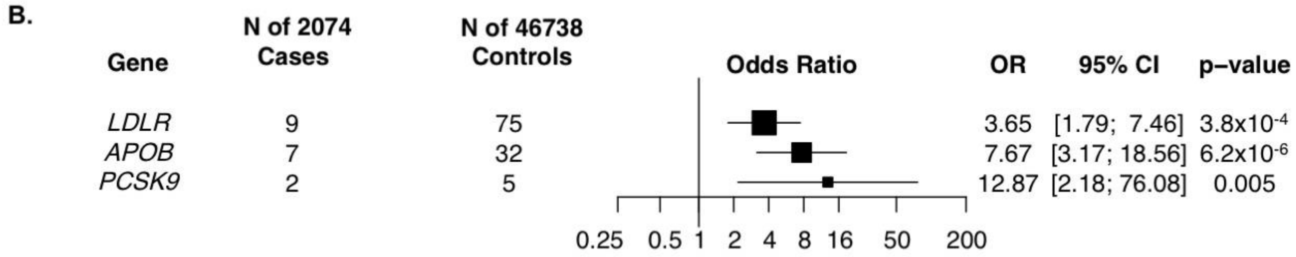

In both **A.** case-control study (N=12,852) and **B.** cohort study (N=48,812), carriers were defined as individuals with a variant meeting clinical criteria of pathogenicity (pathogenic or likely pathogenic) based on American College of Medical Genetics and Genomics (ACMG)/Association of Molecular Pathology (AMP) criteria as reviewed by a certified clinical geneticist blinded to the phenotype. The odds ratio (OR) was assessed in a logistic regression model with age, sex, and the first four principal components of ancestry as covariates. The meta-analysis was conducted using the R *meta* package. The p-value for the test of heterogeneity was 0.13 in the case-control study (A) and 0.26 in the cohort study (B). The black boxes indicate the odds ratio. The horizontal lines around the black boxes indicate the 95% confidence intervals. p-values in the figure were estimated by the Wald Test. Statistical significance was set at $p < .05$, and 2-sided p values were used. Abbreviations: FH familial hypercholesterolemia, *LDLR* low density lipoprotein receptor, *APOB* apolipoprotein B, *PCSK9* proprotein convertase subtilisin/kexin type 9.

Supplementary Figure 3: Association of pathogenic or likely pathogenic HBOC carrier status with risk of breast cancer stratified by gene

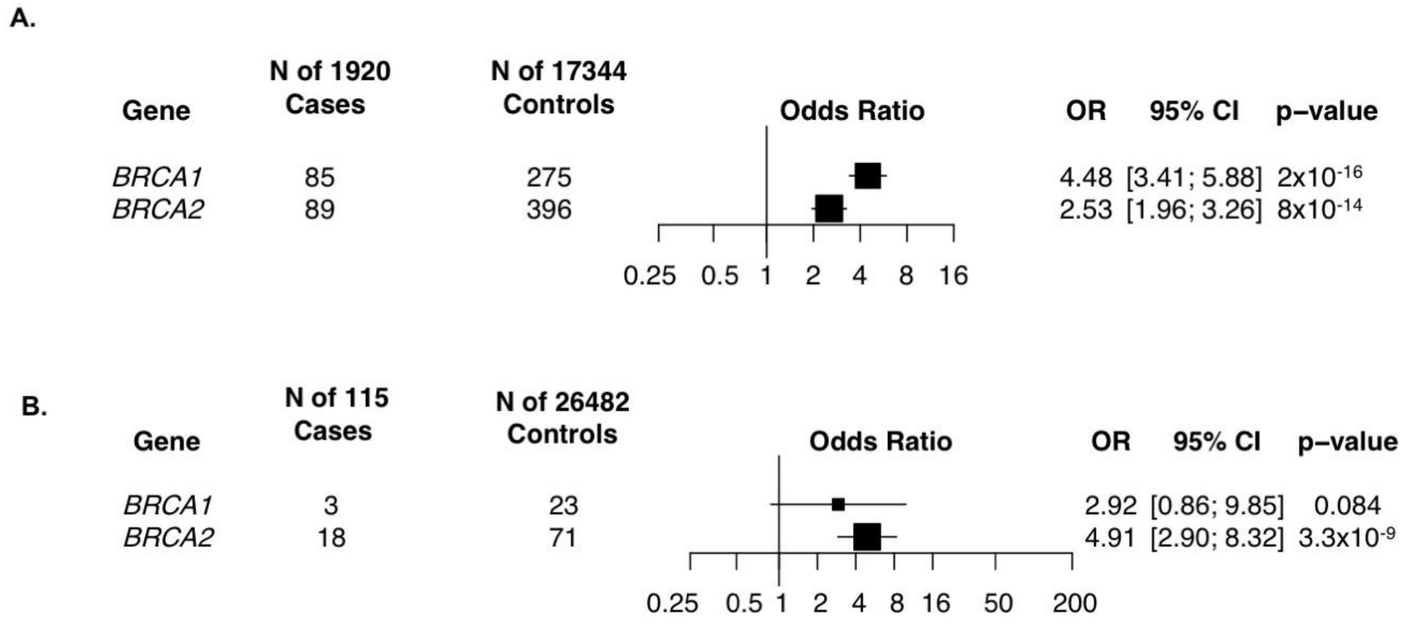

In both **A.** case-control study (N=19,264) and **B.** cohort study (N=26,597), carriers were defined as individuals with a variant meeting clinical criteria of pathogenicity (pathogenic or likely pathogenic) based on American College of Medical Genetics and Genomics (ACMG)/Association of Molecular Pathology (AMP) criteria as reviewed by a certified clinical geneticist blinded to the phenotype. The odds ratio (OR) was assessed in a logistic regression model with age and the first four principal components of ancestry as covariates. The meta-analysis was conducted using the R *meta* package. The p-value for the test of heterogeneity was 0.0026 in the case-control study and 0.44 in the cohort study. The black boxes indicate the odds ratio. The horizontal lines around the black boxes indicate the 95% confidence intervals. p-values in the figure were estimated by the Wald Test. Statistical significance was set at $p < .05$, and 2-sided p-values were used.

Supplementary Figure 4: Association of pathogenic or likely pathogenic Lynch variant carrier status with risk of colorectal cancer stratified by gene (cohort study, N=48,812)

Gene	N of 315 Cases	N of 48497 Controls	OR	95% CI	p–value
MLH1	9	10	167.36	[63.66; 439.99]	$3x10^{-25}$
MSH2	1	5	36.34	[4.09; 322.89]	0.001
MSH6	1	42	3.93	[0.54; 28.83]	0.178
PMS2	0	8	NA		

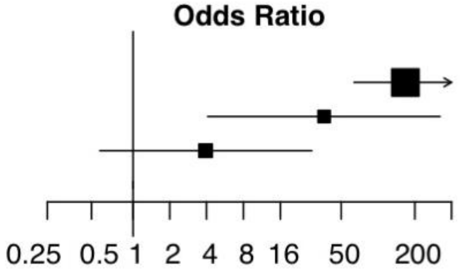

Carriers were defined as individuals with a variant meeting clinical criteria of pathogenicity (pathogenic or likely pathogenic) based on American College of Medical Genetics and Genomics (ACMG)/Association of Molecular Pathology (AMP) criteria as reviewed by a certified clinical geneticist blinded to the phenotype. The odds ratio (OR) was assessed in a logistic regression model with age, sex, and the first four principal components of ancestry as covariates. The meta-analysis was conducted using the R *meta* package. The p-value for the test of heterogeneity was 0.0094. The black boxes indicate the odds ratio. The horizontal lines around the black boxes indicate the 95% confidence intervals. p-values in the figure were estimated by the Wald Test. Statistical significance was set at $p < .05$, and 2-sided p-values were used.

Supplementary Figure 5: Distributions of the polygenic score across racial groups in the UK Biobank

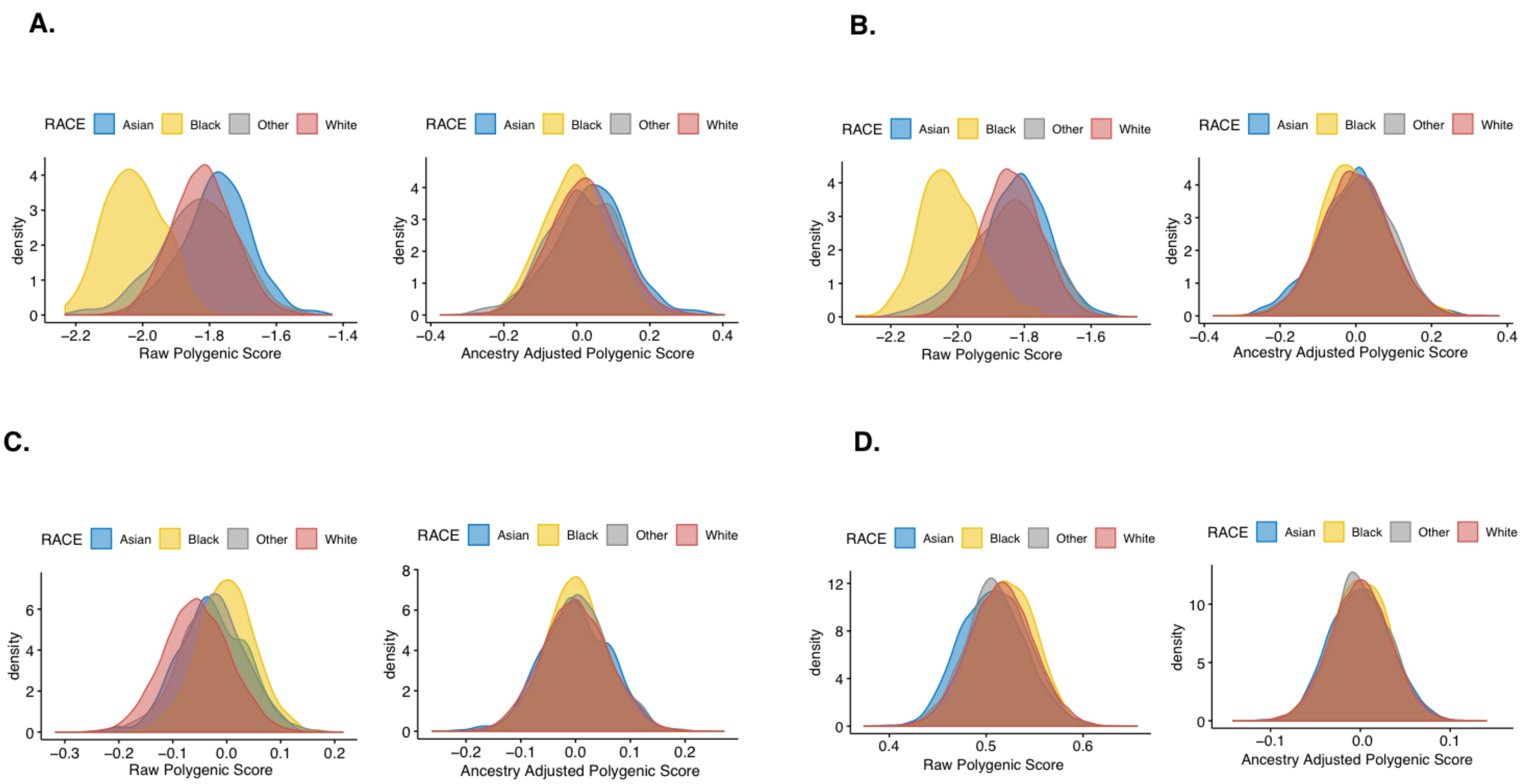

Raw values (left) and after adjustment for genetic ancestry using the first 4 principal components (right) are shown for the **A.** coronary artery disease polygenic score in the case-control study (N=12,852), **B.** coronary artery disease polygenic score in the UK Biobank cohort (N=48,812), **C.** Breast cancer polygenic score in the UK Biobank cohort (N=48,812), and **D.** Colorectal cancer polygenic score in the UK Biobank cohort (N=48,812). Ancestry-adjusted values were scaled to a mean of 0 and SD of 1 to facilitate interpretation.

Supplementary Figure 6: Distributions of the breast cancer polygenic score across racial groups in the Color genomics study

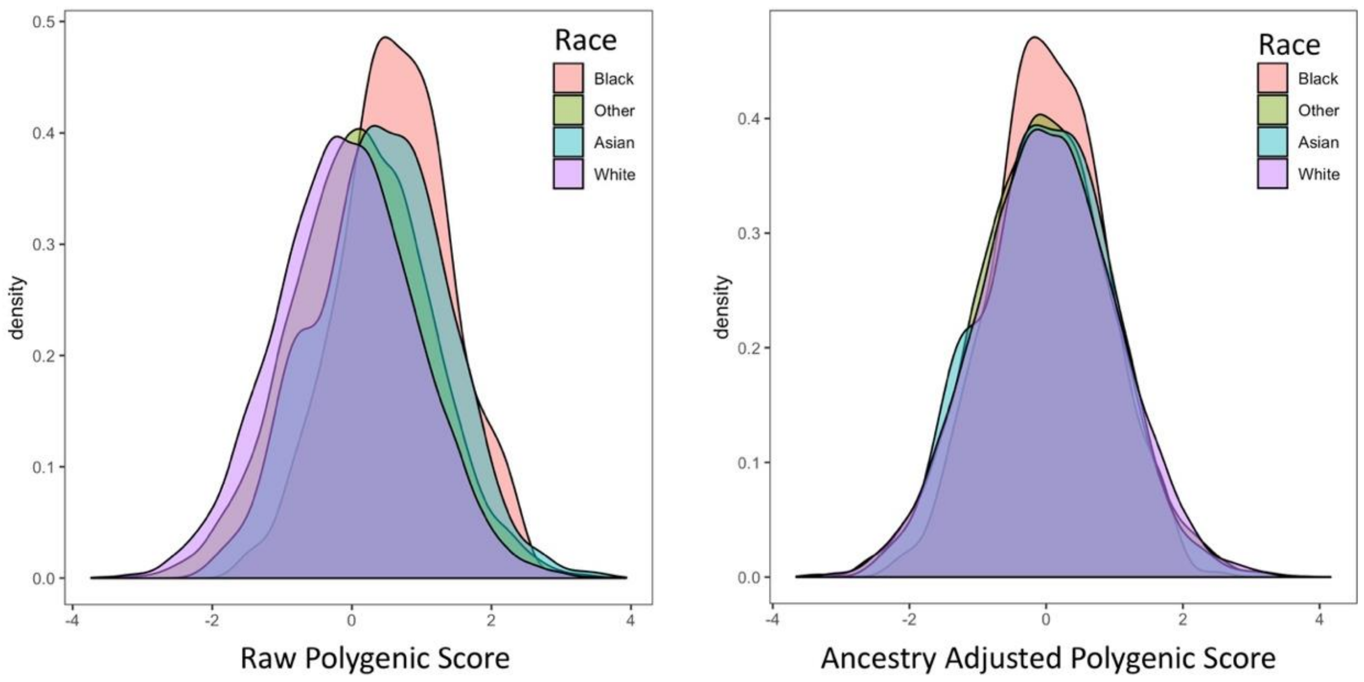

Raw values (left) and after adjustment for genetic ancestry using the first 4 principal components (right) are shown for participants in the Color genomics study (N=19,264). Values were scaled to a mean of 0 and SD of 1 to facilitate interpretation.

Supplementary Table 1: Evidence in support of pathogenic or likely pathogenic assertions for familial hypercholesterolemia variants in the 12,852 coronary artery disease case-control study participants derives from UK Biobank

Variant	Gene (Variant Type)	Amino acid or cDNA change	Number of carriers	Evidence in Support of Pathogenicity Assessment
2:21229161:G>A	*APOB* Missense	p.Arg3527Trp	1	The p.Arg3527Trp variant (also referred to in the literature as p.Arg3500Trp) in APOB has been reported in at least 33 individuals with familial hypercholesterolemia, the majority of whom are of East Asian ancestry, and segregated with disease in at least 15 affected relatives from 4 families (Gaffney 1995, Choong 1997, Tai 1998, Fisher 1999, Tai 2001, Yang 2007, Hollandt 2012, Chiou 2010, Chiou 2011, Chiou 2012, Bertolini 2013). This variant has been reported in ClinVar (Variation ID 40223) and has also been identified in 22/18848 East Asian chromosomes by the Genome Aggregation Database (gnomAD, http://gnomad.broadinstitute.org; dbSNP rs144467873). This frequency is low enough to be consistent with the frequency of familial hypercholesterolemia (FH) in the general population. In vitro functional studies provide some evidence that the p.Arg3527Trp variant may impact protein function (Gaffney 1995, Fisher 1999, Tai 2001). Additionally, another variant at this position, p.Arg3527Gln, is a well-established pathogenic variant for FH. In summary, this variant meets criteria to be classified as pathogenic for FH in an autosomal dominant manner based upon segregation studies, increased prevalence in affected individuals, and pathogenicity of other variants at this position. Please note that pathogenic variants in APOB can have reduced penetrance and a less severe phenotype than disease-causing LDLR or PCSK9 variants (Youngblom and Knowles, GeneReviews). ACMG/AMP Criteria applied: PS4; PP1_Strong; PM5; PS3_Supporting.
2:21229160:C>T	*APOB* Missense	p.Arg3527Gln	15	The p.Arg3527Gln variant in APOB is a well-established pathogenic variant that is mainly found in individuals of European descent. It has been previously reported in >500 individuals with familial hypercholesterolemia (FH) and segregated with disease in >50 affected relatives (Soria 1989, März 1993, Leren 1997, Ludwig 1990, Bednarska-Makaruk 2001, Horvath 2001, Kalina 2001). It has also been reported by other clinical laboratories in ClinVar (Variation ID 17890) and has been identified in 53/126056 of European chromosomes, including 1 homozygote, by gnomAD (http://gnomad.broadinstitute.org/). This frequency is low enough to be consistent with the frequency of FH in the general population. In summary, this variant meets criteria to be classified as pathogenic for autosomal dominant familial hypercholesterolemia based upon presence in multiple affected individuals and segregation studies. Please note that pathogenic variants in APOB can have reduced penetrance and a less severe phenotype than disease-causing LDLR or PCSK9 variants (Youngblom and Knowles, GeneReviews). ACMG/AMP Criteria applied: PS4_Strong; PP1_Strong.
19:11213390:C>T	*LDLR* Missense	p.Arg81Cys	1	The p.Arg81Cys variant in LDLR (also described as p.Arg81Cys in the literature) has been reported in >18 individuals with familial hypercholesterolemia (FH; Nissen 1998, Loubser 1999, Fouchier 2001, Bourbon 2008, Alonso 2009, Huijgen 2010, Huijgen 2011, Bertolini 2013), though not all individuals had extremely elevated LDL-cholesterol levels (Huijen 2011). It has been suggested that the p.Arg81Cys variant results in an LDLR protein that does not function as effectively as that produced by the wild-type allele, thus resulting in only modest LDL-cholesterol elevations (Huijgen 2010). This variant has also been reported by other clinical laboratories in ClinVar (Variation ID: 183083) and has been identified in 2/111718 European chromosomes by the Genome Aggregation Database (gnomAD, http://gnomad.broadinstitute.org; dbSNP rs730882078). This frequency is low enough to be consistent with the frequency of FH in the general population. Computational prediction tools and conservation analysis do not provide strong support for or against an impact to the protein. In summary, although additional studies are required to fully establish its clinical significance, the p.Arg81Cys variant is **likely** pathogenic. ACMG/AMP Criteria applied: PS4_Strong, PM2.
19:11213450:G>A	*LDLR* Missense	p.Glu101Lys	1	The p.Glu101Lys variant in LDLR has been reported in the heterozygous state in >30 individuals with familial hypercholesterolemia (FH), in the compound heterozygous state in 1 individual with homozygous FH (Loux 1992, Webb 1992, García-García 2001, Mozas 2004, Humphries 2006, Miyakem2009, Taylor 2010, Futema 2013, Do 2015) and segregated with disease in four affected relatives from two families (Webb 1992, Loux 1992). This variant has also been reported by other clinical laboratories in ClinVar (Variation ID# 161266). In vitro functional studies provide some evidence that the p.Glu101Lys variant may impact protein function (Webb 1992). This variant has also been identified in 3/111700 European chromosomes by the genome Aggregation Database (gnomAD, http://gnomad.broadinstitute.org). This frequency is low enough to be consistent with the frequency of FH in the general population. Computational prediction tools and conservation analysis suggests that the p.Glu101Lys variant may impact the protein, though this information is not predictive enough to

				determine pathogenicity. In summary, this variant meets criteria to be classified as pathogenic for familial hypercholesterolemia in an autosomal dominant manner based upon presence in multiple affected individuals, segregation studies, low frequency in the general population and functional evidence. ACMG/AMP Criteria applied: PS4, PM2, PS3_Moderate, PP1, PP3.
19:11216084:G>A	*LDLR* Missense	p.Asp168Asn	1	The p.Asp168Asn variant in LDLR has been reported in at least 8 individuals with familial hypercholesterolemia (FH) and in 2 individuals with early-onset myocardial infarction (Day et al. 1997, Lee et al. 1998, Punzalan et al. 2005, Do et al., 2015, ClinVar: Variation ID 183136). In vitro functional studies provide some evidence that the p.Asp168Asn variant may cause a decrease in LDL uptake and binding (Etxebarria 2015). This variant has also been identified in 2/111620 European chromosomes by the Genome Aggregation Database (GnomAD, http://gnomad.broadinstitute.org; dbSNP rs200727689). Computational prediction tools and conservation analysis suggest that the p.Asp168Asn variant may impact the protein. Additionally, other variants at this position have been reported in association with FH in the Human Gene Mutation Database (HGMD; Stenson et al. 2017). In summary, although additional studies are required to fully establish its clinical significance, the p.Asp168Asn variant is likely pathogenic. ACMG/AMP Criteria applied: PS4_Moderate, PS3_Supporting, PM2, PP3.
19:11216133:G>A	*LDLR* Missense	p.Cys184Tyr	1	The p.Cys184Tyr variant in LDLR (also described as p.Cys163Tyr in the literature) has been reported in >15 individuals with familial hypercholesterolemia (FH) and segregated with disease in at least 11 affected relatives from 4 families (Lee 1998, Graham 1999, Fouchier 2001, Wang 2001, Bourbon 2008, Jannes 2015, Martin 2016). It has also been reported by other clinical laboratories in ClinVar (Variation ID 3739). This variant has been identified in 3/15004 European chromosomes by the Genome Aggregation Database (gnomAD, http://gnomad.broadinstitute.org/; dbSNP rs121908039). This frequency is low enough to be consistent with the frequency of FH in the general population. Computational prediction tools and conservation analysis suggest that the p.Cys184Tyr variant may impact the protein. In summary, this variant meets criteria to be classified as pathogenic for FH in an autosomal dominant manner based upon presence in multiple affected individuals, segregation studies, very low frequency in the general population and computational evidence. ACMG/AMP Criteria applied: PS4, PP1_Strong, PM2, PP3.
19:11216244:A>G	*LDLR* Missense	p.Asp221Gly	1	The p.Asp221Gly variant in LDLR has been reported in >75 individuals with familial hypercholesterolemia (FH), including in 4 homozygotes who presented with more severe disease (Hobbs 1992, Chmara 2010, Bertolini 2013). However, not all individuals carrying this variant presented with high cholesterol levels (Bertolini 2013, Thormaehlen 2015). This variant has also been reported by other clinical laboratories in ClinVar (Variation ID 183092) and has been identified in 13/111132 European chromosomes by the Genome Aggregation Database (gnomAD, http://gnomad.broadinstitute.org; dbSNP rs373822756). Please note that this frequency is low enough to be consistent with the frequency of FH in the general population. In vitro functional studies provide some evidence that the p.Asp221Gly variant may impact protein function (Thormaehlen 2015). In summary, this variant meets criteria to be classified as pathogenic for familial hypercholesterolemia in an autosomal dominant manner based upon presence in affected individuals, low frequency in the general population, computational and functional evidence. ACMG/AMP Criteria applied: PS4, PM3_Strong, PP3, PS3_Supporting.
19:11216262:AC>*	*LDLR* Frameshift	p.Asp227Glyfs*12	1	The p.Asp227Glyfs*12 variant in LDLR has been reported in at least 9 individuals with hypercholesterolemia (Gudnason 1993, Graham 1999, Bunn 2002, Dedoussis 2004, Martin 2016) and has also been reported in ClinVar (Variation ID #3731). This variant has also been identified in 1/110684 European chromosomes by the Genome Aggregation Database (gnomAD, http://gnomad.broadinstitute.org; dbSNP rs387906305). This variant is predicted to cause a frameshift, which alters the protein's amino acid sequence beginning at position 227 and leads to a premature termination codon 12 amino acids downstream. This alteration is then predicted to lead to a truncated or absent protein. Heterozygous loss of function of the LDLR gene is an established disease mechanism in familial hypercholesterolemia (FH). In summary, this variant meets criteria to be classified as pathogenic for FH in an autosomal dominant manner based upon the predicted impact to the protein, presence in multiple affected individuals and low frequency in controls. ACMG/AMP Criteria applied: PVS1, PM2, PS4_Moderate.
19:11216263:C>G	*LDLR* Missense	p.Asp227Glu	1	The p.Asp227Glu variant in LDLR (also known as the FH Afrikaner 1 allele) is a founder variant in the Afrikan population and is thought to account for 65 - 75% of familial hypercholesterolemia in Afrikans (Leitersdorf 1989, Kotze 1990, Kotze 1994). This variant has also been identified in at least 7 Caucasian individuals with familial hypercholesterolemia (Gudnason 1993, Callis 1998, Fouchier 2001, Bertolini 2013, Sharifi 2016) and has been reported in ClinVar (Variation ID 3690). Additionally, in vitro functional studies provide some evidence that the p.Asp227Glu variant may impair receptor activity (Fourie 1988). This variant has been identified in 1/33548 Latino chromosomes by the Genome Aggregation Database (gnomAD; http://gnomad.broadinstitute.org; dbSNP rs121908028). This frequency is low enough to be consistent with the frequency of familial hypercholesterolemia (FH) in the general population. In summary, this variant meets criteria to be classified as pathogenic for FH in an

				autosomal dominant manner based upon low frequency in controls, functional evidence, and presence in multiple affected individuals. The ACMG/AMP Criteria applied: PS4, PP1_Strong, PM2, PP3, PS3_Supporting.
19:11216264:G>T	*LDLR* Premature stop	p.Glu228*	2	The p.Glu228* variant in LDLR (also described as p.Glu207* in the literature) has been reported in 23 individuals with familial hypercholesterolemia (FH; Hobbs 1992, Nauck 2001, Bodamer 2002, Kim 2004, Taylor 2007, Hola 2009, Vandrovcova 2013, Han 2015, Du 2016, Abul-Husn 2016) and segregated with disease in 6 affected relatives from 3 families (Bodamer 2002, Kim 2004). This variant has also been reported by other clinical laboratories in ClinVar (Variation ID 226333). In vitro functional studies provide some evidence that the p.Glu228* variant may impact protein function (Hobbs 1992, Holla 2009). This variant has also been identified in 1/23818 African and 2/125438 European chromosomes by the Genome Aggregation Database (gnomAD, http://gnomad.broadinstitute.org; dbSNP rs12190829). This frequency is low enough to be consistent with the frequency of FH in the general population. This nonsense variant leads to a premature termination codon at position 228, which is predicted to lead to a truncated or absent protein. Heterozygous loss of function of the LDLR gene is an established disease mechanism in individuals with FH. In summary, this variant meets criteria to be classified as pathogenic for FH in an autosomal dominant manner based upon presence in multiple affected individuals, segregation studies, low frequency in the general population, predicted impact on the protein, and functional evidence. ACMG/AMP criteria applied: PVS1; PM2; PS4; PP1_Moderate; PS3_Supporting.
19:11217264:G>A	*LDLR* Missense	p.Glu240Lys	1	The p.Glu240Lys variant in LDLR (also described as p.Gly219Lys in the literature) has been reported in 6 individuals with familial hypercholesterolemia (FH) and segregated with disease in 3 affected relatives from two families (Fouchier 2005, Bertolini 2013, Hobbs 1992, Mollaki 2014, Norsworthy 2014). However, this variant has also been reported in one individual with normal cholesterol levels (Abul-Husn 2016), suggesting reduced penetrance. In vitro functional studies provide some evidence that the p.Glu240Lys variant may impact protein folding and transport of the mature protein from the ER to the Golgi (Hobbs 1992, North 2000, North 2001). This variant has also been reported by other clinical laboratories in ClinVar (Variation ID 200920) and has been identified in 7/126728 European chromosomes by the Genome Aggregation Database (gnomAD, http://gnomad.broadinstitute.org/; dbSNP 7685633000). This frequency is consistent with the frequency of FH in the general population. Computational prediction tools and conservation analysis do not provide strong support for or against an impact to the protein. In summary, although additional studies are required to fully establish its clinical significance, the p.Glu240Lys variant is likely pathogenic. ACMG/AMP Criteria applied: PM2; PS3_Supporting; PS4_Moderate; PP1.
19:11218188:G>A	*LDLR* Missense	p.Cys313Tyr	1	The p.Cys313Tyr variant in LDLR (also described as p.Cys292Tyr in the literature) has been reported in >10 individuals with familial hypercholesterolemia (FH), of which 2 are in the compound heterozygous state (Day 1997, Thiart 2000, Fouchier 2001, Bunn 2002, Van der Graaf 2011, Martin 2016). It has also been reported in ClinVar (Variation ID: 226339) and was absent from large population studies. Computational prediction tools and conservation analysis suggest that the p.Cys131Tyr variant may impact the protein. This variant is located in the last three bases of the exon, which is part of the 5' splice region. Computational tools do not suggest an impact to splicing. Additionally, other missense variants at this amino acid position (p.Cys313Arg, p.Cys313Gly and p.Cys313Trp) have been reported in individuals with familial hypercholesterolemia (Human Gene Mutation Database: Stenson 2017), suggesting that changes at this position may not be tolerated. In summary, although additional studies are required to fully establish its clinical significance, the p.Cys313Tyr variant is likely pathogenic. ACMG/AMP Criteria applied (Richards 2015): PS4_Moderate, PM2, PP3, PM5_Supporting.
19:11221414:G>A	*LDLR* Missense	p.Gly343Ser	2	The p.Gly343Ser variant in LDLR has been reported in at least 10 individuals with familial hypercholesterolemia (FH); nine in the heterozygous state (Fouchier 2001, Bourbon 2008, Reshef 1996, Thiart 2000, Guardamagna 2009, Mozas 2004, Van Gaal 2001, Salazar 2002) and one in the compound heterozygous state (Hobbs 1992). This variant has also been reported by other clinical laboratories in ClinVar (Variation ID 183106). In vitro functional studies provide evidence that the p.Gly343Ser variant may impact protein function (Hobbs 1992). This variant has been identified in 5/126,580 European chromosomes by gnomAD (http://gnomad.broadinstitute.org/). This frequency is low enough to be consistent with the frequency of FH in the general population. Computational prediction tools and conservation analysis suggest that the p.Gly343Ser variant may impact the protein, though this information is not predictive enough to determine pathogenicity. In summary, although additional studies are required to fully establish its clinical significance, the p.Gly343Ser variant meets criteria to be classified as likely pathogenic for FH in an autosomal dominant manner based upon presence in multiple affected individuals, low frequency in controls, and functional and computational evidence. ACMG/AMP Criteria applied: PS4_Moderate, PM2, PP3, PS3_supporting.
19:11224013:C>T	*LDLR* Missense	p.Arg416Trp	1	The p.Arg416Trp variant in LDLR has been reported in >30 individuals with familial hypercholesterolemia (FH) and segregated with disease in 1 affected relative (Day 1997, Dušková 2011, Tichý 2012, Bertolini 2013, Do 2015, Etxebarria 2015, Han 2015, Sánchez-Hernández 2016, Sharifi 2016, Durst 2017). It has also been

				reported by other clinical laboratories in ClinVar (Variation ID: 183110) and been identified in 3/30782 South Asian chromosomes by the Genome Aggregation Database (gnomAD, http://gnomad.broadinstitute.org/; dbSNP rs570942190). This frequency is low enough to be consistent with the frequency of FH in the general population. Amino acid position 416 is a known mutation hotspot, with many other variants (p.Arg416Pro, p.Arg416Leu and p.Arg416Gln) reported in individuals with FH (Liguori 2001, Bertolini 2013, Chiou 2010, Thiart 1998, Huijgen 2012). Functional studies provide some evidence that the p.Arg416Trp variant may impact protein function (Etxebarria 2015). In summary, this variant meets criteria to be classified as pathogenic for FH in an autosomal dominant manner based upon presence in multiple affected individuals, low frequency in the general population, functional and computational evidence and its presence in a known mutational hotspot. ACMG/AMP criteria applied: PS4, PM1, PM2, PS3_Moderate, PP3
19:11213408:T>G	*LDLR* Missense	p.Trp87Gly	1	The p.Trp87Gly variant in LDLR is a well-established pathogenic variant for familial hypercholesterolemia (Leitersdorf 1990, Jensen 1996, Vohl 1997, Tybjaerg-Hansen 2005, Futema 2013), and is a known founder mutation in the French Canadian population where it has been reported in >400 individuals with FH, including >15 homozygous individuals (Vohl 1997). It has also been identified in 5/66740 European chromosomes by the Exome Aggregation Consortium (ExAC, http://exac.broadinstitute.org; dbSNP rs121908025); however, this frequency is low enough to be consistent with the frequency of FH in the general population. Additionally, in vitro functional studies provide some evidence that the p.Trp87Gly variant may impact protein function (Leitersdorf 1990). In summary, this variant meets our criteria to be classified as pathogenic for FH in an autosomal dominant manner based upon its identification in a large number of affected individuals and low frequency in controls.
19:11224266:G>T	*LDLR* Missense	p.Asp472Tyr	1	The p.Asp472Tyr variant in LDLR (also described as p.Asp451Tyr in the literature) has been reported in 10 individuals with familial hypercholesterolemia (FH) and 12 individuals who had a myocardial infarction, and segregated with disease in 3 affected relatives from 2 families (Abul-Husn 2016, Vohnout 2016, Thormaehlen 2015, Tichy 2012, Bertolini 2013, Do 2015, Campagna 2008). This variant has also been reported by other clinical laboratories in ClinVar (Variation ID 183116) and has been identified in 5/30778 of South Asian chromosomes and 8/126504 European chromosomes by the Genome Aggregation Database (gnomAD, http://gnomad.broadinstitute.org/; dbSNP 730882102). This frequency is low enough to be consistent with the frequency of FH in the general population. Computational prediction tools and conservation analysis do not provide strong support for or against an impact to the protein and in vitro functional assays were unclear in their overall impact (Thormaehlen 2015). In summary, although additional studies are required to fully establish its clinical significance, the p.Asp472Tyr variant is likely pathogenic. ACMG/AMP Criteria applied (Richards 2015): PS4, PM2, PP1.
19:11230819:C>T	*LDLR* Missense	p.Arg633Cys	3	The p.Arg633Cys variant in LDLR has been reported in at least 11 individuals with familial hypercholesterolemia (FH), including one homozygote (Day 1997, Mozas 2004, Guardamagna 2009, Taylor 2009, Chiou 2011, Tichy 2012, Bertolini 2013, Xiang 2017, Medieros 2017). This variant has been reported in ClinVar (Variation ID: 226379) and has also been identified in 1/30782 South Asian chromosomes by the Genome Aggregation Database (gnomAD, http://gnomad.broadinstitute.org). Computational prediction tools and conservation analysis suggest that the p.Arg833Cys variant may impact the protein. Two other variants at this amino acid position have been reported in at least 4 individuals with FH (p.Arg633Leu and p.Arg633His). In summary, although additional studies are required to fully establish its clinical significance, the p.Arg633Cys variant is likely pathogenic. ACMG/AMP Criteria applied: PS4_Moderate, PM2, PM5_Supporting, PP3.
19:11231112:C>T	*LDLR* Missense	p.Pro685Leu	3	The p.Pro685Leu variant in LDLR, also described as p.Pro664Leu in the literature, has been reported in >40 individuals with familial hypercholesterolemia (FH), segregated with disease in >40 affected relatives from at least 4 families, and was identified in the homozygous state in at least 10 individuals with FH (Bertolini 2013, Sharifi 2016, Medeiros 2010,Rubinsztein 1992, Soutar 1989, Soutar 1991, Thormaehlen 2015, Van Der Graaf 2011). This variant has also been reported by other clinical laboratories in Clinvar (Variation ID 3702) and was identified in 6/126656 European chromosomes by the Genome Aggregation Database (gnomAD, http://gnomad.broadinstitute.org; dbSNP rs28942084). However, this frequency is low enough to be consistent with the frequency of FH in the general population. In vitro functional studies provide some evidence that the p.Pro685Leu variant may impact protein function (Knight 1989, Rubinsztein 1992, Thormaehlen 2015). In summary, this variant meets criteria to be classified as pathogenic for familial hypercholesterolemia in an autosomal dominant manner based upon presence in multiple affected individuals, segregation studies, low frequency in controls and functional evidence. ACMG/AMP Criteria applied: PS4, PP1_Strong, PM2, PS3_Supporting.
19:11215919:G>A	*LDLR* Missense	p.Glu113Lys	4	The p.Glu113Lys variant in LDLR has been reported in 2 individuals with hypercholesterolemia, 1 individual with probable hypercholesterolemia, and segregated with disease in 7 affected individuals from 1 family (Wu 2000, Fouchier 2005, Taylor 2007). It has also been identified in 0.002% (3/128566) of European chromosomes by gnomAD (http://gnomad.broadinstitute.org) and is reported in ClinVar (Variation ID: 237872). Computational

				prediction tools and conservation analysis suggest that this variant may not impact the protein, though this information is not predictive enough to rule out pathogenicity. In summary, although additional studies are required to fully establish its clinical significance, this variant meets criteria to be classified as likely pathogenic for autosomal dominant hypercholesterolemia. ACMG/AMP Criteria applied: PP1_Strong, PM2, PS4_Supporting, BP4.
19:11213452:G>*	*LDLR* Premature stop	p.Glu101Aspfs*105	1	The p.Glu101Aspfs*105 variant in LDLR has been reported in 2 individuals with hypercholesterolemia (Sozen 2004, Tosi 2007). It has also been identified in 0.001% (1/113754) of European chromosomes by gnomAD (http://gnomad.broadinstitute.org) and is reported in ClinVar (Variation ID: 251126). This variant is predicted to cause a frameshift, which alters the protein's amino acid sequence beginning at position 101 and leads to a premature termination codon 105 amino acids downstream. This alteration is then predicted to lead to a truncated or absent protein. Loss of function of the LDLR gene is an established disease mechanism in autosomal dominant hypercholesterolemia. In summary, this variant meets criteria to be classified as pathogenic for autosomal dominant hypercholesterolemia. ACMG/AMP Criteria applied: PVS1, PM2, PS4_Supporting.
19:11224296:G>A	*LDLR* Missense	p.Asp482Asn	5	The p.Asp482Asn variant in LDLR has been reported in at least 24 heterozygous individuals and 1 compound heterozyogus individual with hypercholesterolemia or early myocardial infarction and segregated with disease in 3 affected individuals from 2 families (Ward 1995, Webb 1996, Leren 2004, Graham 2005, Tichy 2012, Bertolini 2013, Lange 2014, Do 2015, Braenne 2015). It has also been identified in 0.009% (11/129018) of European chromosomes by gnomAD (http://gnomad.broadinstitute.org) and has been reported in ClinVar (Variation ID: 161284). Computational prediction tools and conservation analysis are consistent with pathogenicity. In vitro functional studies support an impact on protein function (Webb 1996). 3 variants at this position (p.Asp482Gly, p.Asp482His, p.Asp482Tyr) have been identified in patients with hypercholesterolemia, suggesting changes at this position may not be tolerated. In summary, this variant meets criteria to be classified as pathogenic for autosomal dominant hypercholesterolemia. ACMG/AMP Criteria applied: PS4, PM3, PP1, PP3, PS3_Supporting, PM5_Supporting.
19:11216146:C>G	*LDLR* Premature stop	p.Tyr188*	1	The p.Tyr188* variant in LDLR has been reported in 1 homozygous individual with severe hypercholesterolemia, 2 heterozygous individuals with hypercholesterolemia, and segregated with disease in 5 affected individuals from 1 family (Landsberger 1992. Humphries 2006). It was absent from large population studies, but is reported in ClinVar (Variation ID: 3727). This nonsense variant leads to a premature termination codon at position 188, which is predicted to lead to a truncated or absent protein. Loss of function of the LDLR gene is an established disease mechanism in autosomal dominant hypercholesterolemia. In vitro functional studies support an impact on protein function (Landsberger 1996). In summary, this variant meets criteria to be classified as pathogenic for autosomal dominant hypercholesterolemia. ACMG/AMP Criteria applied: PVS1, PM2, PM3, PP1_Moderate, PS3_Supporting, PS4_Supporting.
19:11223944:G>A	*LDLR* Splice site	c.1187-10G>A	1	The c.1187-10G>A variant in LDLR has been reported in 10 heterozygous individuals and 1 homozygous individual with hypercholesterolemia and segregated with disease in 10 affected individuals from 2 families (Wang 2011, Amsellem 2002, Punzalan 2005, Chmara 2010, Sun 2015, Liang 2016). It has also been identified in 0.005% (1/18364) of East Asian chromosomes by gnomAD (http://gnomad.broadinstitute.org) and is reported in ClinVar (Variation ID: 226349). This variant is located in the 3' splice region. Computational prediction tools and in vitro splicing assays are consistent with pathogenicity (Holla 2009). In vitro functional studies support an impact on protein function (Holla 2009, Romano 2011). In summary, this variant meets criteria to be classified as pathogenic for autosomal dominant hypercholesterolemia. ACMG/AMP Criteria applied: PP1_Strong, PM3, PS3_Moderate, PS4_Moderate.
19:11226820:G>A	*LDLR* Missense	p.Gly546Asp	1	The p.Gly546Asp variant in LDLR has been reported in 5 heterozygous individuals and 1 homozygous individual with hypercholesterolemia (Hobbs 1992, Fouchier 2001, Faiz 2013, Jannes 2015, Benedek 2018, Kim 2018). It was absent from large population studies, but is reported in ClinVar (Variation ID: 3697). Computational prediction tools and conservation analysis suggest that this variant may impact the protein, though this information is not predictive enough to determine pathogenicity. In vitro functional studies support an impact on protein function (Hobbs 1992). In summary, although additional studies are required to fully establish its clinical significance, this variant meets criteria to be classified as likely pathogenic for autosomal dominant hypercholesterolemia. ACMG/AMP Criteria applied: PM2, PM3, PP3, PS3_Supporting, PS4_Supporting.
19:11227549:C>T	*LDLR* Missense	p.Arg574Cys	1	The p.Arg574Cys variant in LDLR has been reported in 10 individuals with familial hypercholesterolemia (FH) and segregated with disease in 3 affected individuals from at least 2 families (Nauck 2001, Chmara 2010, Bertolini 2013, Thromaehlen 2015, Sharifi 2016, Guardamagna 2009, Do 2015). It has also been identified in 8/129194 European chromosomes by gnomAD (http://gnomad.broadinstitute.org) and has been reported in ClinVar (Variation ID: 183123). Computational prediction tools and conservation analysis suggest that this variant may impact the protein, though this information is not predictive enough to determine pathogenicity. In vitro functional studies provide some evidence that this variant does not impact LDLR uptake in cells

				(Thromaehlen 2015); however, these types of assays may not accurately represent biological function. Three additional variants involving this codon (p.Arg574) have been identified in individuals with FH (Stenson 2017). In summary, although additional studies are required to fully establish its clinical significance, this variant meets criteria to be classified as likely pathogenic for autosomal dominant FH. ACMG/AMP Criteria applied: PS4, PM2_Supporting, PP1, PP3.
19:11227612:C>T	*LDLR* Missense	p.Arg595Trp	1	The p.Arg595Trp variant in LDLR has been reported in at least 15 individuals with dominant or recessive familial hypercholesterolemia (Alonso 2016, Bañares 2017, Chiou 2011, Damgaard 2005, Descamps 2001, Fouchier 2005, Jannes 2015, Junyent 2010, Mozas 2004, Pek 2017, Pirillo 2017, Sánchez-Hernández 2016). It has also been identified in 2/129168 European chromosomes by gnomAD (http://gnomad.broadinstitute.org) and has also been reported in ClinVar (Variation ID: 161290). Computational prediction tools and conservation analysis suggest that this variant may impact the protein, though this information is not predictive enough to determine pathogenicity. Two other variants at this codon have been reported in indivdiuals with familial hypercholesterolemia (Stenson 2017). In summary, this variant meets criteria to be classified as pathogenic for autosomal dominant familial hypercholesterolemia. ACMG/AMP Criteria applied: PM3_VeryStrong, PS4, PM2, PP3.
19:11230767:G>A	*LDLR* Splice site	c.1846-1G>A	1	The c.1846-1G>A variant in LDLR has been reported in more than 16 individuals with familial hypercholesterolemia (FH) and segregated with disease in at least 13 affected individuals from 9 families (Jensen 1996, Nissen 1998, Bertolini 1999, Descamps 2001, Brusgaard 2006). It has also been identified in 1/8714 African chromosomes by gnomAD (http://gnomad.broadinstitute.org) and has been reported in ClinVar (Variation ID: 252079). This variant occurs within the canonical splice site (+/- 1,2) and sequencing of mRNA from patient cells has shown that this variant causes abnormal splicing, which is predicted to lead to an abnormal or absent protein (Jensen 1996, Bertolini 1999). Loss of function of the LDLR gene is an established disease mechanism in autosomal dominant FH. In summary, this variant meets criteria to be classified as pathogenic for autosomal dominant FH. ACMG/AMP criteria applied: PVS1, PS3, PS4, PP1_Strong, PM2.
19: 11110730:GC>TG	*LDLR* *Indel*	p.Cys340Leu	1	The p.Cys340Leu variant in LDLR has been reported in at least 3 individuals with familial hypercholesterolemia (FH): One in the heterozygous state (Marduel 2010) and two in the homozygous state (where at least one of these individuals was from a consanguineous family) and segregated with disease in more than 6 heterozygous affected family members from 2 families (Ahmed 2013, ClinVar; submission accession: SCV000503280.1). It has also been identified in 0.007% (2/30614) of South Asian chromosomes by gnomAD (http://gnomad.broadinstitute.org). Three additional variants at this codon have been reported in individuals with FH (p.Cys340Phe, p.Cys340Trp and p.Cys340Tyr), suggesting variation at this position may not be tolerated (Stenson 2017). Computational prediction tools and conservation analysis suggest that the p.Cys340Leu variant may impact the protein. In summary, although additional studies are required to fully establish its clinical significance, this variant meets criteria to be classified as likely pathogenic for autosomal dominant FH. ACMG/AMP Criteria applied: PM2; PP1_Moderate; PP3; PM3_Supporting
1:55523127:G>T	*PCSK9* Missense	p.Asp374Tyr	1	The p.Asp374Tyr variant in PCSK9 has been reported in at least 12 individuals with hypercholesterolemia and segregated with disease in at least 9 affected individuals from 1 family (Timms 2004, Humphries 2009, Kaya 2017). It was absent from large population studies, but has been reported in ClinVar (Variation ID: 2875). Computational prediction tools and conservation analysis are consistent with pathogenicity. In vitro functional studies support an impact on protein function (Benjannet 2004, Bottomley 2009, Al-Mashhadi 2013). In summary, this variant meets criteria to be classified as pathogenic for autosomal dominant hypercholesterolemia. ACMG/AMP Criteria applied: PP1_Strong, PM2, PS4_Moderate, PP3, PS3_Supporting.

Supplementary Table 2: Effect of the polygenic score among carriers and noncarriers of monogenic risk variants in the case-control studies

Coronary artery disease case-control study (N=12,852)					
	N total	N cases	N controls	OR per standard deviation (95% CI)	p-value
FH carriers	56	43	13	2.31 (1.16-4.57)	0.016
Noncarriers	12796	6389	6407	1.74 (1.68-1.81)	$6x10^{-75}$
**Breast cancer case-control study (N=19,264)**					
	N total	N cases	N controls	OR per standard deviation (95% CI)	p-value
HBOC carriers	845	174	671	1.44 (1.19-1.74)	$1.2x10^{-4}$
Noncarriers	18419	1746	16673	1.57 (1.49-1.65)	$< 2x10^{-16}$

The odds ratio (OR) per standard deviation was assessed in a logistic regression model with age, sex, and the first four principal components of ancestry as covariates in the coronary artery disease case-control study, and age and the first four principal components of ancestry as covariates in the breast cancer case-control study. p-values were estimated by the Wald Test. Statistical significance was set at $p < .05$, and 2-sided p values were used. Abbreviations: FH familial hypercholesterolemia, HBOC hereditary breast and ovarian cancer.

Supplementary Table 3: Odds ratio of disease comparing carriers of monogenic risk variants with high vs. low polygenic score

Familial hypercholesterolemia variant carriers in the coronary artery disease case-control study (N=56)									
Comparison group	N total	N cases	N controls	Reference group	N total	N cases	N controls	OR (95% CI)	p-value
Carriers with high polygenic risk	25	23	2	Carriers with low polygenic risk	11	6	5	9.69 (1.48-63.38)	0.018
Hereditary breast and ovarian cancer variant carriers in the breast cancer case-control study (N=845)									
Comparison group	N total	N cases	N controls	Reference group	N total	N cases	N controls	OR (95% CI)	p-value
Carriers with high polygenic risk	173	48	125	Carriers with low polygenic risk	185	32	153	3.04 (1.61-5.73)	0.0004

Carriers with high polygenic risk (defined as the highest quintile of the polygenic score distribution) were compared to carriers with low polygenic score (defined as the lowest quintile of the polygenic score distribution). The odds ratio (OR) was assessed in a logistic regression model with age, sex, and the first four principal components of ancestry as covariates in the coronary artery disease case-control study, and age and the first four principal components of ancestry as covariates in the breast cancer case-control study. p-values were estimated by the Wald Test. Statistical significance was set at $p < .05$, and 2-sided p-values were used.

Supplementary Table 4: Effect of non-cholesterol polygenic background on risk of coronary artery disease in familial hypercholesterolemia

	OR per standard deviation (95% CI)	p-value
Coronary artery disease polygenic score		
FH carriers	2.31 (1.16-4.57)	0.016
Noncarriers	1.74 (1.68-1.81)	$6x10^{-75}$
Coronary artery disease polygenic score residualized to LDL cholesterol polygenic score		
FH carriers	2.37 (1.13-4.95)	0.02
Noncarriers	1.68 (1.62-1.74)	$1.9x10^{-155}$
Coronary artery disease polygenic score with variants within 2 megabases of familial hypercholesterolemia related genes removed		
FH carriers	2.77 (1.27-6.04)	0.01
Noncarriers	1.73 (1.67-1.80)	$4.5x10^{-172}$

For each of the three scores shown, in carriers and in noncarriers separately, the odds ratio (OR) was assessed in a logistic regression model with age, sex, and the first four principal components of ancestry as covariates. p-values were estimated by the Wald Test. Statistical significance was set at $p < .05$, and 2-sided p-values were used. Abbreviations: LDL low density lipoprotein

Supplementary Table 5: Evidence in support of pathogenic or likely pathogenic assertions for three genomic conditions in 48,812 UK Biobank participants

Variant	Gene (Variant Type)	Amino acid or cDNA change	Number of carriers	Evidence in Support of Pathogenicity Assessment
Familial hypercholesterolemia variants				
1:55505604:G>A	*PCSK9* Missense	p.Glu32Lys	1	The p.Glu32Lys variant in PCSK9 has been reported in >40 Japanese and Korean individuals with hypercholesterolemia, including 2 homozygous individuals and 9 double heterozygotes who had an additional pathogenic variant in LDLR (Miyake 2008, Mabuchi 2011, Noguchi 2010, Mabuchi 2014, Han 2015, Hopkins 2015, ClinVar; variation ID: 297692). Homozygotes and double heterozygotes had more severe disease on average than heterozygotes, and heterozygotes for this variant had milder disease than heterozygotes for other variants associated to familial hypercholesterolemia (FH; Mabuchi 2014, Hopkins 2015). Additionally, this variant segregated with disease in >20 affected relatives from >5 families (Noguchi 2010, Mabuchi 2014). In vitro functional studies provide some evidence that the p.Glu32Lys variant may impact protein function (Noguchi 2010). This variant has also been identified in 4/12338 East Asian chromosomes by gnomAD (http://gnomad.broadinstitute.org/). This frequency is low enough to be consistent with the frequency of FH in the general population. In summary, the p.Glu32Lys variant meets criteria to be classified as pathogenic for autosomal dominant familial hypercholesterolemia based upon presence in multiple affected individuals and segregation with disease. The ACMG/AMP Criteria applied: PS4, PP1_Strong, PS3_Supporting.
1:55523127:G>T	*PCSK9* Missense	p.Asp374Tyr	1	The p.Asp374Tyr variant in PCSK9 has been reported in at least 12 individuals with hypercholesterolemia and segregated with disease in at least 9 affected individuals from 1 family (Timms 2004, Humphries 2009, Kaya 2017). It was absent from large population studies, but has been reported in ClinVar (Variation ID: 2875). Computational prediction tools and conservation analysis are consistent with pathogenicity. In vitro functional studies support an impact on protein function (Benjannet 2004, Bottomley 2009, Al-Mashhadi 2013). In summary, this variant meets criteria to be classified as pathogenic for autosomal dominant hypercholesterolemia. ACMG/AMP Criteria applied: PP1_Strong, PM2, PS4_Moderate, PP3, PS3_Supporting.
1:55524303:C>T	*PCSK9* Missense	p.Arg496Trp	5	The p.Arg496Trp variant in PCSK9 has been reported in the heterozygous state in >25 individuals with hypercholesterolemia or primary dislipidemia, the majority of whom were from Turkey or the Netherlands, and segregated with disease in 1 affected individual (Bertolini 2013, Hopkins 2015, Ohta 2016, Kaya 2017, Martin-Campos 2018, Eroglu 2018, Invitae pers. comm., GeneDx pers. comm.). In addition, it was identifed in an individual with a severe presentation who also carried a pathogenic variant in LDLR. His affected mother also carried the PCSK9 variant (Pisciotta 2006). It has been seen in the homozygous state in 2 individuals with hypercholesterolemia (Kaya 2017, Eroglu 2018, Invitae pers. comm.). A case control study of Turkish individuals showed that individuals carrything this variant were statistically more likely to be affected with primary dislipidemia compared to controls and had a 12.8-fold higher triglyceride levels compared to controls (Eroglu 2018). This variant has also been identified in 0.026% (8/30508) of South Asian chromosomes and 0.003% (4/126638) of European chromosomes by gnomAD (http://gnomad.broadinstitute.org) and has been reported in ClinVar (Variation ID 201129). An in vitro functional studies showed a modest gain of function impact (Fasano 2009) and 2 additional studies did not demonstrate a significant functional change (Pisciotta 2006, Ly 2014); however, these types of assays may not accurately represent biological function.

				Computational prediction tools and conservation analyses do not provide strong support for or against an impact to the protein and 1 mammal carries Tryptophan (Trp) at this posistion with high nearby conservation. In summary, although additional studies are required to fully establish its clinical significance particularly because the allele frequency of this variant in the South Asian population of gnomAD is relatively high and functional studies are unclear, this variant meets criteria to be classified as likely pathogenic for autosomal dominant familial hypercholesterolemia. ACMG/AMP Criteria applied: PS4, PM3.
2:21229160:C>T	*APOB* Missense	p.Arg3527Gln	36	The p.Arg3527Gln variant in APOB is a well-established pathogenic variant that is mainly found in individuals of European descent. It has been previously reported in >500 individuals with familial hypercholesterolemia (FH) and segregated with disease in >50 affected relatives (Soria 1989, März 1993, Leren 1997, Ludwig 1990, Bednarska-Makaruk 2001, Horvath 2001, Kalina 2001). It has also been reported by other clinical laboratories in ClinVar (Variation ID 17890) and has been identified in 53/126056 of European chromosomes, including 1 homozygote, by the Genome Aggregation Database (gnomAD, http://gnomad.broadinstitute.org/; dbSNP rs5742904). This frequency is low enough to be consistent with the frequency of FH in the general population. In summary, this variant meets criteria to be classified as pathogenic for autosomal dominant familial hypercholesterolemia based upon presence in multiple affected individuals and segregation studies. Please note that pathogenic variants in APOB can have reduced penetrance and a less severe phenotype than disease-causing LDLR or PCSK9 variants (Youngblom and Knowles, GeneReviews). ACMG/AMP Criteria applied: PS4_Strong; PP1_Strong.
2:21229161:G>A	*APOB* Missense	p.Arg3527Trp	3	The p.Arg3527Trp variant (also referred to in the literature as p.Arg3500Trp) in APOB has been reported in at least 33 individuals with familial hypercholesterolemia, the majority of whom are of East Asian ancestry, and segregated with disease in at least 15 affected relatives from 4 families (Gaffney 1995, Choong 1997, Tai 1998, Fisher 1999, Tai 2001, Yang 2007, Hollandt 2012, Chiou 2010, Chiou 2011, Chiou 2012, Bertolini 2013). This variant has been reported in ClinVar (Variation ID 40223) and has also been identified in 22/18848 East Asian chromosomes by the Genome Aggregation Database (gnomAD, http://gnomad.broadinstitute.org; dbSNP rs144467873). This frequency is low enough to be consistent with the frequency of familial hypercholesterolemia (FH) in the general population. In vitro functional studies provide some evidence that the p.Arg3527Trp variant may impact protein function (Gaffney 1995, Fisher 1999, Tai 2001). Additionally, another variant at this position, p.Arg3527Gln, is a well-established pathogenic variant for FH. In summary, this variant meets criteria to be classified as pathogenic for FH in an autosomal dominant manner based upon segregation studies, increased prevalence in affected individuals, and pathogenicity of other variants at this position. Please note that pathogenic variants in APOB can have reduced penetrance and a less severe phenotype than disease-causing LDLR or PCSK9 variants (Youngblom and Knowles, GeneReviews).
19:11210949:A>*	*LDLR* Frameshift	p.Ile40SerfsX166	1	The p.Ile40SerfsX166 variant in LDLR has been identified in at least 3 individuals with hypercholesterolemia (Heath 1999, Martin 2016). It was absent from large population studies. This variant is predicted to cause a frameshift, which alters the protein's amino acid sequence beginning at position 40 and leads to a premature termination codon 166 amino acids downstream. This alteration is then predicted to lead to a truncated or absent protein. Loss of function of the LDLR gene is an established disease mechanism in autosomal dominant familial hypercholesterolemia. In summary, this variant meets criteria to be classified as pathogenic for autosomal dominant familial hypercholesterolemia. ACMG/AMP Criteria applied: PVS1, PM2, PS4_Supporting.
19:11213360:G>*	*LDLR* Frameshift	p.Asp72ThrfsX134	1	The p.Asp72ThrfsX134 variant in LDLR has been identified in at least 3 individuals with hypercholesterolemia and segregated with disease in >15 individuals from a large family (Ward 1996, Martin 2016, Defesche 2017). It has also been identified in 1/113766 European chromosomes by gnomAD (https://gnomad.broadinstitute.org). This variant is predicted to cause a frameshift, which alters the protein's amino acid sequence beginning at position 72 and leads to a premature termination codon 134 amino acids downstream. This alteration is then predicted to lead to a truncated or absent protein. Loss of function of the LDLR gene is an established disease mechanism

				in autosomal dominant familial hypercholesterolemia. In summary, this variant meets criteria to be classified as pathogenic for autosomal dominant familial hypercholesterolemia. ACMG/AMP Criteria applied: PVS1, PP1_Strong, PM2, PS4_Supporting.
19:11213390:C>T	*LDLR* Missense	p.Arg81Cys	1	The p.Arg81Cys variant in LDLR (also described as p.Arg81Cys in the literature) has been reported in >18 individuals with familial hypercholesterolemia (FH; Nissen 1998, Loubser 1999, Fouchier 2001, Bourbon 2008, Alonso 2009, Huijgen 2010, Huijgen 2011, Bertolini 2013), though not all individuals had extremely elevated LDL-cholesterol levels (Huijen 2011). It has been suggested that the p.Arg81Cys variant results in an LDLR protein that does not function as effectively as that produced by the wild-type allele, thus resulting in only modest LDL-cholesterol elevations (Huijgen 2010). This variant has also been reported by other clinical laboratories in ClinVar (Variation ID: 183083) and has been identified in 2/111718 European chromosomes by the Genome Aggregation Database (gnomAD, http://gnomad.broadinstitute.org; dbSNP rs730882078). This frequency is low enough to be consistent with the frequency of FH in the general population. Computational prediction tools and conservation analysis do not provide strong support for or against an impact to the protein. In summary, although additional studies are required to fully establish its clinical significance, the p.Arg81Cys variant is likely pathogenic. The ACMG/AMP Criteria applied (Richards 2015): PS4_Strong, PM2.
19:11213408:T>G	*LDLR* Missense	p.Trp87Gly	2	The p.Trp87Gly variant in LDLR is a well-established pathogenic variant for familial hypercholesterolemia (Leitersdorf 1990, Jensen 1996, Vohl 1997, Tybjaerg-Hansen 2005, Futema 2013), and is a known founder mutation in the French Canadian population where it has been reported in >400 individuals with FH, including >15 homozygous individuals (Vohl 1997). It has also been identified in 5/66740 European chromosomes by the Exome Aggregation Consortium (ExAC, http://exac.broadinstitute.org; dbSNP rs121908025); however, this frequency is low enough to be consistent with the frequency of FH in the general population. Additionally, in vitro functional studies provide some evidence that the p.Trp87Gly variant may impact protein function (Leitersdorf 1990). In summary, this variant meets our criteria to be classified as pathogenic for FH in an autosomal dominant manner based upon its identification in a large number of affected individuals and low frequency in controls.
19:11213415:G>A	*LDLR* Missense	p.Cys89Tyr	2	The p.Cys89Tyr variant in LDLR has been reported in the heterozygous state in 7 individuals with hypercholesterolemia and segregated with disease in 1 affected individual (Day 1997, Graham 1999, Fouchier 2005, Humphries 2006, Tosi 2010, Wald 2016). It was also identified in the compound heterozygous state with a deletion of exons 16 and 17 in a child with a severe presentation. His father carried the p.Cys89Tyr variant and also had hypercholesterolemia (Tosi 2007). It was absent from large population studies. Computational prediction tools and conservation analyses suggest that this variant may impact the protein, though this information is not predictive enough to determine pathogenicity. Additional variants involving this codon (p.Cys89Trp, p.Cys89Arg, and p.Cys89Gly) have been identified in individuals with hypercholesterolemia. In summary, although additional studies are required to fully establish its clinical significance, this variant meets criteria to be classified as likely pathogenic for autosomal dominant familial hypercholesterolemia. ACMG/AMP Criteria applied: PM2, PM3, PS4_Moderate, PP3.
19:11213450:G>A	*LDLR* Missense	p.Glu101Lys	6	The p.Glu101Lys variant in LDLR has been reported in the heterozygous state in >30 individuals with familial hypercholesterolemia (FH), in the compound heterozygous state in 1 individual with homozygous FH (Loux 1992, Webb 1992, García-García 2001, Mozas 2004, Humphries 2006, Miyakem2009, Taylor 2010, Futema 2013, Do 2015) and segregated with disease in four affected relatives from two families (Webb 1992, Loux 1992). This variant has also been reported by other clinical laboratories in ClinVar (Variation ID# 161266). In vitro functional studies provide some evidence that the p.Glu101Lys variant may impact protein function (Webb 1992). This variant has also been identified in 3/111700 European chromosomes by the genome Aggregation Database (gnomAD, http://gnomad.broadinstitute.org). This frequency is low enough to be consistent with the frequency of FH in the general population. Computational prediction tools and conservation analysis suggests that

				the p.Glu101Lys variant may impact the protein, though this information is not predictive enough to determine pathogenicity. In summary, this variant meets criteria to be classified as pathogenic for familial hypercholesterolemia in an autosomal dominant manner based upon presence in multiple affected individuals, segregation studies, low frequency in the general population and functional evidence. The ACMG/AMP Criteria applied: PS4, PM2, PS3_Moderate, PP1, PP3.
19:11213462:CG>*	*LDLR* splice site	c.313_313+1delCG	1	The c.313_313+1delCG variant in LDLR has been reported in 2 individuals with hypercholesterolemia (Hobbs 1992, Hooper 2012). It was absent from large population studies. This variant occurs within the canonical splice site (+/- 1,2) and is predicted to cause altered splicing leading to an abnormal or absent protein. An in vitro study supports an impact on splicing (Hobbs 1992). Loss of function of the LDLR gene is an established disease mechanism in autosomal dominant familial hypercholesterolemia. In summary, this variant meets criteria to be classified as pathogenic for autosomal dominant familial hypercholesterolemia. ACMG/AMP Criteria applied: PVS1, PM2, PS4_Supporting.
19:11213463:G>C	*LDLR* splice site	c.313+1G>C	1	The c.313+1G>C variant in LDLR has been identified in >80 Spanish individuals with familial hypercholesterolemia (FH; Tejedor 2011). It has also been identified in 1/113710 European chromosomes by gnomAD (https://gnomad.broadinstitute.org). This variant occurs in the invariant region (+/- 1,2) of the splice consensus sequence and is predicted to cause altered splicing leading to an abnormal or absent protein. Heterozygous loss of LDLR function is an established disease mechanism in FH. In summary, this variant meets our criteria to be classified as pathogenic for FH in an autosomal dominant manner. PVS1_Strong, PS4, PM2.
19:11213463:G>A	*LDLR* splice site	c.313+1G>A	1	The c.313+1G>A variant in LDLR has been reported in >140 individuals with familial hypercholesterolemia (FH) and segregated with disease in at least 5 affected relatives from 2 families (Leren 1994, Sun 1995, Lombardi 2000, Hooper 2012). This variant has also been identified in 7/111670 European chromosomes by gnomAD (http://gnomad.broadinstitute.org). This frequency is low enough to be consistent with the frequency of FH in the general population. The c.313+1G>A variant occurs in the invariant region (+/- 1,2) of the splice consensus sequence and is predicted to cause altered splicing that would preserve the protein reading frame, leading to an abnormal protein. Furthermore, in vitro functional studies provide some evidence that this variant may impact protein function (Sun 1995, Cameron 2009). In summary, this variant meets criteria to be classified as pathogenic for FH in an autosomal dominant manner based upon presence in multiple affected individuals, segregation studies, low frequency in the general population, and impact to the protein. The ACMG/AMP Criteria applied: PS4, PM2, PP1_Moderate, PVS1_Moderate.
19:11215919:G>A	*LDLR* Missense	p.Glu113Lys	4	The p.Glu113Lys variant in LDLR has been reported in 2 individuals with hypercholesterolemia, 1 individual with probable hypercholesterolemia, and segregated with disease in 7 affected individuals from 1 family (Wu 2000, Fouchier 2005, Taylor 2007). It has also been identified in 0.002% (3/128566) of European chromosomes by gnomAD (http://gnomad.broadinstitute.org) and is reported in ClinVar (Variation ID: 237872). Computational prediction tools and conservation analysis suggest that this variant may not impact the protein, though this information is not predictive enough to rule out pathogenicity. In summary, although additional studies are required to fully establish its clinical significance, this variant meets criteria to be classified as likely pathogenic for autosomal dominant hypercholesterolemia. ACMG/AMP Criteria applied: PP1_Strong, PM2, PS4_Supporting, BP4.
19:11216083:C>A	*LDLR* Nonsense	p.Cys167X	1	The p.Cys167X variant in LDLR (also described as p.Cys146X in the literature) has been reported in the heterozygous state in >10 individuals with familial hypercholesterolemia (FH), segregated with disease in one affected relative from one family (Lombardi 1995, Heath 1999, Fouchier 2001, Bodamer 2002, van der Graaf 2011, Tichy 2012, Sharifi 2016) and was absent from large population studies. Additionally, this variant has been reported by other clinical laboratories in ClinVar (Variation ID: 200918). This nonsense variant leads to a premature termination codon at position 167, which is predicted to lead to a truncated or absent protein. Heterozygous loss of function of the LDLR gene is an established disease mechanism in FH. In summary, this variant meets our criteria to be classified as pathogenic for familial hypercholesterolemia in an autosomal dominant manner based upon

				predicted impact to the protein, presence in multiple affected individuals and absence in the general population. ACMG/AMP Criteria applied (Richards 2015): PVS1, PS4_Moderate, PM2.
19:11216084:G>A	*LDLR* Missense	p.Asp168Asn	3	The p.Asp168Asn variant in LDLR has been reported in at least 8 individuals with familial hypercholesterolemia (FH) and in 2 individuals with early-onset myocardial infarction (Day et al. 1997, Lee et al. 1998, Punzalan et al. 2005, Do et al., 2015, ClinVar: Variation ID 183136). In vitro functional studies provide some evidence that the p.Asp168Asn variant may cause a decrease in LDL uptake and binding (Etxebarria 2015). This variant has also been identified in 2/111620 European chromosomes by the Genome Aggregation Database (GnomAD, http://gnomad.broadinstitute.org; dbSNP rs200727689). Computational prediction tools and conservation analysis suggest that the p.Asp168Asn variant may impact the protein. Additionally, other variants at this position have been reported in association with FH in the Human Gene Mutation Database (HGMD; Stenson et al. 2017). In summary, although additional studies are required to fully establish its clinical significance, the p.Asp168Asn variant is likely pathogenic. ACMG/AMP Criteria applied (Richards 2015): PS4_Moderate, PS3_Supporting, PM2, PP3.
19:11216233:TGG>*	*LDLR* Deletion	p.Gly219del	2	The p.Gly219del variant in LDLR (also known as FH Lithuania and G197del) has been reported in >75 families with hypercholesterolemia (Hobbs 1990, Meiner 1991, Gudnason 1993, Gorski 1998, Mandelshtam 1998, Heath 1999, Durst 2001, Kuhrova 2002, Taylor 2007, Junyent 2008, Chmara 2010, Tichy 2012, Hooper 2012, Sharifi 2016, Durst 2017, Smyth 2018). It is considered to be a founder mutation in the Ashkenazi Jewish population (Meiner 1991, Durst 2001). This variant has also been identified in 0.05% (5/10062) of Ashkenazi Jewish chromosomes by gnomAD (http://gnomad.broadinstitute.org) and has been reported in ClinVar (Variation ID 226329). This variant is a deletion of 1 amino acid at position 219 and is not predicted to alter the protein reading-frame. In vitro functional studies support an impact on protein function (Hobbs 1990). This variant meets the following ACMG/AMP Criteria: PS4, PM2, PM4_Supporting, PS3_Supporting. Based on these criteria, the variant would be classified as likely pathogenic but its recognized role as a founder mutation lends additional weight. In summary, the p.Gly219del is classified as pathogenic for autosomal dominant hypercholesterolemia.
19:11216244:A>G	*LDLR* Missense	p.Asp221Gly	3	The p.Asp221Gly variant in LDLR has been reported in >75 individuals with familial hypercholesterolemia (FH), including in 4 homozygotes who presented with more severe disease (Hobbs 1992, Chmara 2010, Bertolini 2013). However, not all individuals carrying this variant presented with high cholesterol levels (Bertolini 2013, Thormaehlen 2015). This variant has also been reported by other clinical laboratories in ClinVar (Variation ID 183092) and has been identified in 13/111132 European chromosomes by the Genome Aggregation Database (gnomAD, http://gnomad.broadinstitute.org; dbSNP rs373822756). Please note that this frequency is low enough to be consistent with the frequency of FH in the general population. In vitro functional studies provide some evidence that the p.Asp221Gly variant may impact protein function (Thormaehlen 2015). In summary, this variant meets criteria to be classified as pathogenic for familial hypercholesterolemia in an autosomal dominant manner based upon presence in affected individuals, low frequency in the general population, computational and functional evidence. ACMG/AMP Criteria applied: PS4, PM3_Strong, PP3, PS3_Supporting.
19:11216262:AC>*	*LDLR* Frameshift	p.Asp227GlyfsX12	2	The p.Asp227fs variant in LDLR has been reported in at least 9 individuals with hypercholesterolemia (Gudnason 1993, Graham 1999, Bunn 2002, Dedoussis 2004, Martin 2016) and has also been reported in ClinVar (Variation ID #3731). This variant has also been identified in 1/110684 European chromosomes by the Genome Aggregation Database (gnomAD, http://gnomad.broadinstitute.org; dbSNP rs387906305). This variant is predicted to cause a frameshift, which alters the protein’s amino acid sequence beginning at position 227 and leads to a premature termination codon 12 amino acids downstream. This alteration is then predicted to lead to a truncated or absent protein. Heterozygous loss of function of the LDLR gene is an established disease mechanism in familial hypercholesterolemia (FH). In summary, this variant meets criteria to be classified as pathogenic for FH in an autosomal dominant manner based upon the predicted impact to the protein, presence in

				multiple affected individuals and low frequency in controls. ACMG/AMP Criteria applied: PVS1, PM2, PS4_Moderate.
19:11216263:C>G	*LDLR* Missense	p.Asp227Glu	1	The p.Asp227Glu variant in LDLR (also known as the FH Afrikaner 1 allele) is a founder variant in the Afrikan population and is thought to account for 65 - 75% of familial hypercholesterolemia in Afrikans (Leitersdorf 1989, Kotze 1990, Kotze 1994). This variant has also been identified in at least 7 Caucasian individuals with familial hypercholesterolemia (Gudnason 1993, Callis 1998, Fouchier 2001, Bertolini 2013, Sharifi 2016) and has been reported in ClinVar (Variation ID 3690). Additionally, in vitro functional studies provide some evidence that the p.Asp227Glu variant may impair receptor activity (Fourie 1988). This variant has been identified in 1/33548 Latino chromosomes by the Genome Aggregation Database (gnomAD; http://gnomad.broadinstitute.org; dbSNP rs121908028). This frequency is low enough to be consistent with the frequency of familial hypercholesterolemia (FH) in the general population. In summary, this variant meets criteria to be classified as pathogenic for FH in an autosomal dominant manner based upon low frequency in controls, functional evidence, and presence in multiple affected individuals. ACMG/AMP Criteria applied: PS4, PP1_Strong, PM2, PP3, PS3_Supporting.
19:11216264:G>T	*LDLR* Nonsense	p.Glu228X	1	The p.Glu228X variant in LDLR (also described as p.Glu207X in the literature) has been reported in 23 individuals with familial hypercholesterolemia (FH; Hobbs 1992, Nauck 2001, Bodamer 2002, Kim 2004, Taylor 2007, Hola 2009, Vandrovcova 2013, Han 2015, Du 2016, Abul-Husn 2016) and segregated with disease in 6 affected relatives from 3 families (Bodamer 2002, Kim 2004). This variant has also been reported by other clinical laboratories in ClinVar (Variation ID 226333). In vitro functional studies provide some evidence that the p.Glu228X variant may impact protein function (Hobbs 1992, Holla 2009). This variant has also been identified in 1/23818 African and 2/125438 European chromosomes by the Genome Aggregation Database (gnomAD, http://gnomad.broadinstitute.org; dbSNP rs12190829). This frequency is low enough to be consistent with the frequency of FH in the general population. This nonsense variant leads to a premature termination codon at position 228, which is predicted to lead to a truncated or absent protein. Heterozygous loss of function of the LDLR gene is an established disease mechanism in individuals with FH. In summary, this variant meets criteria to be classified as pathogenic for FH in an autosomal dominant manner based upon presence in multiple affected individuals, segregation studies, low frequency in the general population, predicted impact on the protein, and functional evidence. The ACMG/AMP criteria applied (Richards 2015): PVS1; PM2; PS4; PP1_Moderate; PS3_Supporting.
19:11217264:G>A	*LDLR* Missense	p.Glu240Lys	2	The p.Glu240Lys variant in LDLR (also described as p.Gly219Lys in the literature) has been reported in 6 individuals with familial hypercholesterolemia (FH) and segregated with disease in 3 affected relatives from two families (Fouchier 2005, Bertolini 2013, Hobbs 1992, Mollaki 2014, Norsworthy 2014). However, this variant has also been reported in one individual with normal cholesterol levels (Abul-Husn 2016), suggesting reduced penetrance. In vitro functional studies provide some evidence that the p.Glu240Lys variant may impact protein folding and transport of the mature protein from the ER to the Golgi (Hobbs 1992, North 2000, North 2001). This variant has also been reported by other clinical laboratories in ClinVar (Variation ID 200920) and has been identified in 7/126728 European chromosomes by the Genome Aggregation Database (gnomAD, http://gnomad.broadinstitute.org/; dbSNP 7685633000). This frequency is consistent with the frequency of FH in the general population. Computational prediction tools and conservation analysis do not provide strong support for or against an impact to the protein. In summary, although additional studies are required to fully establish its clinical significance, the p.Glu240Lys variant is likely pathogenic. The ACMG/AMP Criteria applied: PM2; PS3_Supporting; PS4_Moderate; PP1.
19:11217344:T>A	*LDLR* Missense	p.Asp266Glu	1	The p.Asp266Glu variant has been reported in over 100 individuals with familial hypercholesterolemia (FH; Bertolini 2013, Brænne 2015, Brusgaard 2006, Chmara 2010, Do 2015, Fouchier 2001, Hobbs 1992, Schmidt 2000, Sharifi 2016, Tichý 2012, Weiss 2000). Some publications refer to this variant as p.Asp245Glu (or p.D266E). This variant has also been reported by other clinical laboratories in ClinVar (Variation ID: 161287) and has been identified in 10/126728 European chromosomes by the

				Genome Aggregation Database (GnomAD, http://gnomad.broadinstitute.org; dbSNP rs139043155). In vitro functional studies provide some evidence that the p.Asp266Glu variant may impact protein function, resulting in 15-30% LDL receptor activity (Hobbs 1992). Computational prediction tools and conservation analysis suggest that the p.Asp266Glu variant may impact the protein. Another likely pathogenic missense change at the same position (p.Asp266Tyr) has been reported in association to FH (reported as p.Asp245Tyr, Weiss 2000). In summary, this variant meets criteria to be classified as pathogenic for familial hypercholesterolemia in an autosomal dominant manner based upon presence in a large number of affected individuals, low frequency in controls, presence of another pathogenic missense change at the same amino acid position, functional evidence and computational evidence. ACMG/AMP Criteria applied: PS4, PM2, PM5, PP3, PS3_supporting (Richards 2015).
19:11218112:G>A	*LDLR* Missense	p.Glu288Lys	1	The p.Glu288Lys variant in LDLR has been reported in >25 individuals with familial hypercholesterolemia and segregated with disease in 1 affected relative (Ebhardt 1999, Fouchier 2001, Bunn 2002, Bourbon 2008, Alonso 2009, Etxebarria 2012, Bertolini 2013, ArulJothi 2016). Additionally, this variant has been identified in 6/30782 South Asian chromosomes by the Genome Aggregation Database (gnomAD, http://gnomad.broadinstitute.org/; dbSNP rs368657165) and is present in ClinVar (Variation ID: 161268). Please note that for diseases with clinical variability, reduced penetrance, or recessive inheritance, pathogenic variants may be present at a low frequency in the general population. In vitro functional studies provide some evidence that the p.Glu288Lys variant may impact protein function (Etxebarria 2012). However, these types of assays may not accurately represent biological function. Computational prediction tools and conservation analysis do not provide strong support for or against an impact to the protein. In summary, although additional studies are required to fully establish its clinical significance, the p.Glu288Lys variant is likely pathogenic. The ACMG/AMP Criteria applied: PS4, PM2_Supporting, PS3_Supporting
19:11218162:C>G	*LDLR* Missense	p.Asp304Glu	1	The p.Asp304Glu variant in LDLR has been reported in at least 3 individuals with familial hypercholesterolemia, two of which were compound heterozygotes (FH; Hobbs 1992, Tosi 2007, Webb 1996). It has also been reported by other clinical laboratories in ClinVar (Variation ID 226336) and is absent from large population studies. In vitro functional studies have shown that cultured fibroblasts from compound heterozygous carriers of the p.Asp304Glu variant have reduced LDLR activity (2-5% with c.2309-?_*2514+?del and 25-30% with p.Asp96Gly; Hobbs 1992, Webb 1996). In addition, other disease-causing variants (p.Asp304Asn, p.Asp304Tyr) at this position have been reported in individuals with FH (Hobbs 1992, Do 2015, Loux 1992, Thormaehlen 2015, Tichy 2012, Vohnout 2016), suggesting changes at this position are not tolerated. In summary, although additional studies are required to fully establish its clinical significance, the p.Asp304Glu variant is likely pathogenic. ACMG/AMP Criteria applied: PM2, PM5, PS3_Moderate, PS4_Supporting (Richards 2015).
19:11221390:G>A	*LDLR* Missense	p.Gly335Ser	2	The p.Gly335Ser variant in LDLR (also described as p.Glu314Ser in the literature) has been reported in 7 heterozygous individuals with hypercholesterolemia, 1 double heterozygous with hypercholesterolemia (with a pathogenic PCSK9 variant) individual, and 1 compound heterozygous individual with homozygous hypercholesterolemia (HoHF; Bertolini 2013, Abul-Husn 2016, Hobbs 1992, Laurie 2004, Rabacchi 2016, Retterer 2015, Wang 2001) as well as 2 individuals with a myocardial infarction (Do 2015, Thomaehlen 2015). This variant segregated in the compound heterozygous state in 3 affected relatives from 1 family, one of which displayed features of HoHF (Rabacchi 2016). Functional studies provide conflicting evidence on the impact of this variant on the protein (Hobbs 1992, Thormaehlen 2015). This variant has also been reported in ClinVar (Variation ID 183105) and identified in 7/126504 European chromosomes by the Genome Aggregation Database (gnomAD, http://gnomad.broadinstitute.org). Computational prediction tools and conservation analysis suggest that the p.Gly335Ser variant may impact the protein, though this information is not predictive enough to determine pathogenicity. In summary, although additional studies are required to fully establish its clinical significance, this variant meets criteria to be classified as likely pathogenic for

				familial hypercholesterolemia (FH) in an autosomal dominant manner. ACMG/AMP Criteria applied: PS4_Moderate, PM3, PP1, PP3, PM2_Supporting.
19:11221435:C>T	*LDLR* Nonsense	p.Arg350X	3	The p.Arg350X variant in LDLR (also described as p.Arg329X in the literature) has been reported in >15 individuals with hypercholesterolemia and segregated with disease in >15 affected relatives from 8 families (Day 1997, Humphries 2006, Kubalska 2008, Dušková 2011, van der Graaf 2011, Tichý 2012, Huijgen 2012, Radovica-Spalvina 2015, Do 2015, Fan 2015). This variant has also been identified in 1/111552 European chromosomes by the Genome Aggregation Database (gnomAD, http://gnomad.broadinstitute.org; dbSNP rs769737896) and in ClinVar (Variation ID: 226342). This nonsense variant leads to a premature termination codon at position 350, which is predicted to lead to a truncated or absent protein. Heterozygous loss of function of the LDLR gene is an established disease mechanism in individuals with familial hypercholesterolemia (FH). In summary, this variant meets criteria to be classified as pathogenic for FH in an autosomal dominant manner. The ACMG/AMP Criteria applied: PVS1; PS4; PP1_Strong; PM2.
19:11222190:A>G	*LDLR* Missense	p.Asp354Gly	1	The p.Asp354Gly variant in LDLR (also described as p.Asp333Gly in the literature) has been reported in the compound heterozygous state in one individual with familial hypercholesterolemia (FH) who had a second pathogenic variant in LDLR (Hobbs 1992) and in the heterozygous state in at least 6 individuals with FH (Hobbs 1992, Fouchier 2001, Chmara 2010, Pek 2017, ClinVar: Variation ID 251639). In vitro functional studies provide some evidence that the p.Asp354Gly variant may impact protein function (Hobbs 1992). This variant has also been identified in 1/30782 South Asian chromosomes by the Genome Aggregation Database (gnomAD, http://gnomad.broadinstitute.org/; dbSNP rs755449669). This frequency is low enough to be consistent with the frequency of FH in the general population. This variant is located in the first base of the exon, which is part of the 3'splice region. Computational prediction tools and conservation analysis suggest an impact both the protein and splicing. In summary, although additional studies are required to fully establish its clinical significance, the p.Asp354Gly variant is likely pathogenic. The ACMG/AMP Criteria applied: PM2; PP3; PS3_Supporting; PS4_Moderate.
19:11223944:G>A	*LDLR* splice site	c.1187-10G>A	1	The c.1187-10G>A variant in LDLR has been reported in 10 heterozygous individuals and 1 homozygous individual with hypercholesterolemia and segregated with disease in 10 affected individuals from 2 families (Wang 2011, Amsellem 2002, Punzalan 2005, Chmara 2010, Sun 2015, Liang 2016). It has also been identified in 0.005% (1/18364) of East Asian chromosomes by gnomAD (http://gnomad.broadinstitute.org) and is reported in ClinVar (Variation ID: 226349). This variant is located in the 3' splice region. Computational prediction tools and in vitro splicing assays are consistent with pathogenicity (Holla 2009). In vitro functional studies support an impact on protein function (Holla 2009, Romano 2011). In summary, this variant meets criteria to be classified as pathogenic for autosomal dominant hypercholesterolemia. ACMG/AMP Criteria applied: PP1_Strong, PM3, PS3_Moderate, PS4_Moderate.
19:11223983:C>T	*LDLR* Missense	p.Arg406Trp	1	The p.Arg406Trp variant in LDLR has been reported in >15 individuals with hypercholesterolemia and segregated with disease in >30 affected relatives from multiple families (Reshef 1996, Bourbon 2008, Chiou 2010, Shin 2015, Jannes 2015, Medeiros 2014, Medeiros 2015, and Benito-Vicente 2015, ClinVar submission accessions: SCV000503317.1, SCV000268604.1). It has been also identified in 2/24010 African chromosomes by the Genome Aggregation Database (gnomAD, http://gnomad.broadinstitute.org/). In vitro functional studies provide some evidence that the p.Arg406Trp variant diminishes the protein activity by ~40%, suggesting that it may be a milder variant (Benito-Vicente 2015) which is consistent with the milder than expected phenotype seen in a homozygote (Medeiros 2015). Computational prediction tools and conservation analysis suggest that the p.Arg406Trp variant may impact the protein. In summary, this variant meets criteria to be classified as pathogenic for familial hypercholesterolemia in an autosomal dominant manner. The ACMG/AMP Criteria applied: PP1_Strong, PS3_Supporting, PS4, PM2_Supporting, PP3.
19:11223989:G>A	*LDLR* Missense	p.Glu408Lys	1	The p.Glu408Lys variant in LDLR (also described as p.Glu387Lys in the literature), has been reported in one individual with early myocardial infarction (Do 2015) and at least 7 individuals with familial

				hypercholesterolemia (FH), including at least 3 heterozygotes, 3 homozygotes and 1 compound heterozygote with a second pathogenic variant in the LDLR gene (Hobbs 1992, Dedoussis 2004, Widhalm 2007, Taylor 2010). Other clinical laboratories have also reported this variant in ClinVar (Variation ID 36453). In vitro functional studies provide some evidence that the p.Glu408Lys variant may impact ligand recycling as it failed to release ligands in the endosome (Hobbs 1992, Strom 2011). This variant has also been identified in 2/111530 European chromosomes by the Genome Aggregation Database (GnomAD, http://gnomad.broadinstitute.org; dbSNP rs137943601). This frequency is low enough to be consistent with the frequency of FH in the general population. Additionally, other amino acid changes at this position have been reported in individuals with FH (Palcoux 2008, Duscova 2011), suggesting that changes at this position may not be tolerated. In summary, although additional studies are required to fully establish its clinical significance, the p.Glu408Lys variant is likely pathogenic. ACMG/AMP Criteria applied: PS4_moderate, PM2, PS3_supporting, PM5_supporting (Richards 2015).
19:11224052:G>A	*LDLR* Missense	p.Val429Met	1	The p.Val429Met variant in LDLR has been identified in over 25 individuals with familial hypercholesterolemia (FH; including in 2 homozygotes and 3 compound heterozygotes with a known pathogenic variant) and segregated with disease in over 100 affected relatives from at least 1 family (Leitersdorf 1989, Dedoussis 2004, Versmissen 2011). Additionally, this variant was also identified in at least 1 family (of 2 affected sibs) with premature myocardial infraction (MI; Braenne 2015). The p.Val429Met variant has been reported by other clinical laboratories in ClinVar (Variation ID: 3694) and has also been identified in 0.001% (3/251284) of pan-ethnic chromosomes by gnomAD (http://gnomad.broadinstitute.org). This frequency is low enough to be consistent with the frequency of FH in the general population. Computational prediction tools and conservation analysis do not provide strong support for or against an impact to the protein. In vitro functional studies support an impact on protein function (Leitersdorf 1989). In summary, this variant meets criteria to be classified as pathogenic for autosomal dominant FH. ACMG/AMP Criteria applied: PS4, PP1_Strong, PM2, PS3_Supporting.
19:11224266:G>T	*LDLR* Missense	p.Asp472Tyr	3	The p.Asp472Tyr variant in LDLR (also described as p.Asp451Tyr in the literature) has been reported in 10 individuals with familial hypercholesterolemia (FH) and 12 individuals who had a myocardial infarction, and segregated with disease in 3 affected relatives from 2 families (Abul-Husn 2016, Vohnout 2016, Thormaehlen 2015, Tichy 2012, Bertolini 2013, Do 2015, Campagna 2008). This variant has also been reported by other clinical laboratories in ClinVar (Variation ID 183116) and has been identified in 5/30778 of South Asian chromosomes and 8/126504 European chromosomes by the Genome Aggregation Database (gnomAD, http://gnomad.broadinstitute.org/; dbSNP 730882102). This frequency is low enough to be consistent with the frequency of FH in the general population. Computational prediction tools and conservation analysis do not provide strong support for or against an impact to the protein and in vitro functional assays were unclear in their overall impact (Thormaehlen 2015). In summary, although additional studies are required to fully establish its clinical significance, the p.Asp472Tyr variant is likely pathogenic. The ACMG/AMP Criteria applied (Richards 2015): PS4, PM2, PP1.
19:11224296:G>A	*LDLR* Missense	p.Asp482Asn	5	The p.Asp482Asn variant in LDLR has been reported in at least 24 heterozygous individuals and 1 compound heterozygous individual with hypercholesterolemia or early myocardial infarction and segregated with disease in 3 affected individuals from 2 families (Ward 1995, Webb 1996, Leren 2004, Graham 2005, Tichy 2012, Bertolini 2013, Lange 2014, Do 2015, Braenne 2015). It has also been identified in 0.009% (11/129018) of European chromosomes by gnomAD (http://gnomad.broadinstitute.org) and has been reported in ClinVar (Variation ID: 161284). Computational prediction tools and conservation analysis are consistent with pathogenicity. In vitro functional studies support an impact on protein function (Webb 1996). 3 variants at this position (p.Asp482Gly, p.Asp482His, p.Asp482Tyr) have been identified in patients with hypercholesterolemia, suggesting changes at this position may not be tolerated. In summary, this variant meets criteria to be

				classified as pathogenic for autosomal dominant hypercholesterolemia. ACMG/AMP Criteria applied: PS4, PM3, PP1, PP3, PS3_Supporting, PM5_Supporting.
19:11224326:G>A	*LDLR* Missense	p.Asp492Asn	1	The p.Asp492Asn variant in LDLR has been reported in >15 individuals across diverse populations with hypercholesterolemia (Mak 1998, Descamps 2001, Amsellem 2002, Damgaard 2005, Taylor 2007, Alonso 2009, Guardamagno 2009, Chiou 2010, Tichy 2012). In vitro functional studies provide some evidence that the p.Asp492Asn variant may not impact protein function (Thormaehlen 2015). However, these types of assays may not accurately represent biological function. This variant has been identified in 2/17248 East Asian chromosomes by the Genome Aggregation Database (gnomAD, http://gnomad.broadinstitute.org/; dbSNP rs373646964) and is reported in ClinVar (Variation ID: 251864). However, this frequency is not inconsistent for diseases with clinical variability, reduced penetrance, or recessive inheritance, pathogenic variants may be present at a low frequency in the general population. Computational prediction tools and conservation analysis suggest that the p.Asp492Asn variant may impact the protein. Two other variants at the same amino acid residue have been identified in patients with hypercholesterolemia (p.Asp492Gly and p.Asp492His), suggesting that variation in this position may not be tolerated. In summary, although additional studies are required to fully establish its clinical significance, the p.Asp492Asn variant is likely pathogenic. ACMG/AMP Criteria applied: PS4; PM2; PM5_Supporting; PP3.
19:11224419:G>A	*LDLR* Missense	p.Val523Met	1	The p.Val523Met variant in LDLR (also referred as p.Val502Met, FH Kuwait, and FH Bari-2) has been reported in >100 heterozygous individuals with hypercholesterolemia as well as in at least 3 homozygous individuals and 3 compound heterozygous individuals with homozygous familial hypercholesterolemia (Hobbs 1990, Tichy 2012, Bertolini 2013, Wang 2016, Sánchez-Hernández 2016, Pirillo 2017). This variant also segregated with homozygous familial hypercholesterolemia in 1 homozygous relative (Bertolini 2013). In vitro functional studies provide some evidence that the heterozygous p.Val523Met variant may impact protein function (Romano 2011). It has been reported in ClinVar (Variation ID: 3696) and has also been identified in 1/30782 South Asian and 2/111654 European chromosomes by the Genome Aggregation Database (gnomAD, http://gnomad.broadinstitute.org; dbSNP rs28942080). In summary, this variant meets criteria to be classified as pathogenic for familial hypercholesterolemia in an autosomal dominant manner based upon proband count, absence from controls, and functional studies. The ACMG/AMP Criteria applied: PS4, PM2, PS3_Supporting, PM3_Strong.
19:11224443:G>A	*LDLR* splice site	c.1586+5G>A	3	The c.1586+5G>A variant in LDLR has been reported in 7 individuals with familial hypercholesterolemia (FH) and segregated with disease in at least 7 affected relatives from 2 families (Ekstrom 1995, Jensen 1999, Fouchier 2005, Taylor 2007, Guardamagna 2009, Mollaki 2014). Additionally, this variant has been identified in 6/30752 South Asian chromosomes by the Genome Aggregation Database (gnomAD, http://gnomad.broadinstitute.org/) and is present in ClinVar (Variation ID: 251909). Please note that for diseases with clinical variability, reduced penetrance, or recessive inheritance, pathogenic variants may be present at a low frequency in the general population. In vitro functional studies provide some evidence that the c.1586+5G>A variant may impact protein function (Jensen 1999. This variant is located in the 5' splice region. Computational tools do suggest an impact to splicing. In summary, although additional studies are required to fully establish its clinical significance, the c.1586+5G>A variant is likely pathogenic for FH in an autosomal dominant manner based on cased observations, segregation studies, functional and computational evidence. The ACMG/AMP Criteria applied: PP1_Strong, PS4_Moderate, PP3, PS3_Supporting.
19:11226816:G>*	*LDLR* Frameshift	p.Gly546AlafsX2	1	The p.Gly546AlafsX2 variant in LDLR has been reported in 2 individuals with hypercholesterolemia and segregated with disease in 3 affected family members (Marduel 2010). It was absent from large population studies. This variant is predicted to cause a frameshift, which alters the protein's amino acid sequence beginning at position 546 and leads to a premature termination codon 2 amino acids downstream. This alteration is then predicted to lead to a truncated or absent protein. Loss of function of the LDLR gene is an established disease mechanism in autosomal dominant familial hypercholesterolemia. In summary, this variant meets criteria to be classified as pathogenic for

				autosomal dominant familial hypercholesterolemia. ACMG/AMP Criteria applied: PVS1, PM2, PP1, PS4_Supporting.
19:11226817:G>A	*LDLR* Missense	p.Gly545Glu	5	The p.Gly545Glu variant in LDLR has been reported in 1 large Pakistani family with familial hypercholesterolemia (FH) and segregated with disease in 3 homozygous and 17 heterozygous affected relatives (Ahmed 2013). Overall mean total cholesterol levels were significantly higher in those family members with the p.Gly545Glu variant compared to those without. Additionally, total cholesterol levels in homozygotes was significantly higher than heterozygotes. This variant has also been reported by other clinical laboratories in ClinVar (Variation ID: 251945) and has been identified in 0.007% (2/30616) of South Asian chromosomes by gnomAD (http://gnomad.broadinstitute.org). This frequency is low enough to be consistent with the frequency of FH in the general population. Computational prediction tools and conservation analyses do not provide strong support for or against an impact to the protein. In summary, although additional studies are required to fully establish its clinical significance, this variant meets criteria to be classified as likely pathogenic for autosomal dominant FH. ACMG/AMP Criteria applied: PP1_Strong, PM2.
19:11226820:G>T	*LDLR* Missense	p.Gly546Val	1	The p.Gly546Val variant in LDLR has been reported in 5 individuals with hypercholesterolemia (Koeijvoets 2005, Taylor 2007, Vaca 2011). It was absent from large population studies. Computational prediction tools and conservation analyses suggest that this variant may impact the protein, though this information is not predictive enough to determine pathogenicity. Another variant, p.Gly546Asp, involving this codon has also been identified in individuals with hypercholesterolemia. In summary, although additional studies are required to fully establish its clinical significance, this variant meets criteria to be classified as likely pathogenic for autosomal dominant familial hypercholesterolemia. ACMG/AMP Criteria applied: PM2, PM5, PP3, PS4_Supporting.
19:11227549:C>T	*LDLR* Missense	p.Arg574Cys	3	The p.Arg574Cys variant in LDLR has been reported in 10 individuals with familial hypercholesterolemia (FH) and segregated with disease in 3 affected individuals from at least 2 families (Nauck 2001, Chmara 2010, Bertolini 2013, Thromaehlen 2015, Sharifi 2016, Guardamagna 2009, Do 2015). It has also been identified in 8/129194 European chromosomes by gnomAD (http://gnomad.broadinstitute.org) and has been reported in ClinVar (Variation ID: 183123). Computational prediction tools and conservation analysis suggest that this variant may impact the protein, though this information is not predictive enough to determine pathogenicity. In vitro functional studies provide some evidence that this variant does not impact LDLR uptake in cells (Thromaehlen 2015); however, these types of assays may not accurately represent biological function. Three additional variants involving this codon (p.Arg574) have been identified in individuals with FH (Stenson 2017). In summary, while there is some suspicion for a pathogenic role, the clinical significance of this variant is uncertain. ACMG/AMP Criteria applied: PS4, PM2_Supporting, PP1, PP3.
19:11227576:C>T	*LDLR* Missense	p.His583Tyr	1	The p.His583Tyr variant in LDLR (also reported as p.His562Tyr in the literature) has been reported in at least 18 individuals with familial hypercholesterolemia (FH): 15 in the heterozygous state and 3 in the compound heterozygous state. It segregated with disease in 9 affected relatives from 3 families (Sun 1994, Punzalan 2005, Chiou 2012, Yao 2012, Ma 2017). Compound heterozygotes were more severely affected than heterozygotes in the same families. This variant has also been reported by other clinical laboratories in ClinVar (Variation ID: 200921) and has been identified in 0.13% (24/18868) of East Asian chromosomes by gnomAD (http://gnomad.broadinstitute.org). This frequency in low enough to be consistent with the frequency of FH in the general population. In vitro functional studies provide some evidence that the p.His583Tyr variant may impact protein processing (Sun 1994). Computational prediction tools and conservation analysis do not provide strong support for or against an impact to the protein. In summary, this variant meets criteria to be classified as pathogenic for FH in an autosomal dominant manner based upon occurrences in multiple affected individuals, segregation studies and functional evidence. The ACMG/AMP Criteria applied: PS4; PP1_Strong; PS3_Supporting.

19:11227612:C>T	*LDLR* Missense	p.Arg595Trp	1	The p.Arg595Trp variant in LDLR has been reported in at least 15 individuals with dominant or recessive familial hypercholesterolemia (Alonso 2016, Bañares 2017, Chiou 2011, Damgaard 2005, Descamps 2001, Fouchier 2005, Jannes 2015, Junyent 2010, Mozas 2004, Pek 2017, Pirillo 2017, Sánchez-Hernández 2016). It has also been identified in 2/129168 European chromosomes by gnomAD (http://gnomad.broadinstitute.org) and has also been reported in ClinVar (Variation ID: 161290). Computational prediction tools and conservation analysis suggest that this variant may impact the protein, though this information is not predictive enough to determine pathogenicity. Two other variants at this codon have been reported in individuals with familial hypercholesterolemia (Stenson 2017). In summary, this variant meets criteria to be classified as pathogenic for autosomal dominant familial hypercholesterolemia. ACMG/AMP Criteria applied: PM3_VeryStrong, PS4, PM2, PP3.
19:11230819:C>T	*LDLR* Missense	p.Arg633Cys	2	The p.Arg633Cys variant in LDLR has been reported in at least 11 individuals with familial hypercholesterolemia (FH), including one homozygote (Day 1997, Mozas 2004, Guardamagna 2009, Taylor 2009, Chiou 2011, Tichy 2012, Bertolini 2013, Xiang 2017, Medieros 2017). This variant has been reported in ClinVar (Variation ID: 226379) and has also been identified in 1/30782 South Asian chromosomes by the Genome Aggregation Database (gnomAD, http://gnomad.broadinstitute.org). Computational prediction tools and conservation analysis suggest that the p.Arg833Cys variant may impact the protein. Two other variants at this amino acid position have been reported in at least 4 individuals with FH (p.Arg633Leu and p.Arg633His). In summary, although additional studies are required to fully establish its clinical significance, the p.Arg633Cys variant is likely pathogenic. ACMG/AMP Criteria applied: PS4_Moderate, PM2, PM5_Supporting, PP3.
19:11230820:G>A	*LDLR* Missense	p.Arg633His	1	The p.Arg633His variant in LDLR (also described as p.Arg612His in the literature) has been reported in 7 individuals with familial hypercholesterolemia (FH), including 2 compound heterozygotes (Fouchier 2005, Damgaard 2005, Huijgen 2012, Alonso 2016, ClinVar submission accession: SCV000583907.1, SCV000268649.1). It has also been reported by other clinical laboratories in ClinVar (Variation IDL 226380) and has been identified in 0.002% (6/282886) of pan-ethnic chromosomes by gnomAD (http://gnomad.broadinstitute.org). This frequency is low enough to be consistent with the frequency of FH in the general population. Computational prediction tools and conservation analyses are consistent with pathogenicity. Another variant involving this codon (p.Arg633Cys) has been identified in individuals with FH and is classified as likely pathogenic by this laboratory, suggesting change at this position may not be tolerated. In summary, although additional studies are required to fully establish its clinical significance, this variant meets criteria to be classified as likely pathogenic for autosomal dominant FH. ACMG/AMP Criteria applied: PM2, PM5_Supporting, PP3, PS4_Moderate.
19:11231095:T>A	*LDLR* Nonsense	p.Tyr679X	1	The p.Tyr679X variant in LDLR has been reported in 1 individual with hypercholesterolemia (Taylor 2007). It was absent from large population studies. This nonsense variant leads to a premature termination codon at position 679, which is predicted to lead to a truncated or absent protein. Loss of function of the LDLR gene is an established disease mechanism in autosomal dominant familial hypercholesterolemia. In summary, although additional studies are required to fully establish its clinical significance, this variant meets criteria to be classified as likely pathogenic for autosomal dominant familial hypercholesterolemia. ACMG/AMP Criteria applied: PVS1, PM2.
19:11231112:C>T	*LDLR* Missense	p.Pro685Leu	6	The p.Pro685Leu variant in LDLR, also described as p.Pro664Leu in the literature, has been reported in >40 individuals with familial hypercholesterolemia (FH), segregated with disease in >40 affected relatives from at least 4 families, and was identified in the homozygous state in at least 10 individuals with FH (Bertolini 2013, Sharifi 2016, Medeiros 2010,Rubinsztein 1992, Soutar 1989, Soutar 1991, Thormaehlen 2015, Van Der Graaf 2011). This variant has also been reported by other clinical laboratories in Clinvar (Variation ID 3702) and was identified in 6/126656 European chromosomes by the Genome Aggregation Database (gnomAD, http://gnomad.broadinstitute.org; dbSNP rs28942084). However, this frequency is low enough to be consistent with the frequency of FH in the general population. In vitro functional studies provide some evidence that the p.Pro685Leu variant may impact

				protein function (Knight 1989, Rubinsztein 1992, Thormaehlen 2015). In summary, this variant meets criteria to be classified as pathogenic for familial hypercholesterolemia in an autosomal dominant manner based upon presence in multiple affected individuals, segregation studies, low frequency in controls and functional evidence. The ACMG/AMP Criteria applied: PS4, PP1_Strong, PM2, PS3_Supporting.
19:11231118:*>C	*LDLR* Frameshift	p.Asn688GlnfsX29	1	The p.Asn688Glnfsx29 variant in LDLR has been identified in at least 10 individuals hypercholesterolemia (Graham 2005 Humphries 2006, Hooper 2012, Vandrovcova 2013, Johnston 2015, Abul-Husn 2016, Martin 2016) and has also been reported in ClinVar (Variation ID: 68103). It has been identified in 1/113654 European chromosomes by gnomAD (http://gnomad.broadinstitute.org). This variant is predicted to cause a frameshift, which alters the protein's amino acid sequence beginning at position 688 and leads to a premature termination codon 29 amino acids downstream. This alteration is then predicted to lead to a truncated or absent protein. Heterozygous loss of function of the LDLR gene is an established disease mechanism in familial hypercholesterolemia. In summary, this variant meets criteria to be classified as pathogenic. ACMG/AMP Criteria applied: PVS1, PS4_Moderate, PM2.
Hereditary breast and ovarian cancer variants				
13:32893317:C>G	*BRCA2* Nonsense	p.Tyr57X	1	The p.Tyr57X variant in BRCA2 has been reported in the literature in several individuals with unspecified phenotype (van der Hout 2006, Heramb 2018, Rebbeck 2018). It has also been reported in individuals with hereditary breast and/or ovarian cancer (HBOC) in ClinVar (Variation ID: 51179). Furthermore, this variant was classified as Pathogenic by the ClinGen-approved ENIGMA expert panel (SCV000607880.2). It was absent from large population studies. This nonsense variant leads to a premature termination codon at position 57, which is predicted to lead to a truncated or absent protein. Loss of function of the BRCA2 gene is an established disease mechanism in autosomal dominant HBOC. In summary, this variant meets criteria to be classified as pathogenic for autosomal dominant HBOC. ACMG/AMP Criteria applied: PVS1, PM2, PS4_Supporting.
13:32900420:G>T	*BRCA2* splice site	c.516+1G>T	1	The c.516+1G>T variant in BRCA2 has been reported in the literature in at least 1 individual with ovarian cancer (Weren 2017), and has also been reported in individuals with hereditary breast and ovarian cancer (HBOC) in ClinVar (Variation ID: 267649). It was absent from large population studies. This variant occurs within the canonical splice site (+/- 1,2) and is predicted to cause altered splicing leading to an abnormal or absent protein. Furthermore, in vitro functional studies support that this variant leads to abnormal splicing (Whiley 2011). In summary, this variant meets criteria to be classified as pathogenic for autosomal dominant HBOC. ACMG/AMP Criteria applied: PVS1, PM2, PS3_Supporting.
13:32900691:A>G	*BRCA2* Missense	p.Asp191Gly	1	The p.Asp191Gly variant in BRCA2 has been reported in 2 individuals with early-onset breast cancer (Gaildrat 2012). It was absent from large population studies. Computational prediction tools and conservation analysis suggest that the p.Asp191Gly variant may impact the protein, though this information is not predictive enough to determine pathogenicity. Additionally, in vitro splicing assays provide evidence that this variant leads to a splicing change, resulting in the in-frame deletion of 20 amino acids (Gaildrat 2012, Fraile-Bethencourt 2019); however, these types of assays may not accurately represent biological function. In summary, although additional studies are required to fully establish its clinical significance, this variant meets criteria to be classified as likely pathogenic for autosomal dominant hereditary breast and ovarian cancer syndrome (HBOC). ACMG/AMP Criteria applied: PM2, PS3_Moderate, PP3, PS4_Supporting.
13:32900720:CCAC>*	*BRCA2* Frameshift	p.Thr203LeufsX7	5	The p.Thr203LeufsX7 variant in BRCA2 has not been previously reported in individuals with hereditary breast and/or ovarian cancer (HBOC) and was absent from large population studies. This variant is predicted to cause a frameshift, which alters the protein's amino acid sequence beginning at position 203 and leads to a premature termination codon 7 amino acids downstream. This alteration is then predicted to lead to a truncated or absent protein. Loss of function of the BRCA2 gene is an

				established disease mechanism in autosomal dominant HBOC. In summary, although additional studies are required to fully establish its clinical significance, this variant meets criteria to be classified as likely pathogenic for autosomal dominant HBOC. ACMG/AMP Criteria applied: PVS1, PM2.
13:32905124:GACA>*	*BRCA2* Frameshift	p.Asp252ValfsX24	1	The p.Asp252ValfsX24 variant in BRCA2 has been reported in >60 individuals with BRCA2-related cancers and segregated with disease in at least 10 affected members of one family (Tavitigian 1996, Schrader 2016, Park 2017a, Park 2017b, Wang 2018, BIC database). Additionally, it was classified as Pathogenic on Apr. 22, 2016 by the ClinGen-approved ENIGMA expert panel (ClinVar Variation ID: 38103). This variant has also been identified in 0.005% (1/18384) of East Asian chromosomes by gnomAD (http://gnomad.broadinstitute.org); however, this frequency is low enough to be consistent with the frequency of hereditary breast and ovarian cancer (HBOC) in the general population. This variant is predicted to cause a frameshift, which alters the protein's amino acid sequence beginning at position 252 and leads to a premature termination codon 24 amino acids downstream. This alteration is then predicted to lead to a truncated or absent protein. Loss of function of the BRCA2 gene is an established disease mechanism in autosomal dominant hereditary breast and ovarian cancer syndrome (HBOC). In summary, this variant meets criteria to be classified as pathogenic for autosomal dominant HBOC. ACMG/AMP Criteria applied: PVS1, PS4, PP1_Strong, PM2_Supporting.
13:32905147:AA>*	*BRCA2* Frameshift	p.Glu260SerfsX15	3	The p.Glu260SerfsX15 variant in BRCA2 has been reported in at least 3 individuals with breast or ovarian cancer (Chao 2016, Wen 2018, Couch 2015). Additionally, it was classified as Pathogenic on Sep. 8, 2016 by the ClinGen-approved ENIGMA expert panel (ClinVar Variation ID: 188425). It was absent from large population studies. This variant is predicted to cause a frameshift, which alters the protein's amino acid sequence beginning at position 260 and leads to a premature termination codon 15 amino acids downstream. This alteration is then predicted to lead to a truncated or absent protein. Loss of function of the BRCA2 gene is an established disease mechanism in autosomal dominant hereditary breast and ovarian cancer syndrome (HBOC). In summary, this variant meets criteria to be classified as pathogenic for autosomal dominant HBOC. ACMG/AMP Criteria applied: PVS1, PM2, PS4_Supporting.
13:32906407:A>G	*BRCA2* splice site	c.794-2A>G	3	The c.794-2A>G variant in BRCA2 has not been previously reported in individuals with hereditary breast and/or ovarian cancer (HBOC) and was absent from large population studies, but has been reported in ClinVar (Variation ID: 568479). This variant occurs within the canonical splice site (+/- 1,2) and is predicted to cause altered splicing leading to an abnormal or absent protein. Loss of function of the BRCA2 gene is an established disease mechanism in autosomal dominant HBOC. In summary, although additional studies are required to fully establish its clinical significance, this variant meets criteria to be classified as likely pathogenic for autosomal dominant HBOC. ACMG/AMP Criteria applied: PVS1, PM2.
13:32906872:T>*	*BRCA2* Frameshift	p.Cys419TrpfsX11	1	The p.Cys419TrpfsX11 variant in BRCA2 has been reported in at least 8 individuals with a personal or family history of breast or ovarian cancer (Lecarpentier 2012, Wong-Brown 2015, Lubinski 2004, BIC database). Additionally, it was classified as Pathogenic on Sep. 8, 2016 by the ClinGen-approved ENIGMA expert panel (ClinVar Variation ID: 37733). This variant was absent from large population studies. The p.Cys419TrpfsX11 variant is predicted to cause a frameshift, which alters the protein's amino acid sequence beginning at position 419 and leads to a premature termination codon 11 amino acids downstream. This alteration is then predicted to lead to a truncated or absent protein. Loss of function of the BRCA2 gene is an established disease mechanism in autosomal dominant hereditary breast and ovarian cancer syndrome (HBOC). In summary, this variant meets criteria to be classified as pathogenic for autosomal dominant HBOC. ACMG/AMP Criteria applied: PVS1, PM2, PS4_Moderate.
13:32906916:AAAG>*	*BRCA2* Frameshift	p.Lys437IlefsX22	1	The p.Lys437IlefsX22 variant in BRCA2 has been reported in >30 individuals with BRCA2-related cancers (Gayther 1997, Caputo 2012, Laarabi 2017, BIC database). Additionally, it was classified as Pathogenic on Apr. 22, 2016 by the ClinGen-approved ENIGMA expert panel (ClinVar Variation ID: 37737). This variant has also been identified in 0.006% (1/15544) of African chromosomes by gnomAD (http://gnomad.broadinstitute.org); however, this frequency is low enough to be consistent

				with the frequency of hereditary breast and ovarian cancer (HBOC) in the general population. The p.Lys437IlefsX22 variant is predicted to cause a frameshift, which alters the protein's amino acid sequence beginning at position 437 and leads to a premature termination codon 22 amino acids downstream. This alteration is then predicted to lead to a truncated or absent protein. Loss of function of the BRCA2 gene is an established disease mechanism in HBOC. In summary, this variant meets criteria to be classified as pathogenic for autosomal dominant HBOC. ACMG/AMP Criteria applied: PVS1, PM2, PS4.
13:32906974:A>*	*BRCA2* Frameshift	p.Lys454AsnfsX6	1	The p.Lys454AsnfsX6 variant in BRCA2 has been reported in 1 individual with breast cancer (Fackenthal 2012). Additionally, it was classified as Pathogenic on Oct. 18, 2016 by the ClinGen-approved ENIGMA expert panel (Variation ID: 51109). This variant was absent from large population studies. The p.Lys454AsnfsX6 variant is predicted to cause a frameshift, which alters the protein's amino acid sequence beginning at position 454 and leads to a premature termination codon 6 amino acids downstream. This alteration is then predicted to lead to a truncated or absent protein. Loss of function of the BRCA2 gene is an established disease mechanism in autosomal dominant hereditary breast and ovarian cancer syndrome (HBOC). In summary, although additional studies are required to fully establish its clinical significance, this variant meets criteria to be classified as likely pathogenic for autosomal dominant HBOC. ACMG/AMP Criteria applied: PVS1, PM2.
13:32907419:G>T	*BRCA2* Nonsense	p.Gly602X	1	The p.Gly602X variant in BRCA2 has not been previously reported in individuals with hereditary breast and/or ovarian cancer (HBOC) and was absent from large population studies. This nonsense variant leads to a premature termination codon at position 602, which is predicted to lead to a truncated or absent protein. Loss of function of the BRCA2 gene is an established disease mechanism in autosomal dominant HBOC. In summary, although additional studies are required to fully establish its clinical significance, this variant meets criteria to be classified as likely pathogenic for autosomal dominant HBOC. ACMG/AMP Criteria applied: PVS1, PM2.
13:32907455:*>T	*BRCA2* Nonsense	p.Asn615X	1	The p.Asn615X (c.1842dupT) variant in BRCA2 has been reported in at least 3 individuals with BRCA2-related cancers (Esteban Cardeñosa 2010, BIC database). Additionally, it was classified as Pathogenic on Apr 22, 2016 by the ClinGen-approved ENIGMA expert panel (ClinVar Variation ID: 51212). This variant was absent from large population studies. The p.Asn615X (c.1842dupT) variant is an insertion of a single nucleotide, creating a premature termination codon at position 57, which is predicted to lead to a truncated or absent protein. Loss of function of the BRCA2 gene is an established disease mechanism in autosomal dominant hereditary breast and ovarian cancer syndrome (HBOC). In summary, this variant meets criteria to be classified as pathogenic for autosomal dominant HBOC. ACMG/AMP Criteria applied: PVS1, PM2, PS4_Supporting.
13:32910421:G>*	*BRCA2* Frameshift	p.Arg645GlufsX15	1	The p.Arg645GlufsX15 variant in BRCA2 has been reported in >50 individuals with BRCA2-associated cancers (Evans, 2004, Janavicius 2010, Breast Cancer Information Core (BIC) database). This variant was absent from large population studies, though the ability of these studies to accurately detect indels may be limited. This variant is predicted to cause a frameshift, which alters the protein's amino acid sequence beginning at position 645 and leads to a premature termination codon 15 amino acids downstream. This alteration is then predicted to lead to a truncated or absent protein. Heterozygous loss of function of the BRCA2 gene is an established disease mechanism for hereditary breast and ovarian cancer (HBOC). In summary, this variant meets our criteria to be classified as pathogenic for HBOC in an autosomal dominant manner based upon the predicted impact to the protein. ACMG/AMP criteria applied: PVS1, PS4, PM2.
13:32910821:*>A	*BRCA2* Frameshift	p.Asp777GlufsX11	1	The p.Asp777GlufsX11 variant in BRCA2 has been reported in >15 individuals with BRCA2-related cancers (Agoff 2002, Edwards 2003, Lowery 2018, Castro 2013, Alsop 2012, Oros 2006, BIC database). It has also been identified in 1/113388 European chromosomes by gnomAD (http://gnomad.broadinstitute.org); however, this frequency is low enough to be consistent with the frequency of hereditary breast and ovarian cancer (HBOC) in the general population. This variant is predicted to cause a frameshift, which alters the protein's amino acid sequence beginning at position 777 and leads to a premature termination codon 11 amino acids downstream. This alteration is then

				predicted to lead to a truncated or absent protein. Loss of function of the BRCA2 gene is an established disease mechanism in autosomal dominant hereditary breast and ovarian cancer syndrome (HBOC). Additionally, this variant was classified as Pathogenic on Sept 8 2016 by the ClinGen-approved ENIGMA expert panel (Variation ID: 91775). In summary, this variant meets criteria to be classified as pathogenic for autosomal dominant HBOC. ACMG/AMP Criteria applied: PVS1, PM2, PS4.
13:32910899:*>A	*BRCA2* Nonsense	p.Tyr803X	1	The p.Tyr803X (c.2408_2409insA) variant in BRCA2 has not been reported in individuals with BRCA2-related cancers. It was absent from large population studies. This variant is a deletion of a single nucleotide, creating a premature termination codon at position 803, which is predicted to lead to a truncated or absent protein. Loss of function of the BRCA2 gene is an established disease mechanism in autosomal dominant hereditary breast and ovarian cancer (HBOC). Furthermore, a different variant resulting in a termination codon at the same position (c.2409T>G, p.Tyr803X) has been classified as Pathogenic by our laboratory and multiple submitters in ClinVar, including the ClinGen-approved ENIGMA expert panel (ClinVar Variation ID 37784). In summary, the p.Tyr803X (c.2408_2409insA) variant meets criteria to be classified as pathogenic for autosomal dominant HBOC. ACMG/AMP Criteria applied: PVS1, PS1, PM2.
13:32910901:T>G	*BRCA2* Nonsense	p.Tyr803X	1	The p.Tyr803X variant in BRCA2 has been reported in at least 2 individuals with BRCA2-associated cancers (Breast Cancer Information Core (BIC) database, Zhang 2011) and was absent from large population studies. This nonsense variant leads to a premature termination codon at position 803, which is predicted to lead to a truncated or absent protein. Heterozygous loss of function of the BRCA2 gene is an established disease mechanism in hereditary breast and ovarian cancer (HBOC). In addition, this variant was classified as Pathogenic on September 8, 2016 by the ClinGen-approved ENIGMA expert panel (ClinVar SCV000300516.2). In summary, this variant meets our criteria to be classified as pathogenic for HBOC in an autosomal dominant manner based upon the predicted impact to the protein. ACMG/AMP Criteria applied: PVS1, PM2, PS4_Supporting.
13:32911098:C>G	*BRCA2* Nonsense	p.Ser869X	1	The p.Ser869X variant in BRCA2 has not been previously reported in individuals with hereditary breast and/or ovarian cancer (HBOC) and was absent from large population studies, but has been reported in ClinVar (Variation ID: 252823). This nonsense variant leads to a premature termination codon at position 869, which is predicted to lead to a truncated or absent protein. Loss of function of the BRCA2 gene is an established disease mechanism in autosomal dominant HBOC. In summary, although additional studies are required to fully establish its clinical significance, this variant meets criteria to be classified as likely pathogenic for autosomal dominant HBOC. ACMG/AMP Criteria applied: PVS1, PM2.
13:32911298:AAAC>*	*BRCA2* Frameshift	p.Ala938ProfsX21	1	The p.Ala938ProfsX21 variant in BRCA2 is a well-established pathogenic variant for hereditary breast and ovarian cancer (HBOC) and is one of the most common germline mutations in non-Ashkenazi Jewish individuals with breast cancer (Gao 2000, Diez 2003, Janavicius 2010, Caputo 2012, Kwong 2012, Infante 2013). This variant has also been identified in one male with prostate cancer and 5 males with breast cancer (Edwards 2010, de Juan 2015). Furthermore, this variant was identified in 2/113374 of European chromosomes by gnomAD (http://gnomad.broadinstitute.org). This variant was classified as Pathogenic on April 22, 2016 by the ClinGen-approved Evidence-based Network for the Interpretation of Germline Mutant Alleles (ENIGMA) expert panel (Variation ID 9322). The p.Ala938ProfsX21 variant is predicted to cause a frameshift, which alters the protein's amino acid sequence beginning at position 938 and leads to a premature termination codon 21 amino acids downstream. This alteration is then predicted to lead to a truncated or absent protein. Heterozygous loss of function of the BRCA2 gene is an established disease mechanism for HBOC. In summary, this variant meets our criteria to be classified as pathogenic for autosomal dominant HBOC. ACMG/AMP Criteria applied: PVS1, PS4, PM2.
13:32911322:A>T	*BRCA2* Nonsense	p.Lys944X	1	The p.Lys944X variant in BRCA2 has been reported in at least 11 individuals with BRCA2-related cancers; however two of these individuals harbored pathogenic variants in BRCA1 as well (Hakansson 1997, Heidemann 2012, Vietri 2013, Susswein 2016, Weren 2017, Lowery 2018, Wen

				2018, BIC database). In addition, this variant was reported in one proband with Fanconi anemia who was compound heterozygous for this variant and a second variant in BRCA2 (Bodd 2010). The p.Lys944X variant has also been identified in 0.004% (1/24868) of African chromosomes by gnomAD (http://gnomad.broadinstitute.org); however, this frequency is low enough to be consistent with the frequency of hereditary breast and ovarian cancer (HBOC) in the general population. This nonsense variant is predicted to lead to a premature termination codon at position 944. Loss of function of the BRCA2 gene is an established disease mechanism in autosomal dominant HBOC. Additionally, this variant was classified as Pathogenic on Sep 8, 2016 by the ClinGen-approved ENIGMA expert panel (Variation ID: 51355). In summary, this variant meets criteria to be classified as pathogenic for autosomal dominant HBOC. ACMG/AMP Criteria applied: PVS1, PS4_Moderate, PP1.
13:32911322:AA>*	*BRCA2* Frameshift	p.Lys945ArgfsX13	1	The p.Lys945ArgfsX13 variant in BRCA2 has not been previously reported in individuals with hereditary breast and/or ovarian cancer (HBOC) and was absent from large population studies, but has been reported in ClinVar (Variation ID: 55791). This variant is predicted to cause a frameshift, which alters the protein's amino acid sequence beginning at position 945 and leads to a premature termination codon 13 amino acids downstream. This alteration is then predicted to lead to a truncated or absent protein. Loss of function of the BRCA2 gene is an established disease mechanism in autosomal dominant HBOC. In summary, although additional studies are required to fully establish its clinical significance, this variant meets criteria to be classified as likely pathogenic for autosomal dominant HBOC. ACMG/AMP Criteria applied: PVS1, PM2.
13:32911595:G>T	*BRCA2* Nonsense	p.Glu1035X	1	The p.Glu1035X variant in BRCA2 has been reported in at least 8 individuals with BRCA2-related cancers (Ramus 2007, Tung 2015, Meric-Bernstam 2016, Susswein 2016, BIC database). It has also been identified in 1/112890 European chromosomes by gnomAD (http://gnomad.broadinstitute.org); however, this frequency is low enough to be consistent with the frequency of hereditary breast and ovarian cancer (HBOC) in the general population. This variant leads to a premature termination codon at position 1035, which is predicted to lead to a truncated or absent protein. Loss of function of the BRCA2 gene is an established disease mechanism in autosomal dominant hereditary breast and ovarian cancer syndrome (HBOC). Additionally, this variant was classified as Pathogenic on Apr 22, 2016 by the ClinGen-approved ENIGMA expert panel (Variation ID: 51400). In summary, this variant meets criteria to be classified as pathogenic for autosomal dominant HBOC. ACMG/AMP Criteria applied: PVS1, PM2, PS4_Moderate.
13:32911650:T>G	*BRCA2* Nonsense	p.Leu1053X	5	The p.Leu1053X variant in BRCA2 has been reported in at least 11 individuals with BRCA2-related cancers (Lubinski 2004, Kote-Jarai 2011, Elimam 2017, Mijuskovic 2018, Sandhu 2013, BIC database) and was absent from large population studies. This nonsense variant creates a premature termination codon at position 1053, which is predicted to lead to a truncated or absent protein. Loss of function of the BRCA2 gene is an established disease mechanism in autosomal dominant hereditary breast and ovarian cancer syndrome (HBOC). Additionally, this variant was classified as pathogenic on Apr 22, 2016 by the ClinGen-approved ENIGMA expert panel (ClinVar Variation ID: 37820). In summary, this variant meets criteria to be classified as pathogenic for autosomal dominant HBOC. ACMG/AMP Criteria applied: PVS1, PM2, PS4_Moderate.
13:32911897:C>A	*BRCA2* Nonsense	p.Tyr1135X	5	The p.Tyr1135X variant in BRCA2 has been reported in one individual with prostate cancer (Kote-Jarai 2011). It was absent from large population studies. This variant has also been reported in ClinVar (Variation ID: 231604). This nonsense variant leads to a premature termination codon at position 1135, which is predicted to lead to a truncated or absent protein. Loss of function of the BRCA2 gene is an established disease mechanism in autosomal dominant hereditary breast and ovarian cancer syndrome (HBOC). In summary, although additional studies are required to fully establish its clinical significance, this variant meets criteria to be classified as likely pathogenic for autosomal dominant HBOC. ACMG/AMP Criteria applied: PVS1, PM2.
13:32912036:TT>*	*BRCA2* Nonsense	p.Phe1182X	2	The p.Phe1182X variant in BRCA2 has been reported in >15 individuals with BRCA2-associated cancers (Breast Cancer Information Core (BIC) database, Lubinski 2004, Borg 2010, Tea 2014, Belanger 2015, Polsler 2016). It has also been identified in 5/113332 European chromosomes by the

				Genome Aggregation Database (GnomAD, http://gnomad.broadinstitute.org); however, this frequency is low enough to be consistent with the frequency of hereditary breast and ovarian cancer (HBOC) in the general population. This variant creates a premature termination codon at position 1182, which is predicted to lead to a truncated or absent protein. Heterozygous loss of function of the BRCA2 gene is an established disease mechanism in HBOC. Furthermore, the p.Phe1182X variant was classified as Pathogenic on April 22, 2016 by the ClinGen-approved ENIGMA expert panel (ClinVar SCV000282379.1). In summary, this variant meets criteria to be classified as pathogenic for HBOC in an autosomal dominant manner. ACMG/AMP criteria applied: PVS1, PS4.
13:32912090:TG>*	*BRCA2* Nonsense	p.Cys1200X	2	The p.Cys1200X variant in BRCA2 (resulting from c.3599_3600delGT) has been reported in at least 13 individuals with BRCA2-related cancers (De Leon Matsuda 2002, Cunningham 2014, Susswein 2016, Hirasawa 2017, Momozawa 2018, BIC database). It has also been identified in 1/18394 East Asian chromosomes by gnomAD (http://gnomad.broadinstitute.org); however, this frequency is low enough to be consistent with the frequency of hereditary breast and ovarian cancer (HBOC) in the general population. This frameshift variant is predicted to lead to a premature termination codon at position 1200. Loss of function of the BRCA2 gene is an established disease mechanism in autosomal dominant hereditary breast and ovarian cancer syndrome (HBOC). Additionally, this variant was classified as Pathogenic on Apr 22, 2016 by the ClinGen-approved ENIGMA expert panel (Variation ID: 51493). In summary, this variant meets criteria to be classified as pathogenic for autosomal dominant HBOC. ACMG/AMP Criteria applied: PVS1, PM2, PS4_Moderate.
13:32912172:TG>*	*BRCA2* Frameshift	p.Leu1227GlnfsX5	4	The p.Leu1227GlnfsX5 variant in BRCA2 has been reported in at least 8 individuals with BRCA2-associated cancers (Peto 1999, Risch 2001, Pal 2014, Breast Cancer Information Core (BIC) database). It has also been identified in 1/8710 African chromosomes by gnomAD (http://gnomad.broadinstitute.org); however, this frequency is low enough to be consistent with the frequency of hereditary breast and ovarian cancer (HBOC) in the general population. This variant is predicted to cause a frameshift, which alters the protein's amino acid sequence beginning at position 1227 and leads to a premature termination codon 5 amino acids downstream. This alteration is then predicted to lead to a truncated or absent protein. Heterozygous loss of function of the BRCA2 gene is an established disease mechanism in hereditary breast and ovarian cancer (HBOC). In addition, this variant was classified as Pathogenic on April 22, 2016 by the ClinGen-approved ENIGMA expert panel (ClinVar SCV000282380.1). In summary, this variant meets our criteria to be classified as pathogenic for HBOC in an autosomal dominant manner based upon the predicted impact to the protein. ACMG/AMP criteria applied: PVS1, PS4_Moderate, PM2.
13:32912207:A>*	*BRCA2* Frameshift	p.Lys1239AsnfsX20	1	The p.Lys1239AsnfsX20 variant in BRCA2 (also referred to in the literature as c.3745delA) has been reported in at least 10 individuals with breast cancer and segregated with disease in at least 15 affected individuals from 2 families (Tchou 2007, Tai 2007, BIC database). It was absent from large population studies. This variant is predicted to cause a frameshift, which alters the protein's amino acid sequence beginning at position 1239 and leads to a premature termination codon 20 amino acids downstream. This alteration is then predicted to lead to a truncated or absent protein. Loss of function of the BRCA2 gene is an established disease mechanism in autosomal dominant hereditary breast and ovarian cancer syndrome (HBOC). Additionally, this variant was classified as Pathogenic on Sept 8 2016 by the ClinGen-approved ENIGMA expert panel (Variation ID: 37855). In summary, this variant meets criteria to be classified as pathogenic for autosomal dominant HBOC. ACMG/AMP Criteria applied: PVS1, PP1_Strong, PM2.
13:32912277:C>G	*BRCA2* Nonsense	p.Ser1262X	1	The p.Ser1262X variant in BRCA2 has been reported in at least 6 individuals with BRCA2-related cancers (Song 2014, de Juan Jimenez 2013, Loughrey 2008, BIC database). It has also been identified in 1/110430 European chromosomes by gnomAD (http://gnomad.broadinstitute.org); however, this frequency is low enough to be consistent with the frequency of hereditary breast and ovarian cancer (HBOC) in the general population. This variant leads to a premature termination codon at position 1262, which is predicted to lead to a truncated or absent protein. Loss of function of the BRCA2 gene is an established disease mechanism in autosomal dominant hereditary breast and

				ovarian cancer syndrome (HBOC). Additionally, this variant was classified as Pathogenic on Apr 22, 2016 by the ClinGen-approved ENIGMA expert panel (Variation ID: 51525). In summary, this variant meets criteria to be classified as pathogenic for autosomal dominant HBOC. ACMG/AMP Criteria applied: PVS1, PM2, PS4_Moderate.
13:32912338:TG>*	*BRCA2* Frameshift	p.Val1283LysfsX2	5	The p.Val1283LysfsX2 variant in BRCA2 has been reported in >35 individuals with BRCA2-associated cancers (Wang 2012, Breast Cancer Information Core (BIC) database). This variant has also been identified in 11/112032 European chromosomes by the Genome Aggregation Database (GnomAD, http://gnomad.broadinstitute.org); however, this frequency is low enough to be consistent with the frequency of hereditary breast and ovarian cancer (HBOC) in the general population. This variant is predicted to cause a frameshift, which alters the protein's amino acid sequence beginning at position 1283 and leads to a premature termination codon 2 amino acids downstream. This alteration is then predicted to lead to a truncated or absent protein. Heterozygous loss of function of the BRCA2 gene is an established disease mechanism for HBOC. In summary, this variant meets our criteria to be classified as pathogenic for HBOC in an autosomal dominant manner based upon the predicted impact to the protein. ACMG/AMP criteria applied: PVS1, PS4.
13:32912364:A>*	*BRCA2* Frameshift	p.Gln1291HisfsX2	1	The p.Gln1291HisfsX2 variant in BRCA2 (also referred to in the literature as c.4101delA) has been reported in at least 3 individuals with BRCA2-related cancers (Young 2018, Song 2014, Foley 2015). It was absent from large population studies. This variant is predicted to cause a frameshift, which alters the protein's amino acid sequence beginning at position 1291 and leads to a premature termination codon 2 amino acids downstream. This alteration is then predicted to lead to a truncated or absent protein. Loss of function of the BRCA2 gene is an established disease mechanism in autosomal dominant hereditary breast and ovarian cancer syndrome (HBOC). Additionally, this variant was classified as Pathogenic on Sept 8 2016 by the ClinGen-approved ENIGMA expert panel (Variation ID: 91809). In summary, this variant meets criteria to be classified as pathogenic for autosomal dominant HBOC. ACMG/AMP Criteria applied: PVS1, PM2, PS4_Supporting.
13:32912655:C>*	*BRCA2* Frameshift	p.Thr1388IlefsX22	1	The p.Thr1388IlefsX22 variant in BRCA2 has not been previously reported in individuals with hereditary breast and/or ovarian cancer (HBOC) and was absent from large population studies. This variant is predicted to cause a frameshift, which alters the protein's amino acid sequence beginning at position 1388 and leads to a premature termination codon 22 amino acids downstream. This alteration is then predicted to lead to a truncated or absent protein. Loss of function of the BRCA2 gene is an established disease mechanism in autosomal dominant HBOC. In summary, although additional studies are required to fully establish its clinical significance, this variant meets criteria to be classified as likely pathogenic for autosomal dominant HBOC. ACMG/AMP Criteria applied: PVS1, PM2.
13:32912767:*>A	*BRCA2* Frameshift	p.Thr1426AsnfsX12	2	The p.Thr1426AsnfsX12 variant in BRCA2 (resulting from c.4276dupA) has been reported in at least 5 individuals with BRCA2-related cancers (Lubinski 2004, Crawford 2017, BIC database). It was absent from large population studies. This variant is predicted to cause a frameshift, which alters the protein's amino acid sequence beginning at position 1426 and leads to a premature termination codon 12 amino acids downstream. This alteration is then predicted to lead to a truncated or absent protein. Loss of function of the BRCA2 gene is an established disease mechanism in autosomal dominant hereditary breast and ovarian cancer syndrome (HBOC). Additionally, this variant was classified as Pathogenic on Sept 8 2016 by the ClinGen-approved ENIGMA expert panel (Variation ID: 37891). In summary, this variant meets criteria to be classified as pathogenic for autosomal dominant HBOC. ACMG/AMP Criteria applied: PVS1, PM2, PS4_Supporting.
13:32912902:AAGA>*	*BRCA2* Frameshift	p.Lys1472ThrfsX6	2	The p.Lys1472ThrfsX6 variant in BRCA2 has been reported in at least 5 individuals with BRCA2-related cancers (Hansen 2017, Li 2018, Pietschmann 2005, Konstantopoulou 2014). It has also been identified in 1/112594 European chromosomes by gnomAD (http://gnomad.broadinstitute.org); however, this frequency is low enough to be consistent with the frequency of hereditary breast and ovarian cancer (HBOC) in the general population. This variant is predicted to cause a frameshift, which alters the protein's amino acid sequence beginning at position 1472 and leads to a premature termination codon 6 amino acids downstream. This alteration is then predicted to lead to a truncated

				or absent protein. Loss of function of the BRCA2 gene is an established disease mechanism in autosomal dominant hereditary breast and ovarian cancer syndrome (HBOC). Additionally, this variant was classified as Pathogenic on Sept 8 2016 by the ClinGen-approved ENIGMA expert panel (Variation ID: 37902). In summary, this variant meets criteria to be classified as pathogenic for autosomal dominant HBOC. ACMG/AMP Criteria applied: PVS1, PM2, PS4_Moderate.
13:32912956:C>*	*BRCA2* Frameshift	p.His1488GlnfsX4	1	The p.His1488GlnfsX4 variant in BRCA2 has not been previously reported in individuals with hereditary breast and/or ovarian cancer (HBOC) and was absent from large population studies. This variant is predicted to cause a frameshift, which alters the protein's amino acid sequence beginning at position 1488 and leads to a premature termination codon 4 amino acids downstream. This alteration is then predicted to lead to a truncated or absent protein. Loss of function of the BRCA2 gene is an established disease mechanism in autosomal dominant HBOC. In summary, although additional studies are required to fully establish its clinical significance, this variant meets criteria to be classified as likely pathogenic for autosomal dominant HBOC. ACMG/AMP Criteria applied: PVS1, PM2.
13:32912965:GAAA>*	*BRCA2* Frameshift	p.Glu1493ValfsX10	5	The p.Glu1493ValfsX10 variant in BRCA2 has been reported in >20 individuals with BRCA2-related cancers and segregated with disease in 12 individuals from one family (Tavtigian 1996, Kote-Jarai 2011, Zhang 2011, Maxwell 2016, Susswein 2016, Shi 2017, AlDubayan 2018, Dudley 2018, Mijuskovic 2018, BIC database). It has also been identified in 0.0035% (4/112994) European chromosomes by gnomAD (http://gnomad.broadinstitute.org); however, this frequency is low enough to be consistent with the frequency of hereditary breast and ovarian cancer (HBOC) in the general population. This variant is predicted to cause a frameshift, which alters the protein's amino acid sequence beginning at position 1493 and leads to a premature termination codon 10 amino acids downstream. This alteration is then predicted to lead to a truncated or absent protein. Loss of function of the BRCA2 gene is an established disease mechanism in autosomal dominant HBOC. Additionally, this variant was classified as Pathogenic on Apr 22 2016 by the ClinGen-approved ENIGMA expert panel (Variation ID: 51653). In summary, this variant meets criteria to be classified as pathogenic for autosomal dominant HBOC. ACMG/AMP Criteria applied: PVS1, PS4, PP1_Strong, PM2.
13:32913119:A>*	*BRCA2* Frameshift	p.Asn1544ThrfsX24	1	The p.Asn1544ThrfsX24 variant in BRCA2 (also referred to in the literature as c.4859delA) is a founder variant in the Phillipines and has been reported in >20 individuals with BRCA2-related cancers (Hopper 1999, Zhang 2011, De Leon Matsuda 2002, BIC database). It has also been identified in 1/34418 Latino chromosomes by gnomAD (http://gnomad.broadinstitute.org); however, this frequency is low enough to be consistent with the frequency of hereditary breast and ovarian cancer (HBOC) in the general population. This variant is predicted to cause a frameshift, which alters the protein's amino acid sequence beginning at position 1544 and leads to a premature termination codon 24 amino acids downstream. This alteration is then predicted to lead to a truncated or absent protein. Loss of function of the BRCA2 gene is an established disease mechanism in autosomal dominant hereditary breast and ovarian cancer syndrome (HBOC). Additionally, this variant was classified as Pathogenic on Sept 8 2016 by the ClinGen-approved ENIGMA expert panel (Variation ID: 37913). In summary, this variant meets criteria to be classified as pathogenic for autosomal dominant HBOC. ACMG/AMP Criteria applied: PVS1, PM2, PS4.
13:32913206:*>GCAAAGAC	*BRCA2* Frameshift	p.Ala1572GlyfsX10	1	The p.Ala1572GlyfsX10 variant in BRCA2 has not been previously reported in individuals with hereditary breast and/or ovarian cancer (HBOC) and was absent from large population studies. This variant is predicted to cause a frameshift, which alters the protein's amino acid sequence beginning at position 1572 and leads to a premature termination codon 10 amino acids downstream. This alteration is then predicted to lead to a truncated or absent protein. Loss of function of the BRCA2 gene is an established disease mechanism in autosomal dominant HBOC. In summary, although additional studies are required to fully establish its clinical significance, this variant meets criteria to be classified as likely pathogenic for autosomal dominant HBOC. ACMG/AMP Criteria applied: PVS1, PM2.
13:32913209:TG>*	*BRCA2* Nonsense	p.Cys1573X	1	The p.Cys1573X variant in BRCA2 has not been previously reported in individuals with hereditary breast and/or ovarian cancer (HBOC) and was absent from large population studies. This nonsense variant leads to a premature termination codon at position 1573, which is predicted to lead to a

				truncated or absent protein. Loss of function of the BRCA2 gene is an established disease mechanism in autosomal dominant HBOC. In summary, although additional studies are required to fully establish its clinical significance, this variant meets criteria to be classified as likely pathogenic for autosomal dominant HBOC. ACMG/AMP Criteria applied: PVS1, PM2.
13:32913221:G>T	*BRCA2* Nonsense	p.Glu1577X	1	The p.Glu1577X variant in BRCA2 has been reported in two individuals with breast cancer (Couch 2015, Copson 2018). It was absent from large population studies. This nonsense variant leads to a premature termination codon at position 1577, which is predicted to lead to a truncated or absent protein. Loss of function of the BRCA2 gene is an established disease mechanism in autosomal dominant hereditary breast and ovarian cancer syndrome (HBOC). In summary, this variant meets criteria to be classified as pathogenic for autosomal dominant HBOC. ACMG/AMP Criteria applied: PVS1, PM2, PS4_Supporting.
13:32913296:A>T	*BRCA2* Nonsense	p.Lys1602X	1	The p.Lys1602X variant in BRCA2 has not been previously reported in individuals with hereditary breast and/or ovarian cancer (HBOC) and was absent from large population studies. This nonsense variant leads to a premature termination codon at position 1602, which is predicted to lead to a truncated or absent protein. Loss of function of the BRCA2 gene is an established disease mechanism in autosomal dominant HBOC. In summary, although additional studies are required to fully establish its clinical significance, this variant meets criteria to be classified as likely pathogenic for autosomal dominant HBOC. ACMG/AMP Criteria applied: PVS1, PM2.
13:32913351:T>G	*BRCA2* Nonsense	p.Leu1620X	1	The p.Leu1620X variant in BRCA2 has been reported in at least 4 individuals with breast or ovarian cancer (Risch 2001, Zhang 2011, BIC database). It has also been identified in 1/113080 European chromosomes by gnomAD (http://gnomad.broadinstitute.org); however, this frequency is low enough to be consistent with the frequency of hereditary breast and ovarian cancer (HBOC) in the general population. This variant leads to a premature termination codon at position 1620, which is predicted to lead to a truncated or absent protein. Loss of function of the BRCA2 gene is an established disease mechanism in autosomal dominant hereditary breast and ovarian cancer syndrome (HBOC). Additionally, this variant was classified as Pathogenic on Sept 8, 2016 by the ClinGen-approved ENIGMA expert panel (Variation ID: 51730). In summary, this variant meets criteria to be classified as pathogenic for autosomal dominant HBOC. ACMG/AMP Criteria applied: PVS1, PM2, PS4_Supporting.
13:32913366:AA>*	*BRCA2* Frameshift	p.Asn1626SerfsX12	2	The p.Asn1626SerfsX12 variant in BRCA2 has been reported in >20 individuals with BRCA2-associated cancers (Risch 2001, Kote-Jarai 2011, Gonzalez-Garay 2013, Leongamornlert 2014, Meric-Bernstam 2016, Breast Cancer Information Core (BIC) database). This variant has also been identified in 2/112998 European chromosomes by the Genome Aggregation Database (GnomAD, http://gnomad.broadinstitute.org); however, this frequency is low enough to be consistent with the frequency of hereditary breast and ovarian cancer (HBOC) in the general population. This variant is predicted to cause a frameshift, which alters the protein's amino acid sequence beginning at position 1626 and leads to a premature termination codon 12 amino acids downstream. This alteration is then predicted to lead to a truncated or absent protein. Heterozygous loss of function of the BRCA2 gene is an established disease mechanism in individuals with hereditary breast and ovarian cancer (HBOC). In addition, this variant was classified as Pathogenic on April 22, 2016 by the ClinGen-approved ENIGMA expert panel (ClinVar SCV000282396.1). In summary, the p.Asn1626fs variant meets our criteria to be classified as pathogenic for HBOC in an autosomal dominant manner. ACMG/AMP criteria applied: PVS1, PS4.
13:32913457:C>A	*BRCA2* Nonsense	p.Tyr1655X	1	The p.Tyr1655X variant in BRCA2 has been reported in at least 8 individuals with BRCA2-associated cancers (Ramus 2007, Bayraktar 2012, Castro 2013, Chong 2014, Tung 2015, Susswein 2016). This variant has also been identified in 2/125700 European chromosomes by the Genome Aggregation Database (GnomAD, http://gnomad.broadinstitute.org); however, this frequency is low enough to be consistent with the frequency of hereditary breast and ovarian cancer (HBOC) in the general population. This nonsense variant leads to a premature termination codon at position 1655, which is predicted to lead to a truncated or absent protein. Heterozygous loss of BRCA2 function is an

				established disease mechanism in hereditary breast and ovarian cancer (HBOC). In summary, this variant meets criteria to be classified as pathogenic for HBOC in an autosomal dominant manner based upon the predicted impact to the protein and low frequency in controls. ACMG/AMP criteria applied: PVS1, PS4_Moderate, PM2_Supporting.
13:32913457:C>G	*BRCA2* Nonsense	p.Tyr1655X	1	The p.Tyr1655X variant in BRCA2 has been reported in at least 8 individuals with BRCA2-associated cancers (Ramus 2007, Bayraktar 2012, Castro 2013, Chong 2014, Tung 2015, Susswein 2016). This variant has also been identified in 2/125700 European chromosomes by the Genome Aggregation Database (GnomAD, http://gnomad.broadinstitute.org); however, this frequency is low enough to be consistent with the frequency of hereditary breast and ovarian cancer (HBOC) in the general population. This nonsense variant leads to a premature termination codon at position 1655, which is predicted to lead to a truncated or absent protein. Heterozygous loss of BRCA2 function is an established disease mechanism in hereditary breast and ovarian cancer (HBOC). In summary, this variant meets criteria to be classified as pathogenic for HBOC in an autosomal dominant manner based upon the predicted impact to the protein and low frequency in controls. ACMG/AMP criteria applied: PVS1, PS4_Moderate, PM2_Supporting.
13:32913558:*>A	*BRCA2* Frameshift	p.Trp1692MetfsX3	11	The p.Trp1692MetfsX3 variant in BRCA2 has been identified in >30 individuals of various ethnicities with Fanconi anemia or BRCA2-related cancers (Risch 2001, Offit 2003, Van Der Hout 2006, Laarabi 2011, Breast Cancer Information Core database). It has also been identified in 1/8884 Ashkenazi Jewish chromosomes by the Genome Aggregation Consortium (GnomAD, http://gnomad.broadinstitute.org). This frequency is low enough to be consistent with the frequency of hereditary breast and ovarian cancer (HBOC) in the general population. This variant is predicted to cause a frameshift, which alters the protein's amino acid sequence beginning at position 1692 and leads to a premature termination codon 3 amino acids downstream. This alteration is then predicted to lead to a truncated or absent protein. Heterozygous loss of function of the BRCA2 gene is an established disease mechanism in HBOC. In addition, this variant was classified as Pathogenic on April 22, 2016 by the ClinGen-approved ENIGMA expert panel (ClinVar SCV000282397.1). In summary, this variant meets our criteria to be classified as pathogenic for HBOC in an autosomal dominant manner. ACMG/AMP criteria applied: PVS1, PS4, PM2.
13:32913620:TATG>*	*BRCA2* Nonsense	p.Tyr1710X	1	The p.Tyr1710X variant in BRCA2 (resulting from c.5130_5133delTGTA) has been reported in >15 individuals with BRCA2-related cancers (Friedman 1997, de Juan Jiménez 2013, Maxwell 2017, Na 2017, Wong-Brown 2015, Soumittra 2009, Pritzlaff 2017, BIC database). It has also been identified in 1/15268 African chromosomes by gnomAD (http://gnomad.broadinstitute.org); however, this frequency is low enough to be consistent with the frequency of hereditary breast and ovarian cancer (HBOC) in the general population. This frameshift variant is predicted to lead to a premature termination codon at position 1710, which is predicted to lead to a truncated or absent protein. Loss of function of the BRCA2 gene is an established disease mechanism in autosomal dominant hereditary breast and ovarian cancer syndrome (HBOC). Additionally, this variant was classified as Pathogenic on Apr 22, 2016 by the ClinGen-approved ENIGMA expert panel (Variation ID: 51775). In summary, this variant meets criteria to be classified as pathogenic for autosomal dominant HBOC. ACMG/AMP Criteria applied: PVS1, PM2, PS4.
13:32913778:TC>*	*BRCA2* Frameshift	p.Ser1764LysfsX3	2	The p.Ser1764LysfsX3 variant in BRCA2 has been reported in at least 5 individuals with breast or ovarian cancer (Reedy 2002, de Juan Jiménez 2013, Cunningham 2014, Song 2014, BIC database). It has also been identified in 1/113118 European chromosomes by gnomAD (http://gnomad.broadinstitute.org); however, this frequency is low enough to be consistent with the frequency of hereditary breast and ovarian cancer (HBOC) in the general population. This variant is predicted to cause a frameshift, which alters the protein's amino acid sequence beginning at position 1764 and leads to a premature termination codon 3 amino acids downstream. This alteration is then predicted to lead to a truncated or absent protein. Loss of function of the BRCA2 gene is an established disease mechanism in autosomal dominant hereditary breast and ovarian cancer syndrome (HBOC). Additionally, this variant was classified as Pathogenic on Sept 8 2016 by the

				ClinGen-approved ENIGMA expert panel (Variation ID: 37956). In summary, this variant meets criteria to be classified as pathogenic for autosomal dominant HBOC. ACMG/AMP Criteria applied: PVS1, PM2, PS4_Moderate.
13:32913795:TT>*	*BRCA2* Frameshift	p.Leu1768ArgfsX5	5	The p.Leu1768ArgfsX5 variant in BRCA2 has been reported in at least 15 individuals with BRCA2-related cancers and segregated with disease in at least 1 individual from one family (Gayther 2000, Gutiérrez Espeleta 2012, Shindo 2017, Tea 2014, Alsop 2012, Lecarpentier 2012, Castro 2013, Kraus 2017, Mitra 2008, BIC database). It has also been identified in 1/113142 European chromosomes by gnomAD (http://gnomad.broadinstitute.org); however, this frequency is low enough to be consistent with the frequency of hereditary breast and ovarian cancer (HBOC) in the general population. This variant is predicted to cause a frameshift, which alters the protein's amino acid sequence beginning at position 1768 and leads to a premature termination codon 5 amino acids downstream. This alteration is then predicted to lead to a truncated or absent protein. Loss of function of the BRCA2 gene is an established disease mechanism in autosomal dominant hereditary breast and ovarian cancer syndrome (HBOC). Additionally, this variant was classified as Pathogenic on Sept 8 2016 by the ClinGen-approved ENIGMA expert panel (Variation ID: 37957). In summary, this variant meets criteria to be classified as pathogenic for autosomal dominant HBOC. ACMG/AMP Criteria applied: PVS1, PM2, PS4.
13:32913837:AA>*	*BRCA2* Frameshift	p.Asn1784HisfsX2	5	The p.Asn1784HisfsX2 variant in BRCA2 has been identified in >30 individuals with BRCA2-associated cancers (Gayther 1997, Walsh 2011, Zhang 2011, George 2013, Cunningham 2014, Breast Cancer Information Core (BIC) database, Sharing Clinical Reports Project). This variant has been identified in 1/15316 of European chromosomes by the Genome Aggregation Database (GnomAD, http://gnomad.broadinstitute.org); however, this frequency is low enough to be consistent with the frequency of hereditary breast and ovarian cancer (HBOC) in the general population. This variant is predicted to cause a frameshift, which alters the protein's amino acid sequence beginning at position 1784 and leads to a premature termination codon 2 amino acids downstream. This alteration is then predicted to lead to a truncated or absent protein. Heterozygous loss of function of the BRCA2 gene is an established disease mechanism in individuals with hereditary breast and ovarian cancer (HBOC). In summary, this variant meets our criteria to be classified as pathogenic for HBOC in an autosomal dominant manner based upon the predicted impact to the protein. ACMG/AMP criteria applied: PVS1, PS4, PM2.
13:32913857:A>T	*BRCA2* Nonsense	p.Lys1789X	1	The p.Lys1789X variant in BRCA2 has not been previously reported in individuals with hereditary breast and/or ovarian cancer (HBOC) and was absent from large population studies, but has been reported in ClinVar (Variation ID: 266875). This nonsense variant leads to a premature termination codon at position 1789, which is predicted to lead to a truncated or absent protein. Loss of function of the BRCA2 gene is an established disease mechanism in autosomal dominant HBOC. In summary, although additional studies are required to fully establish its clinical significance, this variant meets criteria to be classified as likely pathogenic for autosomal dominant HBOC. ACMG/AMP Criteria applied: PVS1, PM2.
13:32914066:AATT>*	*BRCA2* Frameshift	p.Ile1859LysfsX3	2	The p.Ile1859LysfsX3 variant in BRCA2 has been reported in >35 individuals with BRCA2-associated cancers (Saghir 2015, Kim 2016, Breast Cancer Information Core database). It has also been identified in 1/15994 African chromosomes by the Genome Aggregation Database (GnomAD, http://gnomad.broadinstitute.org); however, this frequency is low enough to be consistent with the frequency of hereditary breast and ovarian cancer (HBOC) in the general population. This variant is predicted to cause a frameshift, which alters the protein's amino acid sequence beginning at position 1859 and leads to a premature termination codon 3 amino acids downstream. This alteration is then predicted to lead to a truncated or absent protein. Heterozygous loss of function of the BRCA2 gene is an established disease mechanism in HBOC. In addition, this variant was classified as Pathogenic on April 22, 2016 by the ClinGen-approved ENIGMA expert panel (ClinVar SCV000282408.1). In summary, this variant meets our criteria to be classified as pathogenic for HBOC in an autosomal dominant manner. ACMG/AMP criteria applied: PVS1, PS4, PM2.

13:32914174:C>G	*BRCA2* Nonsense	p.Tyr1894X	2	The p.Tyr1894X (c.5682C>G) variant in BRCA2 has been reported in >20 individuals with BRCA2-related cancers (Risch 2001, Edwards 2010, Tea 2014, Alemar 2017, AlDubayan 2018, Dudley 2018, BIC database). Furthermore, another variant at this position resulting in the same amino acid change (c.5681dup, p.Tyr1894X) is classified as pathogenic for hereditary breast and ovarian cancer (HBOC) by our laboratory. The c.5682C>G variant has also been identified in 0.001% (1/113148) of European chromosomes by gnomAD (http://gnomad.broadinstitute.org); however, this frequency is low enough to be consistent with the frequency of HBOC in the general population. This nonsense variant is predicted to lead to a premature termination codon at position 1894, which is predicted to lead to a truncated or absent protein. Loss of function of the BRCA2 gene is an established disease mechanism in HBOC. Finally, the p.Tyr1894X (c.5682C>G) variant was classified as Pathogenic on Apr 22, 2016 by the ClinGen-approved ENIGMA expert panel (Variation ID: 37989). In summary, this variant meets criteria to be classified as pathogenic for autosomal dominant HBOC. ACMG/AMP Criteria applied: PVS1, PM2, PS4, PS1.
13:32914210:CT>*	*BRCA2* Frameshift	p.Leu1908ArgfsX2	2	The p.Leu1908ArgfsX2 variant in BRCA2 has been reported in >40 individuals with BRCA2-associated cancers (Kwong 2009, Cherbal 2010, Zhang 2011, Edwards 2010, Breast Cancer Information Core (BIC) database). It has also been identified in 1/30598 South Asian chromosomes by the Genome Aggregation Database (GnomAD, http://gnomad.broadinstitute.org); however, this frequency is low enough to be consistent with the frequency of hereditary breast and ovarian cancer (HBOC) in the general population. This variant is predicted to cause a frameshift, which alters the protein's amino acid sequence beginning at position 1908 and leads to a premature termination codon 2 amino acids downstream. This alteration is then predicted to lead to a truncated or absent protein. Heterozygous loss of function of the BRCA2 gene is an established disease mechanism in hereditary breast and ovarian cancer (HBOC). In summary, this variant meets criteria to be classified as pathogenic for HBOC in an autosomal dominant manner. ACMG/AMP criteria applied: PVS1, PS4, PM2.
13:32914401:C>A	*BRCA2* Nonsense	p.Ser1970X	2	The p.Ser1970X (c.5682C>A) variant in BRCA2 has been reported in at least 12 individuals with BRCA2-related cancers (Gayther 1997, Edwards 2010, Leongamornlert 2014, Labidi-Galy 2018, BIC database) and was absent from large population studies. This nonsense variant is predicted to lead to a premature termination codon at position 1970, which is predicted to lead to a truncated or absent protein. Loss of function of the BRCA2 gene is an established disease mechanism in autosomal dominant hereditary breast and ovarian cancer syndrome (HBOC). Additionally, this variant was classified as Pathogenic on Apr 22, 2016 by the ClinGen-approved ENIGMA expert panel (Variation ID: 38007). In summary, this variant meets criteria to be classified as pathogenic for autosomal dominant HBOC. ACMG/AMP Criteria applied: PVS1, PM2, PS4_Moderate.
13:32914438:T>*	*BRCA2* Frameshift	p.Ser1982ArgfsX22	4	The p.Ser1982ArgfsX22 variant in BRCA2 is a founder mutation in the Ashkenazi Jewish population (Finkelman 2012) and has been identified in >500 individuals of various ethnicities with BRCA2-associated cancers (Breast Cancer Information Core (BIC) database: https://research.nhgri.nih.gov/projects/bic/). It has also been identified in 0.6% (59/10151) Ashkenazi Jewish chromosomes and 10/126512 European chromosomes by the Genome Aggregation Database (gnomAD, http://gnomad.broadinstitute.org; dbSNP rs80359550). This variant is predicted to cause a frameshift, which alters the protein's amino acid sequence beginning at position 1982 and leads to a premature termination codon 22 amino acids downstream. This alteration is then predicted to lead to a truncated or absent protein. Heterozygous loss of function of the BRCA2 gene is an established disease mechanism in individuals with hereditary breast and ovarian cancer (HBOC). Additionally, this variant was classified as pathogenic on April 22, 2016 by the ClinGen-approved ENIGMA expert panel (ClinVar SCV000282418.1). In summary, the p.Ser1982fs variant meets criteria to be classified as pathogenic for HBOC in an autosomal dominant manner based upon the predicted impact to the protein and presence in multiple affected individuals. ACMG/AMP Criteria applied: PVS1, PS4_Strong.

13:32914452:A>*	*BRCA2* Frameshift	p.Gln1987ArgfsX17	1	The p.Gln1987ArgfsX17 variant in BRCA2 has not been previously reported in individuals with hereditary breast and/or ovarian cancer (HBOC) and was absent from large population studies. This variant is predicted to cause a frameshift, which alters the protein's amino acid sequence beginning at position 1987 and leads to a premature termination codon 17 amino acids downstream. This alteration is then predicted to lead to a truncated or absent protein. Loss of function of the BRCA2 gene is an established disease mechanism in autosomal dominant HBOC. In summary, although additional studies are required to fully establish its clinical significance, this variant meets criteria to be classified as likely pathogenic for autosomal dominant HBOC. ACMG/AMP Criteria applied: PVS1, PM2.
13:32914551:AACA>*	*BRCA2* Frameshift	p.Glu2020ValfsX19	3	The p.Glu2020ValfsX19 variant in BRCA2 has been reported in at least 6 families with breast cancer (Heramb 2018, Bergman 2005, Grindedal 2017). It was absent from large population studies. This variant is predicted to cause a frameshift, which alters the protein's amino acid sequence beginning at position 2020 and leads to a premature termination codon 19 amino acids downstream. This alteration is then predicted to lead to a truncated or absent protein. Loss of function of the BRCA2 gene is an established disease mechanism in autosomal dominant hereditary breast and ovarian cancer syndrome (HBOC). Additionally, this variant was classified as Pathogenic on Sept 8, 2016 by the ClinGen-approved ENIGMA expert panel (Variation ID: 91435). In summary, this variant meets criteria to be classified as pathogenic for autosomal dominant HBOC. ACMG/AMP Criteria applied: PVS1, PM2, PS4_Moderate.
13:32914767:TT>*	*BRCA2* Frameshift	p.Leu2092ProfsX7	4	The p.Leu2092ProfsX7 variant in BRCA2 has been reported >100 individuals with BRCA2-associated cancer (Breast Information Core Database (BIC)); Bayraktar 2012, de Juan 2015, Edwards 2010, Fostira 2018, Mijuskovic 2018, Walsh 2011, Wang 2018, Whitworth 2018, Wooster 1995, Zhang 2011). In addition, the p.Leu2080X variant was classified as Pathogenic on Apr 22, 2016 by the ClinGen-approved ENIGMA expert panel (ClinVar SCV000282422.1) and has been suggested to be a European founder mutation (Janavicius 2010). This variant has been identified in 0.005% (6/124234) of European chromosomes by the Genome Aggregation Database (gnomAD, http://gnomad.broadinstitute.org. It is predicted to cause a frameshift, which alters the protein's amino acid sequence beginning at position 2092 and leads to a premature termination codon 7 amino acids downstream. This alteration is then predicted to lead to a truncated or absent protein. Heterozygous loss of function of the BRCA2 gene is an established disease mechanism in hereditary breast and ovarian cancer (HBOC). In summary, this variant meets criteria to be classified as pathogenic for HBOC in an autosomal dominant manner. ACMG/AMP Criteria applied: PVS1, PS4, PM2.
13:32914894:TAACT>*	*BRCA2* Frameshift	p.Asn2135LysfsX3	3	The p.Asn2135LysfsX3 variant in BRCA2 has been reported in at least 12 individuals with BRCA2-associated cancers (Gayther 1997, Wagner 1999, Risch 2001, Gomes 2007, Machackova 2008, Kote-Jarai 2011, Zhang 2011, de Juan 2015, Hirotsu 2015, Breast Cancer Information Core database, www.research.nhgri.nih.gov/bic/). This variant has also been identified in 1/11488 of Latino chromosomes by the Exome Aggregation Consortium (ExAC, http://exac.broadinstitute.org; dbSNP rs80359585). This frequency is low enough to be consistent with the frequency of hereditary breast and ovarian cancer (HBOC) in the general population. This variant is predicted to cause a frameshift, which alters the protein's amino acid sequence beginning at position 2135 and leads to a premature termination codon 3 amino acids downstream. This alteration is then predicted to lead to a truncated or absent protein. Heterozygous loss of function of the BRCA2 gene is an established disease mechanism in HBOC. In addition, this variant was classified as Pathogenic on April 22, 2016 by the ClinGen-approved ENIGMA expert panel (ClinVar SCV000282424.1). In summary, this variant meets our criteria to be classified as pathogenic for HBOC in an autosomal dominant manner based upon the predicted impact to the protein. ACMG/AMP Criteria applied: PVS1, PS4_Moderate, PM2.
13:32914954:TC>*	*BRCA2* Frameshift	p.Gln2157IlefsX18	1	The p.Gln2157IlefsX18 variant in BRCA2 has been reported in >30 individuals with BRCA2-associated cancers (Vietri 2012, Ghiorzo 2012, Manoukian 2007, Gao 2000, Veschi 2009, Papi 2007, Breast Cancer Information Core (BIC) database) and was absent from large population studies. This variant is predicted to cause a frameshift, which alters the protein's amino acid sequence beginning at position 2157 and leads to a premature termination codon 18 amino acids downstream. This alteration

				is then predicted to lead to a truncated or absent protein. Heterozygous loss of function of the BRCA2 gene is an established disease mechanism in individuals with hereditary breast and ovarian cancer (HBOC). In addition, this variant was classified as Pathogenic on April 22, 2016 by the ClinGen-approved ENIGMA expert panel (ClinVar SCV000282426.1). In summary, this variant meets criteria to be classified as pathogenic for HBOC in an autosomal dominant manner based upon the predicted impact to the protein. ACMG/AMP Criteria applied: PVS1, PS4, PM2.
13:32914974:ACAA>*	*BRCA2* Frameshift	p.Lys2162AsnfsX5	2	The p.Lys2162AsnfsX5 variant in BRCA2 has been reported in greater than 30 individuals with BRCA2-associated cancer (de Juan Jimenez 2013, Edwards 2003, Edwards 2010, Labidi-Galy 2018, Li 2018, Nielsen 2016, Sun 2017; Breast Information Core database). It has been identified in 1/26026 South Asian and 1/29656 Latino chromosomes by gnomAD (http://gnomad.broadinstitute.org). This variant was classified as Pathogenic on April 22, 2016 by the ClinGen-approved ENIGMA expert panel (Variation ID 38048). This variant is predicted to cause a frameshift, which alters the protein's amino acid sequence beginning at position 2162 and leads to a premature termination codon 5 amino acids downstream. This alteration is then predicted to lead to a truncated or absent protein. Loss of function of the BRCA2 gene is an established disease mechanism in autosomal dominant hereditary breast and ovarian cancer syndrome. In summary, this variant meets criteria to be classified as pathogenic for autosomal dominant hereditary breast and ovarian cancer syndrome. ACMG/AMP Criteria applied: PVS1, PS4, PM2.
13:32915044:G>*	*BRCA2* Frameshift	p.Ala2185LeufsX6	1	The p.Ala2185LeufsX6 variant in BRCA2 has been reported in at least 8 individuals with breast cancer (Kim 2012, Park 2017, Sun 2017, Li 2018, BIC database). It was absent from large population studies. This variant is predicted to cause a frameshift, which alters the protein's amino acid sequence beginning at position 2185 and leads to a premature termination codon 6 amino acids downstream. This alteration is then predicted to lead to a truncated or absent protein. Loss of function of the BRCA2 gene is an established disease mechanism in autosomal dominant hereditary breast and ovarian cancer syndrome (HBOC). Additionally, this variant was classified as Pathogenic on Sept 8, 2016 by the ClinGen-approved ENIGMA expert panel (Variation ID: 52127). In summary, this variant meets criteria to be classified as pathogenic for autosomal dominant HBOC. ACMG/AMP Criteria applied: PVS1, PM2, PS4_Moderate.
13:32915078:AA>*	*BRCA2* Frameshift	p.Lys2196AsnfsX2	1	The p.Lys2196AsnfsX2 variant in BRCA2 has not been previously reported in individuals with hereditary breast and/or ovarian cancer (HBOC) and was absent from large population studies, but has been reported in ClinVar (Variation ID: 236280). This variant is predicted to cause a frameshift, which alters the protein's amino acid sequence beginning at position 2196 and leads to a premature termination codon 2 amino acids downstream. This alteration is then predicted to lead to a truncated or absent protein. Loss of function of the BRCA2 gene is an established disease mechanism in autosomal dominant HBOC. In summary, although additional studies are required to fully establish its clinical significance, this variant meets criteria to be classified as likely pathogenic for autosomal dominant HBOC. ACMG/AMP Criteria applied: PVS1, PM2.
13:32915094:C>*	*BRCA2* Frameshift	p.Ser2201LeufsX5	1	The p.Ser2201LeufsX5 variant in BRCA2 has not been previously reported in individuals with hereditary breast and/or ovarian cancer (HBOC) and was absent from large population studies, but has been reported in ClinVar (Variation ID: 96839). This variant is predicted to cause a frameshift, which alters the protein's amino acid sequence beginning at position 2201 and leads to a premature termination codon 5 amino acids downstream. This alteration is then predicted to lead to a truncated or absent protein. Loss of function of the BRCA2 gene is an established disease mechanism in autosomal dominant HBOC. In summary, although additional studies are required to fully establish its clinical significance, this variant meets criteria to be classified as likely pathogenic for autosomal dominant HBOC. ACMG/AMP Criteria applied: PVS1, PM2.
13:32920968:AATA>*	*BRCA2* Frameshift	p.Ile2315LysfsX12	1	The p.Ile2315LysfsX12 variant in BRCA2 has been reported in at least 15 families with breast and/or ovarian cancer (Frank 1998, Wong-Brown 2015, Evans 2008, Teer 2016, BIC database). It has also been identified in 2/113008 European chromosomes by gnomAD (http://gnomad.broadinstitute.org); however, this frequency is low enough to be consistent with the frequency of hereditary breast and

				ovarian cancer (HBOC) in the general population. This variant is predicted to cause a frameshift, which alters the protein's amino acid sequence beginning at position 2315 and leads to a premature termination codon 12 amino acids downstream. This alteration is then predicted to lead to a truncated or absent protein. Loss of function of the BRCA2 gene is an established disease mechanism in autosomal dominant hereditary breast and ovarian cancer syndrome (HBOC). Additionally, this variant was classified as Pathogenic on Sept 8, 2016 by the ClinGen-approved ENIGMA expert panel (Variation ID: 91435). In summary, this variant meets criteria to be classified as pathogenic for autosomal dominant HBOC. ACMG/AMP Criteria applied: PVS1, PM2, PS4_Strong.
13:32921004:T>*	*BRCA2* Nonsense	p.Leu2327X	5	The p.Leu2327X variant in BRCA2 has not been previously reported in individuals with hereditary breast and/or ovarian cancer (HBOC) and was absent from large population studies, but has been reported in ClinVar (Variation ID: 252419). This nonsense variant leads to a premature termination codon at position 2327, which is predicted to lead to a truncated or absent protein. Loss of function of the BRCA2 gene is an established disease mechanism in autosomal dominant HBOC. In summary, although additional studies are required to fully establish its clinical significance, this variant meets criteria to be classified as likely pathogenic for autosomal dominant HBOC. ACMG/AMP Criteria applied: PVS1, PM2.
13:32929058:TC>*	*BRCA2* Frameshift	p.Leu2357ValfsX2	6	The p.Leu2357ValfsX2 variant in BRCA2 has been identified in >30 individuals with BRCA2-associated cancers (Garvin 1997, Spearman 2008, Borg 2010, Caux-Moncoutier 2011, Zhang 2011, Breast Cancer Information Core (BIC) database). This variant has also been identified in 7/12892 European chromosomes by the Genome Aggregation Database (GnomAD, http://gnomad.broadinstitute.org); however, this frequency is low enough to be consistent with the frequency of hereditary breast and ovarian cancer (HBOC) in the general population. This variant is predicted to cause a frameshift, which alters the protein's amino acid sequence beginning at position 2357 and leads to a premature termination codon 2 amino acids downstream. This alteration is then predicted to lead to a truncated or absent protein. Heterozygous loss of function of the BRCA2 gene is an established disease mechanism in individuals with HBOC. In addition, this variant was classified as Pathogenic on April 22, 2016 by the ClinGen-approved ENIGMA expert panel (ClinVar SCV000282439.1). In summary, this variant meets criteria to be classified as pathogenic for HBOC in an autosomal dominant manner based upon the predicted impact to the protein. ACMG/AMP Criteria applied: PVS1, PS4, PM2.
13:32929208:TG>*	*BRCA2* Frameshift	p.Val2407SerfsX4	1	The p.Val2407SerfsX4 variant in BRCA2 has not been previously reported in individuals with hereditary breast and/or ovarian cancer (HBOC) and was absent from large population studies. This variant is predicted to cause a frameshift, which alters the protein's amino acid sequence beginning at position 2407 and leads to a premature termination codon 4 amino acids downstream. This alteration is then predicted to lead to a truncated or absent protein. Loss of function of the BRCA2 gene is an established disease mechanism in autosomal dominant HBOC. In summary, although additional studies are required to fully establish its clinical significance, this variant meets criteria to be classified as likely pathogenic for autosomal dominant HBOC. ACMG/AMP Criteria applied: PVS1, PM2.
13:32929350:A>*	*BRCA2* Frameshift	p.Ile2454PhefsX13	1	The p.Ile2454PhefsX13 variant in BRCA2 has been reported in 2 individuals with breast cancer (BIC database) and was absent from large population studies. This variant is predicted to cause a frameshift, which alters the protein's amino acid sequence beginning at position 2454 and leads to a premature termination codon 13 amino acids downstream. This alteration is then predicted to lead to a truncated or absent protein. Loss of function of the BRCA2 gene is an established disease mechanism in autosomal dominant HBOC. Additionally, this variant was classified as Pathogenic on Apr 22, 2016 by the ClinGen-approved ENIGMA expert panel (Variation ID: 52312). In summary, this variant meets criteria to be classified as pathogenic for autosomal dominant HBOC. ACMG/AMP Criteria applied: PVS1, PM2, PS4_Supporting.
13:32930609:C>T	*BRCA2* Nonsense	p.Arg2494X	2	The p.Arg2494X variant in BRCA2 has been reported in >20 individuals with BRCA2-related cancers and has been reported as a Finnish founder variant (Vehmanen 1997, Park 2016, Eoh 2017, Park 2017, Sun 2017, Weren 2017, Labidi-Galy 2018, Lee 2018, BIC database). It has also been identified

				in 4/21648 Finnish chromosomes by gnomAD (http://gnomad.broadinstitute.org); however, this frequency is low enough to be consistent with the frequency of hereditary breast and ovarian cancer (HBOC) in the general population. This variant leads to a premature termination codon at position 2494, which is predicted to lead to a truncated or absent protein. Loss of function of the BRCA2 gene is an established disease mechanism in autosomal dominant HBOC. Additionally, this variant was classified as Pathogenic on Apr 22, 2016 by the ClinGen-approved ENIGMA expert panel (Variation ID: 38099). In summary, this variant meets criteria to be classified as pathogenic for autosomal dominant HBOC. ACMG/AMP Criteria applied: PVS1, PS4.
13:32930687:C>T	*BRCA2* Nonsense	p.Arg2520X	1	The p.Arg2520X variant in BRCA2 has been reported in >40 individuals with BRCA2-associated cancers (Hàkansson 1997, Bayraktar 2012, Castéra 2014, Schultheis 2014, Breast Cancer Information Core (BIC) database), and segregated with associated cancers in 2 affected relatives from 1 family. This variant has also been identified in 3/113554 European chromosomes by the Genome Aggregation Database (GnomAD, http://gnomad.broadinstitute.org); however, this frequency is low enough to be consistent with the frequency of hereditary breast and ovarian cancer (HBOC) in the general population This nonsense variant leads to a premature termination codon at position 2520, which is predicted to lead to a truncated or absent protein. Heterozygous loss of BRCA2 function is an established disease mechanism in hereditary breast and ovarian cancer (HBOC). In summary, this variant meets criteria to be classified as pathogenic for HBOC in an autosomal dominant manner based on the low frequency in controls, presence in affected individuals, and predicted impact to the protein. ACMG/AMP Criteria applied: PVS1, PS4, PM2_Supporting.
13:32930706:CAGT>*	*BRCA2* Frameshift	p.Ala2526GlufsX24	1	The p.Ala2526GlufsX24 variant in BRCA2 has not been previously reported in individuals with hereditary breast and/or ovarian cancer (HBOC) and was absent from large population studies. This variant is predicted to cause a frameshift, which alters the protein's amino acid sequence beginning at position 2526 and leads to a premature termination codon 24 amino acids downstream. This alteration is then predicted to lead to a truncated or absent protein. Loss of function of the BRCA2 gene is an established disease mechanism in autosomal dominant HBOC. In summary, although additional studies are required to fully establish its clinical significance, this variant meets criteria to be classified as likely pathogenic for autosomal dominant HBOC. ACMG/AMP Criteria applied: PVS1, PM2.
13:32930711:*>A	*BRCA2* Frameshift	p.Gly2528GlufsX11	1	The p.Gly2528GlufsX11 variant in BRCA2 has not been previously reported in individuals with hereditary breast and/or ovarian cancer (HBOC) and was absent from large population studies. This variant is predicted to cause a frameshift, which alters the protein's amino acid sequence beginning at position 2528 and leads to a premature termination codon 11 amino acids downstream. This alteration is then predicted to lead to a truncated or absent protein. Loss of function of the BRCA2 gene is an established disease mechanism in autosomal dominant HBOC. In summary, although additional studies are required to fully establish its clinical significance, this variant meets criteria to be classified as likely pathogenic for autosomal dominant HBOC. ACMG/AMP Criteria applied: PVS1, PM2.
13:32932018:G>A	*BRCA2* Nonsense	p.Trp2586X	1	The p.Trp2586X variant in BRCA2 has been reported in at least 14 individuals with BRCA2-related cancer (Perkowska 2003, Willems-Jones 2012, George 2013, Li 2018, BIC database). It has also been identified in 2/113726 European chromosomes by gnomAD (http://gnomad.broadinstitute.org); however, this frequency is low enough to be consistent with the frequency of hereditary breast and ovarian cancer (HBOC) in the general population. This variant leads to a premature termination codon at position 2586, which is predicted to lead to a truncated or absent protein. Loss of function of the BRCA2 gene is an established disease mechanism in autosomal dominant hereditary breast and ovarian cancer syndrome (HBOC). Another variant, c.7758G>A, resulting in the same amino acid change has been identified in individuals with BRCA2-related cancers and is classified as pathogenic by this laboratory. Additionally, this variant was classified as Pathogenic on Apr 22, 2016 by the ClinGen-approved ENIGMA expert panel (Variation ID: 52401). In summary, this variant meets criteria to be classified as pathogenic for autosomal dominant HBOC. ACMG/AMP Criteria applied: PVS1, PM2, PS4_Moderate, PS1.

13:32936737:*>A	*BRCA2* Frameshift	p.Trp2629MetfsX12	1	The p.Trp2629MetfsX12 variant in BRCA2 has not been previously reported in individuals with hereditary breast and/or ovarian cancer (HBOC) and was absent from large population studies, but has been reported in ClinVar (Variation ID: 52431). This variant is predicted to cause a frameshift, which alters the protein's amino acid sequence beginning at position 2629 and leads to a premature termination codon 12 amino acids downstream. This alteration is then predicted to lead to a truncated or absent protein. Loss of function of the BRCA2 gene is an established disease mechanism in autosomal dominant HBOC. In summary, although additional studies are required to fully establish its clinical significance, this variant meets criteria to be classified as likely pathogenic for autosomal dominant HBOC. ACMG/AMP Criteria applied: PVS1, PM2.
13:32936812:T>C	*BRCA2* Missense	p.Leu2653Pro	1	The p.Leu2653Pro variant in BRCA2 has been reported in at least 3 individuals with breast cancer (Easton 2007, BIC database) and was absent from large population studies, but has been reported in Clinvar (Variation ID: 52447). In vitro assays provide some evidence that this variant impacts protein function (Biswas 2012, Guidugli 2014, Bernards 2016, Guidugli 2018, Mesman 2018); however, these types of assays may not accurately represent biological function. Computational prediction tools and conservation analyses suggest that this variant may impact the protein, though this information is not predictive enough to determine pathogenicity. In summary, although additional studies are required to fully establish its clinical significance, this variant meets criteria to be classified as likely pathogenic for autosomal dominant hereditary breast and ovarian cancer syndrome (HBOC). ACMG/AMP Criteria applied: PM2, PS3_Moderate, PP3, PS4_Supporting.
13:32937327:A>T	*BRCA2* Missense	p.Glu2663Val	1	The p.Glu2663Val variant in BRCA2 has been reported in at least 9 individuals with BRCA2-associated cancers (Breast Cancer Information Core database: https://research.nhgri.nih.gov/projects/bic/, Szabo 2000, Chevenix-Trench 2006, Borg 2010, Akbari 2011), and was absent from large population studies. In vitro functional studies suggest that this variant may alter protein function (Kuznetsov 2008, Farrugia 2008, Sanz 2010, Walker 2010, Whiley 2014, Fraile-Bethencourt 2017). Computational prediction tools and conservation analysis also suggest that the p.Glu2663Val variant may impact the protein. In addition, this variant was classified as pathogenic on August 10, 2015 by the ClinGen-approved ENIGMA expert panel (ClinVar SCV000244478.1). In summary, although additional studies are required to fully establish its clinical significance, the p.Glu2663Val variant is likely pathogenic. ACMG/AMP Criteria applied (Richards 2015): PM2, PS3_Supporting, PS4_Moderate, PP3.
13:32937507:A>C	*BRCA2* Missense	p.Asp2723Ala	2	The p.Asp2723Ala variant in BRCA2 has been reported in at least 4 individuals with breast caner (Scott 2003, Gorringe 2008, BIC database). It has also been identified in 1/113396 European chromosomes by gnomAD (http://gnomad.broadinstitute.org); however, this frequency is low enough to be consistent with the frequency of hereditary breast and ovarian cancer (HBOC) in the general population. This variant has also been reported in Clinvar (Variation ID: 52516). Computational prediction tools and conservation analyseis suggest that this variant may impact the protein, though this information is not predictive enough to determine pathogenicity. In vitro assays provide some evidence that this variant impact protein function (Guidugli 2018); however, these types of assays may not accurately represent biological function. Another variant involving this codon (p.Asp2723His) has been identified in individuals with breast cancer and has been classified as Pathogenic by the ClinGen-approved ENIGMA expert panel. In summary, although additional studies are required to fully establish its clinical significance, this variant meets criteria to be classified as likely pathogenic for autosomal dominant hereditary breast and ovarian cancer syndrome (HBOC). ACMG/AMP Criteria applied: PM2, PS3_Moderate, PM5, PP3, PS4_Supporting.
13:32937636:C>*	*BRCA2* Frameshift	p.Thr2766AsnfsX11	2	The p.Thr2766AsnfsX11 (NM_000059.3 c.8297delC) variant in BRCA2 (also referred to as c.8285delC in the literature) has been previously reported in many individuals and families with breast, ovarian or prostate cancer (Tavtigian 1996, Castro 2013, McVeigh 2014, Wong-Brown 2015), and was absent from large population studies. In addition, this variant was classified as Pathogenic by the ClinGen-approved ENIGMA expert panel (ClinVar SCV000282457.1). This variant is predicted to cause a frameshift, which alters the protein's amino acid sequence beginning at position 2766 and

				leads to a premature termination codon 11 amino acids downstream. This alteration is then predicted to lead to a truncated or absent protein. Heterozygous loss of BRCA2 function is an established disease mechanism in hereditary breast and ovarian cancer syndrome (HBOC). In summary, this variant meets our criteria to be classified as pathogenic for HBOC in an autosomal dominant manner based on its occurrence in affected individuals and its predicted impact to the protein. ACMG/AMP Criteria applied: PVS1, PM2, PS4_Moderate, PP5 (Richards 2015).
13:32937672:T>A	*BRCA2*<br>splice site	c.8331+2T>A	1	The c.8331+2T>A variant in BRCA2 has not been previously reported in individuals with hereditary breast and/or ovarian cancer (HBOC) and was absent from large population studies, but has been reported in ClinVar (Variation ID: 91507). This variant occurs within the canonical splice site (+/- 1,2) and is predicted to cause altered splicing leading to an abnormal or absent protein. Loss of function of the BRCA2 gene is an established disease mechanism in autosomal dominant HBOC. In summary, although additional studies are required to fully establish its clinical significance, this variant meets criteria to be classified as likely pathogenic for autosomal dominant HBOC. ACMG/AMP Criteria applied: PVS1, PM2.
13:32937672:T>C	*BRCA2*<br>splice site	c.8331+2T>C	1	The c.8331+2T>C variant in BRCA2 has been reported in at least 2 probands with BRCA2-related cancer (Cunningham 2014, Tung 2015). It was absent from large population studies, but has been reported in ClinVar (Variation ID: 267692). This variant occurs within the canonical splice site (+/- 1,2) and is predicted to cause altered splicing leading to an abnormal or absent protein. In vitro functional studies confirm that this variant leads to abnormal splicing (Fraile-Bethencourt 2017). In summary, this variant meets criteria to be classified as pathogenic for autosomal dominant hereditary breast and ovarian cancer (HBOC). ACMG/AMP Criteria applied: PVS1, PM2, PS4_Supporting.
13:32944692:C>T	*BRCA2*<br>Nonsense	p.Gln2829X	2	The p.Gln2829X variant in BRCA2 has been reported in at least 6 individuals with hereditary breast and/or ovarian cancer (HBOC; Yang 2015, Shi 2017, Sun 2017, Wang 2018, BIC database). It was absent from large population studies. This nonsense variant leads to a premature termination codon at position 2829, which is predicted to lead to a truncated or absent protein. Loss of function of the BRCA2 gene is an established disease mechanism in autosomal dominant hereditary breast and ovarian cancer syndrome (HBOC). Additionally, this variant was classified as Pathogenic on Dec 15, 2017 by the ClinGen-approved ENIGMA expert panel (Variation ID: 52600). In summary, this variant meets criteria to be classified as pathogenic for autosomal dominant HBOC. ACMG/AMP Criteria applied: PVS1, PM2, PS4_Moderate.
13:32945092:G>A	*BRCA2*<br>splice site	c.8488-1G>A	1	The c.8488-1G>A variant in BRCA2 has been reported in at least 8 probands with BRCA2-related cancers, as well as in one proband with Fanconi anemia who had this variant in the homozygous state (Acedo 2012, Santos 2014, Park 2017, Li 2018 , Palmero 2018, Cotrim 2019). It was absent from large population studies, but has been reported in ClinVar (Variation ID: 38164). This variant occurs within the canonical splice site (+/- 1,2) and is predicted to cause altered splicing leading to an abnormal or absent protein. Sequencing of patient RNA confirms that this variant leads to abnormal splicing (Howlett 2002, Acedo 2012, Santos 2014). In summary, this variant meets criteria to be classified as pathogenic for autosomal dominant hereditary breast and ovarian cancer (HBOC). ACMG/AMP Criteria applied: PVS1, PM2, PS4_Moderate.
13:32945180:C>*	*BRCA2*<br>Frameshift	p.Gln2859LysfsX4	3	The p.Gln2859LysfsX4 variant in BRCA2 has been reported in >20 individuals with BRCA2-related cancers (Martin 2001, Pritchard 2016, Roed Nielsen 2016, BIC database). It was absent from large population databases. This variant is predicted to cause a frameshift, which alters the protein's amino acid sequence beginning at position 2859 and leads to a premature termination codon 4 amino acids downstream. This alteration is then predicted to lead to a truncated or absent protein. Loss of function of the BRCA2 gene is an established disease mechanism in autosomal dominant hereditary breast and ovarian cancer syndrome (HBOC). Additionally, this variant was classified as Pathogenic on Apr 22, 2016 by the ClinGen-approved ENIGMA expert panel (Variation ID: 38169). In summary, this variant meets criteria to be classified as pathogenic for autosomal dominant HBOC. ACMG/AMP Criteria applied: PVS1, PM2, PS4.

13:32953602:C>*	*BRCA2* Frameshift	p.Val2969CysfsX7	2	The p.Val2969CysfsX7 variant in BRCA2 has been reported at least 40 individuals with BRCA2-associated cancers (Claes 2004, Kote-Jarai 2011, Frank 1998, Breast Cancer Information Core (BIC) database), and was absent from large population studies. This variant is predicted to cause a frameshift, which alters the protein's amino acid sequence beginning at position 2969 and leads to a premature termination codon 7 amino acids downstream. This alteration is then predicted to lead to a truncated or absent protein. Heterozygous loss of function of the BRCA2 gene is an established disease mechanism in hereditary breast and ovarian cancer (HBOC). In addition, this variant was classified as Pathogenic on April 22, 2016 by the ClinGen-approved ENIGMA expert panel (ClinVar SCV000282464.1). In summary, the p.Val2969CysfsX7 variant meets criteria to be classified as pathogenic for HBOC in an autosomal dominant manner based upon the predicted impact to the protein. ACMG/AMP Criteria applied: PVS1, PS4, PM2.
13:32953632:C>A	*BRCA2* Nonsense	p.Ser2978X	1	The p.Ser2978X variant (resulting from c.8933C>A) in BRCA2 has been reported in at least 3 individuals with breast cancer (Scott 2003, BIC database). It was absent from large population studies. This variant leads to a premature termination codon at position 2978, which is predicted to lead to a truncated or absent protein. Loss of function of the BRCA2 gene is an established disease mechanism in autosomal dominant hereditary breast and ovarian cancer syndrome (HBOC). Another variant at this position that leads to the same amino acid change (c.8933C>G) has also been reported in one proband with breast cancer (Momozawa 2018). This variant was classified as Pathogenic on Apr 22, 2016 by the ClinGen-approved ENIGMA expert panel (Variation ID: 52704). In summary, this variant meets criteria to be classified as pathogenic for autosomal dominant HBOC. ACMG/AMP Criteria applied: PVS1, PM2, PS4_Supporting, PS1.
13:32953886:G>A	*BRCA2* splice site	c.8954-1G>A	3	The c.8954-1G>A variant in BRCA2 has not been previously reported in individuals with hereditary breast and/or ovarian cancer (HBOC) and was absent from large population studies, but has been reported in ClinVar (Variation ID: 531281). This variant occurs within the canonical splice site (+/- 1,2) and is predicted to cause altered splicing leading to an abnormal or absent protein. Loss of function of the BRCA2 gene is an established disease mechanism in autosomal dominant HBOC. In summary, although additional studies are required to fully establish its clinical significance, this variant meets criteria to be classified as likely pathogenic for autosomal dominant HBOC. ACMG/AMP Criteria applied: PVS1, PM2.
13:32953987:TA>*	*BRCA2* Frameshift	p.Ser3018ArgfsX3	1	The p.Ser3018ArgfsX3 variant in BRCA2 has been reported in 2 individuals with breast cancer (BIC database). It has also been identified in 1/113052 European chromosomes by gnomAD (http://gnomad.broadinstitute.org); however, this frequency is low enough to be consistent with the frequency of hereditary breast and ovarian cancer (HBOC) in the general population. This variant is predicted to cause a frameshift, which alters the protein's amino acid sequence beginning at position 3018 and leads to a premature termination codon 3 amino acids downstream. This alteration is then predicted to lead to a truncated or absent protein. Loss of function of the BRCA2 gene is an established disease mechanism in autosomal dominant hereditary breast and ovarian cancer syndrome (HBOC). Additionally, this variant was classified as Pathogenic on Sep 8, 2016 by the ClinGen-approved ENIGMA expert panel (Variation ID: 52312). In summary, this variant meets criteria to be classified as pathogenic for autosomal dominant HBOC. ACMG/AMP Criteria applied: PVS1, PM2, PS4_Supporting.
13:32954006:AT>*	*BRCA2* Frameshift	p.Ile3025ThrfsX18	1	The p.Ile3025ThrfsX18 variant in BRCA2 has been reported in 1 family with breast and/or ovarian cancer (Marroni 2004). It was absent from large population studies. This variant has also been reported in ClinVar (Variation ID: 52741). This variant is predicted to cause a frameshift, which alters the protein's amino acid sequence beginning at position 3025 and leads to a premature termination codon 18 amino acids downstream. This alteration is then predicted to lead to a truncated or absent protein. Loss of function of the BRCA2 gene is an established disease mechanism in autosomal dominant hereditary breast and ovarian cancer syndrome (HBOC). In summary, although additional studies are required to fully establish its clinical significance, this variant meets criteria to be classified as likely pathogenic for autosomal dominant HBOC. ACMG/AMP Criteria applied: PVS1, PM2.

13:32954050:G>A	*BRCA2* splice site	p.Pro3039Pro	1	The p.Pro3039Pro (c.9117G>A) variant in BRCA2 has been reported in at least 16 individuals with BRCA2-related cancer (Peelen 2000, Houdayer 2012, Willems-Jones 2012, de Juan 2015, Corman 2016, Labidi-Galy 2018, BIC database). It has also been identified in 1/111660 European chromosomes by gnomAD (http://gnomad.broadinstitute.org); however, this frequency is low enough to be consistent with the frequency of hereditary breast and ovarian cancer (HBOC) in the general population. This variant has also been identified in ClinVar (Variation ID: 38215). This variant is located in the last base of the exon, which is part of the 5' splice region. Computational tools predict a splicing impact, and both in vitro studies and testing of patient RNA have shown that this variant results in exon skipping, which is predicted to lead to an absent or truncated protein (Peelen 2000, Acedo 2012, Houdayer 2012, Colombo 2013). Loss of function of the BRCA2 gene is an established disease mechanism in autosomal dominant hereditary breast and ovarian cancer syndrome (HBOC). Another variant, c.9117G>T, resulting in the same synonymous change and predicted splicing impact has also been identified in individuals with BRCA2-related cancers. In summary, this variant meets criteria to be classified as pathogenic for autosomal dominant HBOC. ACMG/AMP Criteria applied: PS3, PM2, PS4.
13:32968835:*>T	*BRCA2* Frameshift	p.Val3091ArgfsX20	1	The p.Val3091ArgfsX20 variant in BRCA2 has been reported in 1 individual with breast cancer (BIC database) and was absent from large population studies. This variant is predicted to cause a frameshift, which alters the protein's amino acid sequence beginning at position 3091 and leads to a premature termination codon 20 amino acids downstream. This alteration is then predicted to lead to a truncated or absent protein. Loss of function of the BRCA2 gene is an established disease mechanism in autosomal dominant HBOC. Additionally, this variant was classified as Pathogenic on Apr 22, 2016 by the ClinGen-approved ENIGMA expert panel (Variation ID: 52797). In summary, although additional studies are required to fully establish its clinical significance, this variant meets criteria to be classified as likely pathogenic for autosomal dominant HBOC. ACMG/AMP Criteria applied: PVS1, PM2.
13:32968847:T>A	*BRCA2* Nonsense	p.Leu3093X	1	The p.Leu3093X variant in BRCA2 has not been previously reported in individuals with hereditary breast and/or ovarian cancer (HBOC) and was absent from large population studies. This nonsense variant leads to a premature termination codon at position 3093, which is predicted to lead to a truncated or absent protein. Loss of function of the BRCA2 gene is an established disease mechanism in autosomal dominant HBOC. In summary, although additional studies are required to fully establish its clinical significance, this variant meets criteria to be classified as likely pathogenic for autosomal dominant HBOC. ACMG/AMP Criteria applied: PVS1, PM2.
13:32968863:C>G	*BRCA2* Nonsense	p.Tyr3098X	2	The p.Tyr3098X (c.9294C>A) variant in BRCA2 has been reported in at least 15 individuals with BRCA2-related cancers (Frank 1998, S/NI consortium 2003, Kerr 2016, Sun 2017, Bannon 2018, Wen 2018, BIC database). It has also been identified in 2/24950 African chromosomes by gnomAD (http://gnomad.broadinstitute.org); however, this frequency is low enough to be consistent with the frequency of hereditary breast and ovarian cancer (HBOC) in the general population. This variant leads to a premature termination codon at position 3098, which is predicted to lead to a truncated or absent protein. Loss of function of the BRCA2 gene is an established disease mechanism in autosomal dominant HBOC. This variant was classified as Pathogenic on Apr 22, 2016 by the ClinGen-approved ENIGMA expert panel (Variation ID: 38229). In summary, this variant meets criteria to be classified as pathogenic for autosomal dominant HBOC. ACMG/AMP Criteria applied: PVS1, PS4
13:32968968:A>*	*BRCA2* Frameshift	p.Gly3134AlafsX29	1	The p.Gly3134AlafsX29 variant in BRCA2 has not been previously reported in individuals with hereditary breast and/or ovarian cancer (HBOC) and was absent from large population studies. This variant is predicted to cause a frameshift, which alters the protein's amino acid sequence beginning at position 3134 and leads to a premature termination codon 29 amino acids downstream. This alteration is then predicted to lead to a truncated or absent protein. Loss of function of the BRCA2 gene is an established disease mechanism in autosomal dominant HBOC. In summary, although additional

				studies are required to fully establish its clinical significance, this variant meets criteria to be classified as likely pathogenic for autosomal dominant HBOC. ACMG/AMP Criteria applied: PVS1, PM2.
13:32969001:TG>*	*BRCA2* Frameshift	p.Ser3147CysfsX2	1	The p.Ser3147CysfsX2 variant in BRCA2 has been reported in >20 individuals with breast cancer (Cunningham 2014, Phelan 1996, BIC database). It has also been identified in 1/113672 European chromosomes by gnomAD (http://gnomad.broadinstitute.org); however, this frequency is low enough to be consistent with the frequency of hereditary breast and ovarian cancer (HBOC) in the general population. This variant is predicted to cause a frameshift, which alters the protein's amino acid sequence beginning at position 3147 and leads to a premature termination codon 2 amino acids downstream. This alteration is then predicted to lead to a truncated or absent protein. Loss of function of the BRCA2 gene is an established disease mechanism in autosomal dominant hereditary breast and ovarian cancer syndrome (HBOC). Additionally, this variant was classified as Pathogenic on Sep 8, 2016 by the ClinGen-approved ENIGMA expert panel (Variation ID: 38240). In summary, this variant meets criteria to be classified as pathogenic for autosomal dominant HBOC. ACMG/AMP Criteria applied: PVS1, PM2, PS4.
17:41197784:G>A	*BRCA1* Nonsense	p.Arg1835X	3	The p.Arg1835X variant in BRCA1 has been identified in >50 individuals with BRCA1-associated cancers (Breast Cancer Information Core (BIC) database). This variant has been identified in 2/30616 South Asian chromosomes by the Genome Aggregation Database (GnomAD, http://gnomad.broadinstitute.org); however, this frequency is low enough to be consistent with the frequency of hereditary breast and ovarian cancer (HBOC) in the general population. This nonsense variant leads to a premature termination codon at position 1835. This alteration occurs within the last exon and is more likely to escape nonsense mediated decay (NMD), resulting in a truncated protein. However, in vitro functional studies provide some evidence that this truncation may impact protein function (Ye 2001). Furthermore, this variant was classified as Pathogenic on Apr 22, 2016 by the ClinGen-approved ENIGMA Expert Panel (ClinVar SCV000282345.1). In summary, this variant meets criteria to be classified as pathogenic for autosomal dominant hereditary breast and ovarian cancer (HBOC) based upon its frequency in affected individuals and absence from controls. ACMG/AMP Criteria applied: PVS1_Strong, PS4, PS3_Supporting, PM2.
17:41201209:G>A	*BRCA1* Nonsense	p.Gln1779X	1	The p.Gln1779X variant in BRCA1 has been reported in 2 siblings with early-onset breast cancer (Levanat 2012). It was absent from large population studies. This variant leads to a premature termination codon at position 1779, which is predicted to lead to a truncated or absent protein. Loss of function of the BRCA1 gene is an established disease mechanism in autosomal dominant hereditary breast and ovarian cancer syndrome (HBOC). In vitro functional studies support an impact on protein function (Findlay 2018). This variant was classified as Pathogenic on Oct 18, 2016 by the ClinGen-approved ENIGMA expert panel (Variation ID: 55540). In summary, this variant meets criteria to be classified as pathogenic for autosomal dominant HBOC. ACMG/AMP Criteria applied: PVS1, PM2, PS3_Moderate.
17:41203088:A>C	*BRCA1* Missense	p.Met1775Arg	1	The p.Met1775Arg variant in BRCA2 has been reported in >20 individuals with BRCA1-related cancers, with a higher prevalence in those of African descent (Futreal 1994, Pal 2015, Hall 2009, Miki 1994, Fackenthal 2012, BIC database) and has been classified in ClinVar as Pathogenic on Aug 10, 2015 by the ClinGen-approved ENIGMA expert panel (Variation ID: 17694). It has also been identified in 4/24950 African chromosomes by gnomAD (http://gnomad.broadinstitute.org); however, this frequency is low enough to be consistent with the frequency of hereditary breast and ovarian cancer (HBOC) in the general population. Computational prediction tools and conservation analysis suggest that the p.Met1775Arg variant may impact the protein, though this information is not predictive enough to determine pathogenicity. In vitro functional studies support an impact on protein function (Kawai 2002, Caligo 2009, Lee 2010, Findlay 2018). In summary, this variant meets criteria to be classified as pathogenic for autosomal dominant HBOC. ACMG/AMP Criteria applied: PS3, PS4, PP1_Moderate, PM2_Supporting, PP3.
17:41209079:*>G	*BRCA1* Frameshift	p.Gln1756ProfsX74	1	The p.Gln1756ProfsX74 variant in BRCA1 (also referred to as p.Gln1777fs) is a founder variant in the Ashkenazi Jewish population and has been reported in >1000 individuals with BRCA1-associated

				cancers (Abeliovich 1997, Elwad 2011, Breast Cancer Information Core (BIC) database). This variant has also been identified in 24/103702 Ashkenazi Jewish and 25/129200 European chromosomes by the Genome Aggregation Database (gnomAD, http://gnomad.broadinstitute.org; dbSNP rs397507247). This variant is predicted to cause a frameshift, which alters the protein's amino acid sequence beginning at position 1756 and leads to a premature termination codon 74 amino acids downstream. This alteration is then predicted to lead to a truncated or absent protein. Heterozygous loss of function of the BRCA1 gene is an established disease mechanism in hereditary breast and ovarian cancer (HBOC). Furthermore, the p.Gln1756fs variant was classified as Pathogenic on April 22, 2016 by the ClinGen-approved ENIGMA expert panel (ClinVar SCV000282341.1). In summary, this variant meets criteria to be classified as pathogenic for HBOC in an autosomal dominant manner based upon the predicted impact to the protein and presence in affected individuals. ACMG/AMP Criteria applied: PS4, PVS1.
17:41209139:A>G	*BRCA1* Missense	p.Val1736Ala	1	The p.Val1736Ala variant in BRCA1 has been identified in at least 5 individuals with BRCA1-associated cancer and segregated with disease in 4 affected relatives, including 1 obligate carrier (Akbari 2011, Domchek 2013, Finch 2016, Thompson 2016). One of the probands with ovarian cancer, short stature, and developmental delay also carried a loss-of-function variant in BRCA1 in trans (Domchek 2013). This variant was absent from large population studies but has been reported in ClinVar (Variation ID# 37648). Computational prediction tools and conservation analysis suggest that this variant may impact the protein. In addition, the majority of in vitro functional studies support a loss-of-function impact on protein function (Carvalho 2007, Lee 2010, Rowling 2010, Domchek 2013, Gaboriau 2015, Woods 2016, Findlay 2018). In summary, although additional studies are required to fully establish its clinical significance, this variant meets criteria to be classified as likely pathogenic for autosomal dominant hereditary breast and ovarian cancer. ACMG/AMP Criteria applied: PM2, PS3_Moderate, PP1, PP3, PS4_Supporting.
17:41215348:A>*	*BRCA1* splice site	c.5193+2delT	2	The c.5193+2delT variant in BRCA1 has been reported in at least 7 families with hereditary breast and/or ovarian cancer (HBOC) and segregated with disease in at least 3 affected individuals from 1 family (Wagner 1999, Claes 2003, Gayther 1995, BIC database). It was absent from large population studies. This variant has also been reported in ClinVar (Variation ID: 55450). This variant occurs within the canonical splice site (+/- 1,2) and is predicted to cause altered splicing leading to an abnormal or absent protein. In vitro functional studies on patient cells show that this variant leads to exon 19 skipping, leading to a premature stop codon (Houdayer 2012, Claes 2003). This is expected to lead to a truncated or absent protein and loss of function of the BRCA1 gene is an established disease mechanism in autosomal dominant HBOC. In summary, this variant meets criteria to be classified as pathogenic for autosomal dominant HBOC. ACMG/AMP Criteria applied: PVS1, PM2, PS3_Moderate, PS4_Moderate, PP1.
17:41215890:C>A	*BRCA1* splice site	c.5152+1G>T	1	The c.5152+1G>T variant in BRCA1 has been reported in at least 11 probands hereditary breast and/or ovarian cancer (HBOC; Gayther 1995, Brovkina 2018, BIC database). It was absent from large population studies. This variant occurs within the canonical splice site (+/- 1,2) and is predicted to cause altered splicing leading to an abnormal or absent protein. Loss of function of the BRCA1 gene is an established disease mechanism in autosomal dominant HBOC. In vitro functional studies support an impact on protein function (Findlay 2018). This variant was classified as Pathogenic on Oct 18, 2016 by the ClinGen-approved ENIGMA expert panel (Variation ID: 55423). In summary, this variant meets criteria to be classified as pathogenic for autosomal dominant HBOC. ACMG/AMP Criteria applied: PVS1, PM2, PS3_Moderate, PS4_Moderate.
17:41215902:A>C	*BRCA1* Missense	p.Val1714Gly	1	The p.Val1714Gly variant in BRCA1 has been reported in at least 3 individuals with hereditary breast and/or ovarian cancer (HBOC) and segregated with disease in 4 affected individuals from one family (Li 2018, Zhang 2015, BIC database). It was absent from large population studies. This variant has also been reported in ClinVar (Variation ID: 55413). Computational prediction tools and conservation analyses suggest that this variant may impact the protein, though this information is not predictive enough to determine pathogenicity. In vitro functional studies provide some evidence that this variant

				impacts protein function (Lee 2010, Findlay 2018, Woods 2016); however, these types of assays may not accurately represent biological function. In summary, although additional studies are required to fully establish its clinical significance, this variant meets criteria to be classified as likely pathogenic. ACMG/AMP Criteria applied: PM2, PS3_Moderate, PP1, PP3, PS4_Supporting.
17:41215920:G>T	*BRCA1* Missense	p.Ala1708Glu	1	The p.Ala1708Glu variant in BRCA1 has been reported in more than 40 individuals with hereditary breast and ovarian cancer (HBOC) and segregated with disease in at least 5 affected relatives from 3 families (Futreal 1994, Greenman 1998, Blesa 2000, de la Hoya 2002, Infante 2006, Torres 2007, Laitman 2011, Sagi 2011, Laitman 2012, Rodriguez 2012, de Juan 2013, Hernandez 2014). It has also been identified in 2/34580 Latino chromosomes by the Genome Aggregation Database (gnomAD, http://gnomad.broadinstitute.org); however, this frequency is low enough to be consistent with the frequency of HBOC in the general population. Computational prediction tools and conservation analysis suggest that this variant may impact the protein. In vitro functional studies provide some evidence that the p.Ala1729Glu variant may cause skipping of exon 18 (Millevoi 2010, Sanz 2010). In addition, this variant was classified as Pathogenic on Aug 10, 2015 by the ClinGen-approved ENIGMA Expert Panel (ClinVar SCV000244385.1). In summary, the p.Ala1729Glu variant meets criteria to be classified as pathogenic for autosomal dominant HBOC. ACMG/AMP Criteria applied: PS4, PM2, PS3_Moderate, PP1_Moderate.
17:41215947:C>T	*BRCA1* Missense	p.Arg1699Gln	3	The p.Arg1699Gln variant in BRCA1 has been reported in >60 individuals with BRCA1-associated cancers and segregated with disease in multiple relatives from 30 families (Spurdle 2012, Shimelis 2017, Moghadasi 2018). This variant has been described as having reduced penetrance compared to other disease-causing variants: up to 24% risk of BRCA1-related cancer by age 70 (95% CI, 10% to 40%) for Arg1699Gln carriers vs. 58% (95% CI, 7% to 72%) for Arg1699Trp carriers vs. 4.6% risk for women in the general population (Spurdle 2012, Moghadasi 2018). It has been identified in 6/113618 European chromosomes by the Genome Aggregation Database (gnomAD, http://gnomad.broadinstitute.org). While some studies have demonstrated impaired in vitro protein activity, others report that the variant performed similar to wild-type (Williams 2003, Lovelock 2007, Chang 2011). Computational prediction tools and conservation analysis suggest that this variant may impact the protein. In summary, this variant meets criteria to be classified as a low-penetrant pathogenic variant for autosomal dominant HBOC. ACMG/AMP criteria applied: PS4, PP1_Strong, PM5, PM2_Supporting, PP3, PS3_Supporting.
17:41215948:G>A	*BRCA1* Missense	p.Arg1699Trp	2	The p.Arg1699Trp variant in BRCA1 is an established pathogenic variant that has been identified in multiple individuals of various ethnic backgrounds with BRCA1-associated cancer and segregated with disease in multiple families (Vallon-Christersson 2001, Rhiem 2007, Kuusisto 2011, Zhang 2011, Spurdle 2012, Larqui 2013, Song 2014, Zahra 2016, Alemar 2017, Barrios 2017, Hirasawa 2017, Alhuqail 2018, Rebbeck 2018, Bhaskaran 2019, Concolino 2019). It was also identified as a de novo change in 1 individual with early onset breast cancer (paternity confirmed; Antonucci 2017) and in the compound heterozygous state with a loss-of-function BRCA1 variant in an individual with breast cancer, short stature, intellectual disability, and multiple congenital anomalies (Sawyer 2015). Multiple in vitro analyses as well as multifactorial probability models are consistent with pathogenicity (Carvalho 2007, Easton 2007, Lee 2010, Coquelle 2011, Spurdle 2012, Bouwman 2013). This variant is present in 6/251242 chromosomes by gnomAD (https://gnomad.broadinstitute.org) and was classified as pathogenic in ClinVar by several laboratories and the ClinGen-approved ENIGMA expert panel (Variation ID 55396). In summary, this variant meets criteria to be classified as pathogenic for autosomal dominant hereditary breast and ovarian cancer. ACMG/AMP Criteria applied: PS2, PS4, PP1_Strong, PM2_Supporting, PS3_Moderate, PP3.
17:41226397:AG>*	*BRCA1* Frameshift	p.Ser1542TrpfsX31	1	The p.Ser1542TrpfsX31 variant in BRCA1 has been reported in at least 6 individuals with hereditary breast and/or ovarian cancer (HBOC; Evans 2003, BIC database) and was absent from large population studies. This variant is predicted to cause a frameshift, which alters the protein's amino acid sequence beginning at position 1542 and leads to a premature termination codon 31 amino acids downstream. This alteration is then predicted to lead to a truncated or absent protein. Loss of function

				of the BRCA1 gene is an established disease mechanism in autosomal dominant HBOC. Additionally, this variant was classified as Pathogenic on Sep 8, 2016 by the ClinGen-approved ENIGMA expert panel (Variation ID: 55243). In summary, this variant meets criteria to be classified as pathogenic for autosomal dominant HBOC. ACMG/AMP Criteria applied: PVS1, PM2, PS4_Moderate.
17:41226448:TT>*	*BRCA1* Frameshift	p.Gln1525ArgfsX5	1	The p.Gln1525ArgfsX5 variant in BRCA1 has been reported in at least 15 individuals with hereditary breast and/or ovarian cancer (HBOC; Greenman 1998, Ellis 2000, Al-Mulla 2009, Song 2014, Robertson 2012, BIC database) and was absent from large population studies. This variant is predicted to cause a frameshift, which alters the protein's amino acid sequence beginning at position 1525 and leads to a premature termination codon 5 amino acids downstream. This alteration is then predicted to lead to a truncated or absent protein. Loss of function of the BRCA1 gene is an established disease mechanism in autosomal dominant HBOC. Additionally, this variant was classified as Pathogenic on Apr 22, 2016 by the ClinGen-approved ENIGMA expert panel (Variation ID: 55229). In summary, this variant meets criteria to be classified as pathogenic for autosomal dominant HBOC. ACMG/AMP Criteria applied: PVS1, PM2, PS4.
17:41226499:C>T	*BRCA1* Nonsense	p.Trp1508X	1	The p.Trp1508X variant in BRCA1 has been reported in >13 individuals with BRCA1-related cancers (Loman 2001, Laitman 2011, Walsh 2011, Bayraktar 2012, Lowery 2018, BIC database). It was absent from large population studies. This nonsense variant leads to a premature termination codon at position 1508, which is predicted to lead to a truncated or absent protein. Loss of function of the BRCA1 gene is an established disease mechanism in autosomal dominant hereditary breast and ovarian cancer syndrome (HBOC). Additionally, this variant was classified as Pathogenic on Sept 8, 2016 by the ClinGen-approved ENIGMA expert panel (Variation ID: 55221). In summary, this variant meets criteria to be classified as pathogenic for autosomal dominant HBOC. ACMG/AMP Criteria applied: PVS1, PM2, PS4_Moderate.
17:41228590:G>*	*BRCA1* Frameshift	p.Gln1467ArgfsX38	1	The p.Gln1467ArgfsX38 variant in BRCA1 has not been previously reported in individuals with hereditary breast and/or ovarian cancer (HBOC) and was absent from large population studies. This variant is predicted to cause a frameshift, which alters the protein's amino acid sequence beginning at position 1467 and leads to a premature termination codon 38 amino acids downstream. This alteration is then predicted to lead to a truncated or absent protein. Loss of function of the BRCA1 gene is an established disease mechanism in autosomal dominant HBOC. In summary, although additional studies are required to fully establish its clinical significance, this variant meets criteria to be classified as likely pathogenic for autosomal dominant HBOC. ACMG/AMP Criteria applied: PVS1, PM2.
17:41234451:G>A	*BRCA1* Nonsense	p.Arg1443X	1	The p.Arg1443X variant in BRCA1 has been previously reported in >100 individuals with BRCA1-associated cancers (Vézina 2005, Hall 2009). It has also been identified in 3/35428 Latino chromosomes by the Genome Aggregation Database (GnomAD, http://gnomad.broadinstitute.org). This nonsense variant leads to a premature termination codon at position 1443, which is predicted to lead to a truncated or absent protein. In addition, functional studies provide some evidence that this variant results in a truncated protein (Caligo 2009). Heterozygous loss of function of the BRCA1 gene is an established disease mechanism in individuals with hereditary breast and ovarian cancer (HBOC). In addition, this variant was classified as Pathogenic on April 22, 2016 by the ClinGen-approved ENIGMA expert panel (ClinVar SCV000282327.1). In summary, this variant meets our criteria to be classified as pathogenic for HBOC in an autosomal dominant manner. ACMG/AMP Criteria applied: PVS1, PS3_Supporting, PS4.
17:41243480:TTGA>*	*BRCA1* Frameshift	p.Asn1355LysfsX10	5	The p.Asn1355LysfsX10 variant in BRCA1 has been reported in at least 17 individuals with hereditary breast and ovarian cancer syndrome (Friedman 1994, Zhang 2011, George 2013, Cao 2013, Cunningham 2014, Leongamornlert 2014, Rashid 2016, Sun 2017, Maxwell 2017, Hirasawa 2017, Li 2018, Singh 2018, Wen 2018, Li 2019Friedman 1994, Zhang 2011, George 2013, Cao 2013, Cunningham 2014, Rashid 2016, Sun 2017, Maxwell 2017, Hirasawa 2017, Li 2018, Singh 2018, Wen 2018, Li 2019) and in 1 individual with prostate cancer (Leongamornlert 2014). It has also been identified in 0.003% (1/30478) of South Asian chromosomes by gnomAD (http://gnomad.broadinstitute.org). This variant was classified as Pathogenic on April 22, 2016 by the

				ClinGen-approved Evidence-based Network for the Interpretation of Germline Mutant Alleles (ENIGMA) expert panel (Variation ID 17674). This variant is predicted to cause a frameshift, which alters the protein’s amino acid sequence beginning at position 1355 and leads to a premature termination codon 10 amino acids downstream. This alteration is then predicted to lead to a truncated or absent protein. Loss of function of the BRCA1 gene is an established disease mechanism in autosomal dominant hereditary breast and ovarian cancer. In summary, this variant meets criteria to be classified as pathogenic for autosomal dominant hereditary breast and ovarian cancer. ACMG/AMP Criteria applied: PVS1, PS4, PM2.
17:41243788:*>A	*BRCA1* Nonsense	p.Lys1254X	1	The p.Lys1254X variant (resulting from c.3759dupT) in BRCA1 has been reported in >20 individuals with breast or ovarian cancer (Greenman 1998, van Orsouw 1999, de Juan Jimenez 2013, Alvarez 2017, BIC database). It was absent from large population studies. This frameshift variant leads to a premature termination codon at position 1254, which is predicted to lead to a truncated or absent protein. Loss of function of the BRCA1 gene is an established disease mechanism in autosomal dominant hereditary breast and ovarian cancer syndrome (HBOC). Additionally, this variant was classified as Pathogenic on Sept 8, 2016 by the ClinGen-approved ENIGMA expert panel (Variation ID: 54992). In summary, this variant meets criteria to be classified as pathogenic for autosomal dominant HBOC. ACMG/AMP Criteria applied: PVS1, PM2, PS4.
17:41243789:AGAC>*	*BRCA1* Frameshift	p.Ser1253ArgfsX10	2	The p.Ser12353ArgfsX10 variant in BRCA1 has been reported in >100 individuals with breast and/or ovarian cancer (HBOC; George 2013, Ghiorzo 2012, Meindl 2002, Pohlreich 2005, Sun 2017, Susswein 2015, Zhang 2011, Breast Cancer Information Core (BIC): https://research.nhgri.nih.gov/bic/). This variant has also been identified in 0.004% (5/113572) of European chromosomes by gnomAD (http://gnomad.broadinstitute.org). This variant is predicted to cause a frameshift, which alters the protein’s amino acid sequence beginning at position 1253 and leads to a premature termination codon 10 amino acids downstream. This alteration is then predicted to lead to a truncated or absent protein. Heterozygous loss of function of the BRCA1 gene is an established disease mechanism in individuals with HBOC. Moreover, this variant was classified as pathogenic on April 22, 2016 by the ClinGen-approved ENIGMA expert panel (ClinVar SCV000282317.1). In summary, this variant meets criteria to be classified as pathogenic for HBOC in an autosomal dominant manner based upon predicted impact to the protein, presence in multiple affected individuals and low frequency in the general population. ACMG/AMP Criteria applied: PVS1, PM2, PS4.
17:41243920:*>T	*BRCA1* Frameshift	p.Glu1210ArgfsX9	1	The p.Glu1210ArgfsX9 variant in BRCA1 has been reported in >10 individuals with breast and/or ovarian cancer (HBOC; Kim 2006, George 2013, Hirasawa 2017, Li 2018, BIC database). It has also been identified in 2/18370 East Asian chromosomes by gnomAD (http://gnomad.broadinstitute.org); however, this frequency is low enough to be consistent with the frequency of hereditary breast and ovarian cancer in the general population. This variant is predicted to cause a frameshift, which alters the protein’s amino acid sequence beginning at position 1210 and leads to a premature termination codon 9 amino acids downstream. This alteration is then predicted to lead to a truncated or absent protein. Loss of function of the BRCA1 gene is an established disease mechanism in autosomal dominant hereditary breast and ovarian cancer syndrome. Additionally, this variant was classified as Pathogenic on Apr 22, 2016 by the ClinGen-approved ENIGMA expert panel (Variation ID: 37534). In summary, this variant meets criteria to be classified as pathogenic for autosomal dominant HBOC. ACMG/AMP Criteria applied: PVS1, PM2, PS4_M.
17:41243941:G>A	*BRCA1* Nonsense	p.Arg1203X	1	The p.Arg1203X variant in BRCA1 has been reported in >40 individuals with BRCA1-associated cancers (Friedman 1994, Manguoglu 2003, Walsh 2011, Solano 2012, Kim 2012, Juwle 2012, Couch 2015, Breast Cancer Information Core (BIC) database, Sharing Clinical Reports Project). This variant has been identified in 1/17194 East Asian and 2/111402 European chromosomes by the Genome Aggregation Database (gnomAD, http://gnomad.broadinstitute.org; dbSNP rs62625308). This nonsense variant leads to a premature termination codon at position 1203, which is predicted to lead to a truncated or absent protein. Heterozygous loss of function of the BRCA1 gene is an established

				disease mechanism in individuals with hereditary breast and ovarian cancer (HBOC). In addition, this variant was classified as Pathogenic on April 22, 2016 by the ClinGen-approved ENIGMA expert panel (ClinVar SCV000282311.1). In summary, this variant meets criteria to be classified as pathogenic for HBOC in an autosomal dominant manner. ACMG/AMP Criteria applied: PVS1, PM2, PS4.
17:41244148:C>A	*BRCA1* Nonsense	p.Glu1134X	2	The p.Glu1134X variant in BRCA1 has been reported in >20 individuals with BRCA1-associated cancers (Wagner 1999, Bergthorsson 2001, Nedelcu 2002, Pal 2005, Couch 2015, Rebbeck 2016, Breast Cancer Information Core (BIC) database: https://research.nhgri.nih.gov/bic/) and was absent from large population studies. This nonsense variant leads to a premature termination codon at position 1134, which is predicted to lead to a truncated or absent protein. Heterozygous loss of function of the BRCA1 gene is an established disease mechanism in individuals with hereditary breast and ovarian cancer (HBOC). In addition, this variant was classified as Pathogenic on April 22, 2016 by the ClinGen-approved ENIGMA expert panel (ClinVar SCV000282306.1). In summary, this variant meets criteria to be classified as pathogenic for HBOC in an autosomal dominant manner based upon presence in multiple affected individuals, absence in the general population and predicted impact to the protein. ACMG/AMP Criteria applied: PVS1; PS4; PM2 (Richards 2015).
17:41244633:C>*	*BRCA1* Frameshift	p.Gly972AspfsX28	1	The p.Gly972AspfsX28 variant in BRCA1 has not been previously reported in individuals with hereditary breast and/or ovarian cancer (HBOC) and was absent from large population studies, but has been reported in ClinVar (Variation ID: 54716). This variant is predicted to cause a frameshift, which alters the protein’s amino acid sequence beginning at position 972 and leads to a premature termination codon 28 amino acids downstream. This alteration is then predicted to lead to a truncated or absent protein. Loss of function of the BRCA1 gene is an established disease mechanism in autosomal dominant HBOC. In summary, although additional studies are required to fully establish its clinical significance, this variant meets criteria to be classified as likely pathogenic for autosomal dominant HBOC. ACMG/AMP Criteria applied: PVS1, PM2.
17:41244866:TT>*	*BRCA1* Frameshift	p.Lys894ThrfsX8	1	The p.Lys894ThrfsX8 variant in BRCA1 has been reported in >80 individuals with BRCA1-associated cancers (Friedman 1994, Walsh 2011, Wong-Brown 2016, Zhang 2011, Liede 2000, Breast Cancer Information Core (BIC) database), segregated with disease in >10 affected relatives, and was absent from large population studies. This variant is predicted to cause a frameshift, which alters the protein’s amino acid sequence beginning at position 894 and leads to a premature termination codon 8 amino acids downstream. This alteration is then predicted to lead to a truncated or absent protein. Heterozygous loss of function of the BRCA1 gene is an established disease mechanism in individuals with hereditary breast and ovarian cancer (HBOC). In addition, this variant was classified as Pathogenic on April 22, 2016 by the ClinGen-approved ENIGMA expert panel (ClinVar SCV000282287.1). In summary, the p.Lys894fs variant meets our criteria to be classified as pathogenic for HBOC in an autosomal dominant manner based upon the predicted impact to the protein. ACMG/AMP Criteria applied: PVS1, PS4, PP1_Strong, PM2.
17:41244922:C>A	*BRCA1* Nonsense	p.Gly876X	1	The p.Gly876X variant in BRCA1 has not been previously reported in individuals with hereditary breast and/or ovarian cancer (HBOC) and was absent from large population studies. This nonsense variant leads to a premature termination codon at position 876, which is predicted to lead to a truncated or absent protein. Loss of function of the BRCA1 gene is an established disease mechanism in autosomal dominant HBOC. In summary, although additional studies are required to fully establish its clinical significance, this variant meets criteria to be classified as likely pathogenic for autosomal dominant HBOC. ACMG/AMP Criteria applied: PVS1, PM2.
17:41245073:G>*	*BRCA1* Frameshift	p.Asp825GlufsX21	4	The p.Asp825GlufsX21 variant in BRCA1 has been reported in >25 individuals with BRCA1-related cancers and has been reported as a founder variant in Scandinavian populations (Pennington 2013, Cunningham 2014, Maxwell 2017, Hakansson 1997, BIC database). It has also been identified in 1/113380 European chromosomes by gnomAD (http://gnomad.broadinstitute.org); however, this frequency is low enough to be consistent with the frequency of hereditary breast and ovarian cancer (HBOC) in the general population. This variant is predicted to cause a frameshift, which alters the

				protein’s amino acid sequence beginning at position 825 and leads to a premature termination codon 21 amino acids downstream. This alteration is then predicted to lead to a truncated or absent protein. Loss of function of the BRCA1 gene is an established disease mechanism in autosomal dominant hereditary breast and ovarian cancer syndrome. Additionally, this variant was classified as Pathogenic on Apr 22, 2016 by the ClinGen-approved ENIGMA expert panel (Variation ID: 37472). In summary, this variant meets criteria to be classified as pathogenic for autosomal dominant HBOC. ACMG/AMP Criteria applied: PVS1, PM2, PS4.
17:41245285:C>*	*BRCA1* Frameshift	p.Glu755LysfsX10	1	The p.Glu755LysfsX10 variant in BRCA1 has not been previously reported in individuals with hereditary breast and/or ovarian cancer (HBOC) and was absent from large population studies, but has been reported in ClinVar (Variation ID: 54517). This variant is predicted to cause a frameshift, which alters the protein’s amino acid sequence beginning at position 755 and leads to a premature termination codon 10 amino acids downstream. This alteration is then predicted to lead to a truncated or absent protein. Loss of function of the BRCA1 gene is an established disease mechanism in autosomal dominant HBOC. In summary, although additional studies are required to fully establish its clinical significance, this variant meets criteria to be classified as likely pathogenic for autosomal dominant HBOC. ACMG/AMP Criteria applied: PVS1, PM2.
17:41245354:C>A	*BRCA1* Nonsense	p.Glu732X	1	The p.Glu732X variant in BRCA1 has been reported in at least 6 individuals with a personal or family history of breast and/or ovarian cancer (Kadouri 2004, BIC database). It was absent from large population studies. This variant leads to a premature termination codon at position 732, which is predicted to lead to a truncated or absent protein. Loss of function of the BRCA1 gene is an established disease mechanism in autosomal dominant hereditary breast and ovarian cancer syndrome (HBOC). Additionally, this variant was classified as Pathogenic on Sep 8, 2016 by the ClinGen-approved ENIGMA expert panel (Variation ID: 54492). In summary, this variant meets criteria to be classified as pathogenic for autosomal dominant HBOC. ACMG/AMP Criteria applied: PVS1, PM2, PS4_Moderate.
17:41245670:*>ACTA	*BRCA1* Frameshift	p.Val627SerfsX4	1	The p.Val627SerfsX4 variant in BRCA1 has been reported in at least 17 individuals with hereditary breast and/or ovarian cancer (HBOC; Kwong 2014, Balz 2002, Thomassen 2008, BIC database) and was absent from large population studies. This variant is predicted to cause a frameshift, which alters the protein’s amino acid sequence beginning at position 627 and leads to a premature termination codon 4 amino acids downstream. This alteration is then predicted to lead to a truncated or absent protein. Loss of function of the BRCA1 gene is an established disease mechanism in autosomal dominant HBOC. Additionally, this variant was classified as Pathogenic on Sep 8, 2016 by the ClinGen-approved ENIGMA expert panel (Variation ID: 54376). In summary, this variant meets criteria to be classified as pathogenic for autosomal dominant HBOC. ACMG/AMP Criteria applied: PVS1, PM2, PS4.
17:41245861:G>A	*BRCA1* Nonsense	p.Gln563X	1	The p.Gln563X variant in BRCA1 (also referred to as 1806C>T) has been reported in >100 individuals with BRCA1-associated cancers (Shattuck-Eidens 1995, Wagner 1998, Pohlreich 2003, Foretova 2004, Salazar 2006, Krajc 2008, Janav 2010, Zuradelli 2010, Blay 2013, Cunningham 2014, Kluska 2015, Cini 2016, Breast Cancer Information Core (BIC) database). It was also identified in 5/66568 European chromosomes by the Exome Aggregation Consortium (ExAC, http://exac.broadinstitute.org; dbSNP rs80356898); however, this frequency is low enough to be consistent with the frequency of hereditary breast and ovarian cancer (HBOC) in the general population. This nonsense variant leads to a premature termination codon at position 563, which is predicted to lead to a truncated or absent protein. Heterozygous loss of function of the BRCA1 gene is an established disease mechanism in HBOC. Furthermore, this variant was classified as Pathogenic on April 22, 2016 by the ClinGen-approved ENIGMA expert panel (ClinVar SCV000282262.1). In summary, this variant meets criteria to be classified as pathogenic for HBOC in an autosomal dominant manner. PVS1, PM2, PS4.
17:41246077:G>A	*BRCA1* Nonsense	p.Gln491X	1	The p.Gln491X variant in BRCA1 has been reported in at least 6 individuals with a personal or family history of breast and/or ovarian cancer (HBOC; Rashid 2006, Park 2017, Sun 2017, BIC database). It was absent from large population studies. This variant leads to a premature termination codon at

				position 491, which is predicted to lead to a truncated or absent protein. Loss of function of the BRCA1 gene is an established disease mechanism in autosomal dominant hereditary breast and ovarian cancer syndrome (HBOC). Additionally, this variant was classified as Pathogenic on Apr 22, 2016 by the ClinGen-approved ENIGMA expert panel (Variation ID: 54264). In summary, this variant meets criteria to be classified as pathogenic for autosomal dominant HBOC. ACMG/AMP Criteria applied: PVS1, PM2, PS4_Moderate.
17:41246192:T>*	*BRCA1* Frameshift	p.Glu453ArgfsX22	1	The p.Glu453ArgfsX22 variant in BRCA1 has not been previously reported in individuals with hereditary breast and/or ovarian cancer (HBOC) and was absent from large population studies, but has been reported in ClinVar (Variation ID: 125495). This variant is predicted to cause a frameshift, which alters the protein's amino acid sequence beginning at position 453 and leads to a premature termination codon 22 amino acids downstream. This alteration is then predicted to lead to a truncated or absent protein. Loss of function of the BRCA1 gene is an established disease mechanism in autosomal dominant HBOC. In summary, although additional studies are required to fully establish its clinical significance, this variant meets criteria to be classified as likely pathogenic for autosomal dominant HBOC. ACMG/AMP Criteria applied: PVS1, PM2.
17:41246846:TG>*	*BRCA1* Frameshift	p.Thr234LysfsX3	1	The p.Thr234LysfsX3 variant in BRCA1 has not been previously reported in individuals with hereditary breast and/or ovarian cancer (HBOC) and was absent from large population studies. This variant is predicted to cause a frameshift, which alters the protein's amino acid sequence beginning at position 234 and leads to a premature termination codon 3 amino acids downstream. This alteration is then predicted to lead to a truncated or absent protein. Loss of function of the BRCA1 gene is an established disease mechanism in autosomal dominant HBOC. In summary, although additional studies are required to fully establish its clinical significance, this variant meets criteria to be classified as likely pathogenic for autosomal dominant HBOC. ACMG/AMP Criteria applied: PVS1, PM2.
17:41247869:T>A	*BRCA1* Nonsense	p.Lys222X	1	The p.Lys222X variant in BRCA1 has not been previously reported in individuals with hereditary breast and/or ovarian cancer (HBOC) and was absent from large population studies. This nonsense variant leads to a premature termination codon at position 222, which is predicted to lead to a truncated or absent protein. Loss of function of the BRCA1 gene is an established disease mechanism in autosomal dominant HBOC. In summary, although additional studies are required to fully establish its clinical significance, this variant meets criteria to be classified as likely pathogenic for autosomal dominant HBOC. ACMG/AMP Criteria applied: PVS1, PM2.
17:41256153:C>A	*BRCA1* Nonsense	p.Glu143X	4	The p.Glu143X variant in BRCA1 has been reported in >15 individuals with BRCA1-related cancers (Shattuck-Eidens 1997, Caligo 2009, Cunningham 2014, Susswein 2016, Lowery 2018, Yurgelun 2019, BIC database). It was absent from large population studies. This frameshift variant leads to a premature termination codon at position 143, which is predicted to lead to a truncated or absent protein. Loss of function of the BRCA1 gene is an established disease mechanism in autosomal dominant hereditary breast and ovarian cancer syndrome (HBOC). Additionally, this variant was classified as Pathogenic on Apr 22, 2016 by the ClinGen-approved ENIGMA expert panel (Variation ID: 37581). In summary, this variant meets criteria to be classified as pathogenic for autosomal dominant HBOC. ACMG/AMP Criteria applied: PVS1, PM2, PS4.
17:41256280:T>*	*BRCA1* splice site	c.302-2delA	1	The c.302-2delA variant in BRCA1 has been reported in at least 16 individuals with breast and/or ovarian cancer (Shattuck-Eidens 1997, Gayther 1999, Southey 2003, Chen 2006, Borg 2010, BIC database [https://research.nhgri.nih.gov/bic/]) and segregated with disease in at least 9 affected relatives from 1 family (Southey 2003). This variant has also been reported by other clinical laboratories in ClinVar (Variation ID# 54753) and was absent from large population databases. The c.302-2delA variant occurs in the invariant region (+/- 1,2) of the splice consensus sequence and is predicted to cause altered splicing leading to an abnormal or absent protein. In vitro studies as well as sequencing of cDNA from individuals with this variant showed that it leads to activation of a cryptic splice site, leading to 10 bp frameshift at the beginning of the next exon and resulting in the addition of 14 new amino acid residues and a premature stop codon (Chen 2006). In summary, this variant meets criteria to be classified as pathogenic for hereditary breast and ovarian cancer (HBOC) in an

				autosomal dominant manner based upon presence in multiple affected individuals, segregation studies, absence from the general population, functional evidence, and predicted impact on the protein. ACMG/AMP Criteria applied (Richards 2015): PVS1, PS4, PP1_Strong.
17:41256884:C>A	*BRCA1* splice site	c.301+1G>T	2	The c.301+1G>T variant in BRCA1 has not been previously reported in individuals with hereditary breast and/or ovarian cancer (HBOC) but has been identified in 1/113556 European chromosomes by gnomAD (http://gnomad.broadinstitute.org); however, this frequency is low enough to be consistent with the frequency of hereditary breast and ovarian cancer (HBOC) in the general population. This variant has also been reported in ClinVar (Variation ID: 246510). This variant occurs within the canonical splice site (+/- 1,2) and is predicted to cause altered splicing leading to an abnormal or absent protein. In vitro functional studies support an impact on protein function (Findlay 2018). Loss of function of the BRCA1 gene is an established disease mechanism in autosomal dominant HBOC. In summary, although additional studies are required to fully establish its clinical significance, this variant meets criteria to be classified as likely pathogenic for autosomal dominant HBOC ACMG/AMP Criteria applied: PVS1, PM2
17:41256884:C>G	*BRCA1* splice site	c.301+1G>C	3	The c.301+1G>C variant in BRCA1 has not been previously reported in individuals with hereditary breast and/or ovarian cancer (HBOC) but has been identified in 1/113556 European chromosomes by gnomAD (http://gnomad.broadinstitute.org); however, this frequency is low enough to be consistent with the frequency of hereditary breast and ovarian cancer (HBOC) in the general population. This variant has also been reported in ClinVar (Variation ID: 267517). This variant occurs within the canonical splice site (+/- 1,2) and is predicted to cause altered splicing leading to an abnormal or absent protein. In vitro functional studies support an impact on protein function (Findlay 2018). Loss of function of the BRCA1 gene is an established disease mechanism in autosomal dominant HBOC. In summary, although additional studies are required to fully establish its clinical significance, this variant meets criteria to be classified as likely pathogenic for autosomal dominant HBOC ACMG/AMP Criteria applied: PVS1, PM2.
Lynch syndrome variants				
2:47635539:G>A	*MSH2* splice site	c.212-1G>A	2	The c.212-1G>A variant in MSH2 has been reported in 1 individual affected with colorectal cancer (Overbeek 2007), 1 individual with Lynch syndrome (De Lellis 2013), 1 individual with bladder cancer (van der Post 2010), and 1 individual with clinical suspicion of Lynch syndrome (Ramsoekh 2008). It was absent from large population studies. This variant was classified as Pathogenic on September 5, 2013 by the ClinGen-approved InSiGHT expert panel (Variation ID 90892). This variant occurs within the canonical splice site (+/- 1,2) and is predicted to cause altered splicing leading to an abnormal or absent protein. Loss of function of the MSH2 gene is an established disease mechanism in autosomal dominant Lynch syndrome. In summary, the c.212-1G>A variant meets criteria to be classified as pathogenic for Lynch syndrome. ACMG/AMP criteria applied: PVS1, PM2, PS4_Supporting.
2:47643458:CT>*	*MSH2* Frameshift	p.Gln324ValfsX8	2	The p.Gln324ValfsX8 (c.969_970delTC) variant in MSH2 has been reported in 4 individuals with colorectal cancer/Lynch syndrome and segregated with disease in 4 affected family members (Sjursen 2010, Hansen 2014, Tanyi 2014, Koder 2017). It was also absent from large population studies. This variant is predicted to cause a frameshift, which alters the protein's amino acid sequence beginning at position 324 and leads to a premature termination codon 8 amino acids downstream. This alteration is then predicted to lead to a truncated or absent protein. Another variant, c.970_971delCA (ClinVar ID 91262), resulting in the same amino acid change has been identified in several individuals with Lynch syndrome (Goldberg 2015). Loss of function of the MSH2 gene is an established disease mechanism in autosomal dominant Lynch syndrome. In summary, the p.Gln324ValfsX8 variant meets criteria to be classified as pathogenic for Lynch syndrome. ACMG/AMP criteria applied: PVS1, PS1, PS4_Supporting, PM2, PP1.
2:47657016:*>TACCG	*MSH2* Frameshift	p.Leu407ThrfsX7	1	The p.Leu407ThrfsX7 variant in MSH2 has not been previously reported in individuals with Lynch syndrome and was absent from large population studies. This variant is predicted to cause a frameshift, which alters the protein's amino acid sequence beginning at position 407 and leads to a

				premature termination codon 7 amino acids downstream. This alteration is then predicted to lead to a truncated or absent protein. Loss of function of the MSH2 gene is an established disease mechanism in autosomal dominant Lynch syndrome. In summary, although additional studies are required to fully establish its clinical significance, the p.Leu407ThrfsX7 variant meets criteria to be classified as likely pathogenic for Lynch syndrome. ACMG/AMP criteria applied: PVS1, PM2.
2:47705528:T>*	*MSH2* Frameshift	p.Cys778AlafsX34	1	The p.Cys778AlafsX34 variant in MSH2 has not been previously reported in individuals with Lynch syndrome and was absent from large population studies. This variant is predicted to cause a frameshift, which alters the protein's amino acid sequence beginning at position 778 and leads to a premature termination codon 34 amino acids downstream. This alteration is then predicted to lead to a truncated or absent protein. Loss of function of the MSH2 gene is an established disease mechanism in autosomal dominant Lynch syndrome. In summary, although additional studies are required to fully establish its clinical significance, the p.Cys778AlafsX34 variant meets criteria to be classified as likely pathogenic for Lynch syndrome. ACMG/AMP criteria applied: PVS1, PM2.
2:48010634:T>A	*MSH6* splice site	c.260+2T>A	1	The c.260+2T>A variant in MSH6 has not been previously reported in individuals with Lynch syndrome or in large population studies but has been reported by other clinical laboratories in ClinVar (Variation ID 492704). This variant occurs within the canonical splice site (+/- 1,2) and is predicted to cause altered splicing leading to an abnormal or absent protein. Heterozygous loss-of-function of the MSH6 gene is an established disease mechanism in individuals with Lynch syndrome. In summary, this variant meets criteria to be classified as likely pathogenic for autosomal dominant Lynch syndrome based upon predicted impact to the protein and absence in controls. ACMG/AMP criteria applied: PVS1, PM2.
2:48025764:C>G	*MSH6* Nonsense	p.Tyr214X,NA	3	The p.Tyr214X variant in MSH6 has been previously reported in the heterozygous state in at least 1 individual with colorectal cancer (Verma 1999), 1 individual with metastatic prostate cancer (Pritchard 2016), and 1 individual with multiple sebaceous adenomas/embryonal teratoma of the testis and family history of gastrointestinal cancers (Murphy 2008), and in the compound heterozygous state in 1 individual with constitutional mismatch repair-deficiency (Scott 2007). It was absent from large population studies. This variant was classified as Pathogenic on September 5, 2013 by the ClinGen-approved InSiGHT expert panel (Variation ID 89547). This nonsense variant leads to a premature termination codon at position 214, which is predicted to lead to a truncated or absent protein. Heterozygous loss of function of the MSH6 gene is an established disease mechanism in individuals with Lynch syndrome. In summary, this variant meets criteria to be classified as pathogenic for autosomal dominant Lynch syndrome. ACMG/AMP criteria applied: PVS1, PM2, PS4_Supporting, PM3.
2:48025796:*>TG	*MSH6* Frameshift	p.Glu226ValfsX21	1	The p.Glu226ValfsX21 variant in MSH6 has been reported in 1 individual with Lynch syndrome (as c.674insTG; Talseth-Palmer 2010) and was absent from large population studies. This variant is predicted to cause a frameshift, which alters the protein's amino acid sequence beginning at position 226 and leads to a premature termination codon 21 amino acids downstream. This alteration is then predicted to lead to a truncated or absent protein. Loss of function of the MSH6 gene is an established disease mechanism in autosomal dominant Lynch syndrome. In summary, the p.Glu226ValfsX21 variant meets criteria to be classified as pathogenic for Lynch syndrome. ACMG/AMP criteria applied: PVS1, PM2.
2:48025840:C>T	*MSH6* Nonsense	p.Arg240X	1	The p.Arg240X variant in MSH6 has been previously reported in 1 individual referred for multigene panel testing (Espenschied 2017), 2 individuals with colorectal cancer, one of which was MSI-low (DeRycke 2017, Yan 2008), and 1 individual with Lynch syndrome and segregated with disease in 2 affected family members (Kovac 2011). It was also identified in 1/30606 of South Asian chromosomes by gnomAD (http://gnomad.broadinstitute.org). This variant was classified as Pathogenic on September 5, 2013 by the ClinGen-approved InSiGHT expert panel (Variation ID 89559). This nonsense variant leads to a premature termination codon at position 240, which is predicted to lead to a truncated or absent protein. Heterozygous loss of function of the MSH6 gene is an established disease mechanism in individuals with Lynch syndrome. In summary, this variant meets criteria to be

				classified as pathogenic for autosomal dominant Lynch syndrome. ACMG/AMP criteria applied: PVS1, PM2, PS4_Supporting.
2:48025863:*>C	*MSH6* Frameshift	p.Arg248ProfsX8	1	The p.Arg248ProfsX8 variant in MSH6 has not been previously reported in individuals with Lynch syndrome and was absent from large population studies. This variant is predicted to cause a frameshift, which alters the protein's amino acid sequence beginning at position 248 and leads to a premature termination codon 8 amino acids downstream. This alteration is then predicted to lead to a truncated or absent protein. Heterozygous loss of function of the MSH6 gene is an established disease mechanism in individuals with autosomal dominant Lynch syndrome. In summary, although additional studies are required to fully establish its clinical significance, the p.Arg248ProfsX8 variant meets criteria to be classified as likely pathogenic for autosomal dominant Lynch syndrome. ACMG/AMP Criteria applied: PVS1; PM2.
2:48025864:C>T	*MSH6* Nonsense	p.Arg248X	1	The p.Arg248X variant in MSH6 has been previously reported in 1 individual with Lynch syndrome (Steinke 2008) and 2 individuals with colorectal cancer (Wijnen 1999, Hendricks 2003). It was absent from large population studies. This variant was classified as Pathogenic on September 5, 2013 by the ClinGen-approved InSiGHT expert panel (Variation ID 89563). This nonsense variant leads to a premature termination codon at position 248, which is predicted to lead to a truncated or absent protein. Heterozygous loss of function of the MSH6 gene is an established disease mechanism in individuals with Lynch syndrome. In summary, this variant meets criteria to be classified as pathogenic for autosomal dominant Lynch syndrome. ACMG/AMP criteria applied: PVS1, PM2, PS4_Supporting.
2:48026014:C>T	*MSH6* Nonsense	p.Arg298X	1	The p.Arg298X variant in MSH6 has been reported in at least 2 individuals with MSH6-associated cancers (Goodfellow 2015, Susswein et al. 2016) and has also been identified in 1/15278 African chromosomes by the genome Aggregation Database (gnomAD, http://gnomad.broadinstitute.org). This nonsense variant leads to a premature termination codon at position 298, which is predicted to lead to a truncated or absent protein. Heterozygous loss of function of the MSH6 gene is an established disease mechanism in Lynch syndrome. Moreover, this variant was classified as pathogenic on September 5, 2013 by the ClinGen-approved InSiGHT expert panel (ClinVar SCV000108264.2). In summary, this variant meets criteria to be classified as pathogenic for Lynch syndrome in an autosomal dominant manner based upon the predicted impact on the protein and low frequency in controls. ACMG/AMP Criteria applied: PS4_Supporting, PM2, PVS1.
2:48026310:*>T	*MSH6* Frameshift	p.Tyr397LeufsX4	1	The p.Tyr397LeufsX4 variant in MSH6 has not been previously reported in individuals with Lynch syndrome and was absent from large population studies. It has been reported in ClinVar (variation ID 418928). This variant is predicted to cause a frameshift, which alters the protein's amino acid sequence beginning at position 379 and leads to a premature termination codon 4 amino acids downstream. This alteration is then predicted to lead to a truncated or absent protein. Heterozygous loss of function of the MSH6 gene is an established disease mechanism in individuals with autosomal dominant Lynch syndrome. In summary, although additional studies are required to fully establish its clinical significance, thep.Tyr397LeufsX4 variant meets criteria to be classified as likely pathogenic for autosomal dominant Lynch syndrome. ACMG/AMP Criteria applied: PVS1; PM2.
2:48026468:T>C	*MSH6* Missense	p.Leu449Pro	1	The p.Leu449Pro variant in MSH6 has been reported in >10 individuals with MSH6-associated cancers from a large multigenerational Swedish family (Cederquist 2004, Cederquist 2005). In addition, the majority of tumors sampled from these individuals showed microsatellite instability and lacked MSH6 expression. This variant has also been identified in 3/113542 of European chromosomes by gnomAD (http://gnomad.broadinstitute.org). Computational prediction tools and conservation analysis suggest that this variant may impact the protein, though this information is not predictive enough to determine pathogenicity. Furthermore, this variant was classified as Pathogenic on Sept. 5, 2013 by the ClinGen-approved InSiGHT Expert Panel (ClinVar SCV000107853). In summary, the p.Leu449Pro variant meets criteria to be classified as pathogenic for Lynch Syndrome in an autosomal dominant manner based upon segregation studies and low frequency in controls. ACMG/AMP Criteria applied: PP1_Strong, PM2, PP3, PS3_Strong.

2:48026473:T>*	*MSH6* Frameshift	p.Phe451SerfsX2	2	The p.Phe451SerfsX2 variant in MSH6 has been reported in at least 1 individual with colorectal cancer (Shirts 2016) and was absent from large population studies. It has also been reported in ClinVar (Variation ID 224534) and classified as Pathogenic by several clinical labs. This variant is predicted to cause a frameshift, which alters the protein's amino acid sequence beginning at position 451 and leads to a premature termination codon 2 amino acids downstream. This alteration is then predicted to lead to a truncated or absent protein. Loss of function of the MSH6 gene is an established disease mechanism in autosomal dominant Lynch syndrome. In summary, although additional studies are required to fully establish its clinical significance, the p.Phe451SerfsX2 variant meets criteria to be classified as likely pathogenic for Lynch syndrome. ACMG/AMP criteria applied: PVS1, PM2
2:48026752:G>*	*MSH6* Frameshift	p.Glu544LysfsX27	1	The p.Glu544LysfsX27 variant in MSH6 has not been previously reported in individuals with Lynch Syndrome and was absent from large population studies. This variant is predicted to cause a frameshift, which alters the protein's amino acid sequence beginning at position 544 and leads to a premature termination codon 27 amino acids downstream. This alteration is then predicted to lead to a truncated or absent protein. Heterozygous loss of function of the MSH6 gene is an established disease mechanism in individuals with Lynch syndrome. In summary, this variant meets criteria to be classified as likely pathogenic for autosomal dominant Lynch syndrome. ACMG/AMP criteria applied: PVS1, PM2.
2:48026753:AA>*	*MSH6* Frameshift	p.Lys545ArgfsX17	1	The p.Lys545ArgfsX17 variant in MSH6 has been reported in the compound heterozygous state in 1 individual with constitutional mismatch repair deficiency (Peters 2009) and in the heterozygous state in 1 individual with colorectal cancer (Susswein 2016). Another variant affecting the same position (p.Lys545ArgfsX25) has been reported in 2 individuals with breast/endometrial cancer (Susswein 2016). It was absent from large population studies. It has also been reported in ClinVar (Variation ID 140961) and was classified as Pathogenic by several clinical labs. This variant is predicted to cause a frameshift, which alters the protein's amino acid sequence beginning at position 545 and leads to a premature termination codon 17 amino acids downstream. This alteration is then predicted to lead to a truncated or absent protein. Loss of function of the MSH6 gene is an established disease mechanism in autosomal dominant Lynch syndrome. In summary, the p.Lys545ArgfsX17 variant meets criteria to be classified as Pathogenic for Lynch syndrome. ACMG/AMP criteria applied: PVS1, PM2, PM3.
2:48026928:AAAG>*	*MSH6* Frameshift	p.Glu604LeufsX5	1	The p.Glu604LeufsX5 variant in MSH6 has been reported in 1 individual with colon cancer (Ohmiya 2001) and 1 individual with Lynch syndrome (Chika 2015), and segregated with disease in 1 affected relative (Chika 2015). It has also been reported in the compound heterozygous state in an individual with early onset colorectal cancer, vitiligo and systemic lupus erythematosus (Rahner 2008). It was absent from large population studies. This variant was classified as Pathogenic on September 5, 2013 by the ClinGen-approved InSiGHT expert panel (Variation ID 89224). This variant is predicted to cause a frameshift, which alters the protein's amino acid sequence beginning at position 604 and leads to a premature termination codon 5 amino acids downstream. This alteration is then predicted to lead to a truncated or absent protein. Loss of function of the MSH6 gene is an established disease mechanism in autosomal dominant Lynch syndrome. In summary, the p.Glu604LeufsX5 variant meets criteria to be classified as pathogenic for Lynch syndrome. ACMG/AMP criteria applied: PVS1, PM2, PS4_supporting.
2:48027179:G>A	*MSH6* Missense	p.Gly686Asp	1	The p.Gly686Asp variant in MSH6 has been reported in at least 5 individuals with Lynch syndrome (Yurgelun 2015, Goodfellow 2015, Thompson 2013, Hampel 2008, DeRycke 2017) and in 1 individual with breast cancer, who also carried a likely pathogenic variant in CHEK2 (Susswein 2016). It was absent from large population studies. This variant was classified as likely pathogenic on September 5, 2013 by the ClinGen-approved InSiGHT expert panel (Variation ID 89245) and several clinical labs. Computational prediction tools and conservation analyses suggest that this variant may impact the protein, and in vitro and in vivo (patient tumor) functional studies provide further evidence that this variant impacts protein function (Houlleberghs 2017, Goodfellow 2015, Thompson 2013). In summary,

				although additional studies are required to fully establish its clinical significance, this variant meets criteria to be classified as likely pathogenic for autosomal dominant Lynch syndrome. ACMG/AMP Criteria applied: PM2, PS4_Moderate, PS3_Moderate, PP3.
2:48027195:*>A	*MSH6* Frameshift	p.Cys694MetfsX4	2	The p.Cys694MetfsX4 variant in MSH6 has been reported in 2 individuals with colorectal cancer (DeRycke 2017, Hansen 2017) and in 1 individual with a family history of Lynch-associated tumors (Shirts 2016). It was absent from large population studies. This variant has been reported in ClinVar (Variation ID 187516) as pathogenic by several clinical labs. This variant is predicted to cause a frameshift, which alters the protein's amino acid sequence beginning at position 694 and leads to a premature termination codon 4 amino acids downstream. This alteration is then predicted to lead to a truncated or absent protein. Loss of function of the MSH6 gene is an established disease mechanism in autosomal dominant Lynch syndrome. In summary, the p.Cys694MetfsX4 variant meets criteria to be classified as pathogenic for Lynch syndrome. ACMG/AMP criteria applied: PVS1, PM2, PS4_supporting.
2:48027269:CAGT>*	*MSH6* Frameshift	p.Val717AlafsX18	2	The p.Val717AlafsX18 variant in MSH6 has been reported in 1 individual with endometrial cancer, 2 individuals with ovarian cancer, 3 individuals with colorectal cancer and 1 individual with Lynch syndrome (Walsh 2011, Baglietto 2010, Nilbert 2009, Pal 2012, Pagin 2013, Kolodner 1999, Hirasawa 2017). It has also been identified in 1/30612 of South Asian and in 1/34584 of Latino chromosomes by gnomAD (http://gnomad.broadinstitute.org). This variant was classified as Pathogenic on September 5, 2013 by the ClinGen-approved InSiGHT expert panel (Variation ID 89256). This variant is predicted to cause a frameshift, which alters the protein's amino acid sequence beginning at position 717 and leads to a premature termination codon 18 amino acids downstream. This alteration is then predicted to lead to a truncated or absent protein. Loss of function of the MSH6 gene is an established disease mechanism in autosomal dominant Lynch syndrome. In summary, the p.Val717AlafsX18 variant meets criteria to be classified as pathogenic for Lynch syndrome. ACMG/AMP criteria applied: PVS1, PM2, PS4_Moderate.
2:48027316:C>T	*MSH6* Nonsense	p.Arg732X	2	The p.Arg732X variant in MSH6 has been reported in at least 5 individuals with hereditary non-polyposis colorectal cancer (HNPCC) and related tumors (Plaschke 2004, Steinke 2008, Baglietto 2010, Giraldez 2010, Song 2014) and was absent from large population studies. This nonsense variant leads to a premature termination codon at position 732, which is predicted to lead to a truncated or absent protein. Heterozygous loss-of-function of the MSH6 gene is an established disease mechanism for Lynch syndrome. In summary, this variant meets criteria to be classified as pathogenic for Lynch syndrome in an autosomal dominant manner. ACMG/AMP Criteria applied: PM2, PVS1, PS4_Moderate.
2:48027656:ATGAA>*	*MSH6* Nonsense	p.Tyr845X	1	The p.Tyr845X variant in MSH6 has not been previously reported in individuals with Lynch Syndrome and was absent from large population studies. This nonsense variant leads to a premature termination codon at position 845, which is predicted to lead to a truncated or absent protein. Heterozygous loss of function of the MSH6 gene is an established disease mechanism in individuals with Lynch syndrome. In summary, this variant meets criteria to be classified as likely pathogenic for autosomal dominant Lynch syndrome. ACMG/AMP criteria applied: PVS1, PM2.
2:48027853:C>T	*MSH6* Nonsense	p.Arg911X	2	The p.Arg911X variant in MSH6 has been reported in at least 13 individual with MSH6-associated cancers and segregated in at least 4 affected relatives (Goodfellow 2003, Buttin 2004, Hendriks 2004, Plaschke 2004, Hampel 2006, Talseth-Palmer 2010, Pal 2012, Palles 2013, Rosty 2014, Susswein 2015, Akbari 2017, Raskin 2017). This variant has also been reported in ClinVar (Variation ID 89312) and has been identified in 1/15426 European chromosomes by gnomAD (http://gnomad.broadinstitute.org). This nonsense variant leads to a premature termination codon at position 911, which is predicted to lead to a truncated or absent protein. Heterozygous loss of function of the MSH6 gene is an established disease mechanism in individuals with Lynch syndrome. In summary, this variant meets criteria to be classified as pathogenic for Lynch syndrome in an autosomal dominant manner. ACMG/AMP Criteria applied: PVS1; PS4; PP1; PM2.

2:48027878:A>*	*MSH6* Frameshift	p.Lys920ArgfsX25	1	The p.Lys920ArgfsX25 variant in MSH6 has not been previously reported in individuals with Lynch Syndrome and was absent from large population studies. This variant has also been reported in ClinVar (Variation ID 428433). This variant is predicted to cause a frameshift, which alters the protein's amino acid sequence beginning at position 920 and leads to a premature termination codon 27 amino acids downstream. This alteration is then predicted to lead to a truncated or absent protein. Heterozygous loss of function of the MSH6 gene is an established disease mechanism in individuals with Lynch syndrome. In summary, this variant meets criteria to be classified as likely pathogenic for autosomal dominant Lynch syndrome. ACMG/AMP criteria applied: PVS1, PM2.
2:48028096:G>T	*MSH6* Nonsense	p.Glu992X	1	The p.Glu992X variant in MSH6 has not been previously reported in individuals with Lynch Syndrome and was absent from large population studies. This nonsense variant leads to a premature termination codon at position 992, which is predicted to lead to a truncated or absent protein. Heterozygous loss of function of the MSH6 gene is an established disease mechanism in individuals with Lynch syndrome. In summary, this variant meets criteria to be classified as likely pathogenic for autosomal dominant Lynch syndrome. ACMG/AMP criteria applied: PVS1, PM2.
2:48028135:C>T	*MSH6* Nonsense	p.Arg1005X	1	The p.Arg1005X variant in MSH6 has been previously reported in 5 individuals with colorectal cancer, 1 individual with endometrial cancer, 1 individual with multiple colorectal adenomas, and 2 individuals with Lynch syndrome, and segregated with disease in 2 affected relatives from 1 family (Colley 2005, Castillejo 2011, Plaschke 2004, De Lellis2013, Goodfellows 2015, Adachi 2017, Pan 2019, Keranen 2018, Tanskanen 2013). It has also been identified in 1/99286 of European chromosomes by gnomAD (http://gnomad.broadinstitute.org). This variant was classified as Pathogenic on September 5, 2013 by the ClinGen-approved InSiGHT expert panel (Variation ID 89330). This nonsense variant leads to a premature termination codon at position 1005, which is predicted to lead to a truncated or absent protein. Heterozygous loss of function of the MSH6 gene is an established disease mechanism in individuals with Lynch syndrome. In summary, this variant meets criteria to be classified as pathogenic for autosomal dominant Lynch syndrome. ACMG/AMP criteria applied: PVS1, PM2, PS4_Moderate.
2:48028225:C>T	*MSH6* Nonsense	p.Arg1035X	1	The p.Arg1035X variant in MSH6 has been previously reported in 1 individual with constitutive mismatch repair deficiency syndrome (compound heterozygous), 1 individual with pediatric CNS tumor, 1 individual with ovarian cancer and 6 individuals with endometrial cancer (1 of whom also carried a nonsense variant in BRIP1), and segregated with disease in 2 affected relatives from 2 families (Pal 2102, Norquist 2016, Planck 1999, Hendriks 2004, Devlin 2008, Planschke 2004, Ling 2018, Grobner 2018). It has also been identified in 1/18338 of East Asian and 2/112342 of European chromosomes by gnomAD (http://gnomad.broadinstitute.org). This variant was classified as Pathogenic on September 5, 2013 by the ClinGen-approved InSiGHT expert panel (Variation ID 89338). This nonsense variant leads to a premature termination codon at position 1035, which is predicted to lead to a truncated or absent protein. Heterozygous loss of function of the MSH6 gene is an established disease mechanism in individuals with Lynch syndrome. In summary, this variant meets criteria to be classified as pathogenic for autosomal dominant Lynch syndrome. ACMG/AMP criteria applied: PVS1, PM2, PS4_Moderate, PM3.
2:48028229:TG>*	*MSH6* Frameshift	p.Phe1037LeufsX2	1	The p.Phe1037LeufsX2 variant in MSH6 has been reported in 2 individuals with colorectal cancer and 1 individual with pancreatic cancer (Sjursen 2016, DeRycke 217, Slavin 2018). It was absent from large population studies. This variant has been reported in ClinVar (Variation ID 525755), classified as pathogenic by 1 clinical lab. This variant is predicted to cause a frameshift, which alters the protein's amino acid sequence beginning at position 1037 and leads to a premature termination codon 2 amino acids downstream. This alteration is then predicted to lead to a truncated or absent protein. Loss of function of the MSH6 gene is an established disease mechanism in autosomal dominant Lynch syndrome. In summary, the p.Phe1037LeufsX2 variant meets criteria to be classified as pathogenic for Lynch syndrome. ACMG/AMP criteria applied: PVS1, PM2, PS4_supporting.
2:48030612:C>T	*MSH6* Missense	p.Arg1076Cys	6	The p.Arg1076Cys variant in MSH6 has been reported in the heterozygous state in at least 12 individuals with Lynch syndrome-associated cancers (Plaschke 2004, Limburg 2011, Liccardo 2017,

				Rohlin 2017, Rubio 2016, Schultheis 2016, Klarskov 2011, Schofield 2009, Nilbert 2009) and in the homozygous state in 1 individual with relatively late onset colorectal cancer, suggesting that it may be a hypomorphic allele (Gardes 2012). Additionally, the p.Arg1076Cys variant has also been reported in the compound heterozygous state in 5 individuals (from four families) with clinical features of constitutional mismatch repair deficiency (Okkels 2006, Plaschke 2006, Jasperson 2011, Gardes 2012). It has also been identified in 0.03% (5/18386) of East Asian chromosomes by gnomAD (http://gnomad.broadinstitute.org). Immunohistochemistry staining of tumor tissue samples in the majority of affected individuals showed loss of MSH6 staining, providing evidence that the Arg1076Cys variant may impact protein function. Computational prediction tools and conservation analyses suggest that this variant may impact the protein. This variant was classified as Likely Pathogenic on March 9, 2018 by the ClinGen-approved InSiGHT expert panel (RCV000074823.4). In summary, although additional studies are required to fully establish its clinical significance, this variant meets criteria to be classified as likely pathogenic for autosomal dominant Lynch syndrome. ACMG/AMP Criteria applied: PM3, PS4_Moderate, PS3_Supporting, PP3, PM2_Supporting.
2:48030623:TC>*	*MSH6* Frameshift	p.Leu1080ValfsX12	2	The p.Leu1080ValfsX12 variant in MSH6 has been reported in 1 individual with endometrial cancer (Batte 2014) and was absent from large population studies. This variant has been reported in ClinVar (Variation ID 218060), classified as pathogenic by several clinical labs. This variant is predicted to cause a frameshift, which alters the protein's amino acid sequence beginning at position 1080 and leads to a premature termination codon 12 amino acids downstream. This alteration is then predicted to lead to a truncated or absent protein. Loss of function of the MSH6 gene is an established disease mechanism in autosomal dominant Lynch syndrome. In summary, the p.Leu1080ValfsX12 variant meets criteria to be classified as likely pathogenic for Lynch syndrome. ACMG/AMP criteria applied: PVS1, PM2.
2:48030647:CT>*	*MSH6* Frameshift	p.Phe1088LeufsX4	1	The p.Phe1088LeufsX4 variant in MSH6 has not been previously reported in individuals with Lynch Syndrome or in large population studies. This variant is predicted to cause a frameshift, which alters the protein's amino acid sequence beginning at position 1088 and leads to a premature termination codon 4 amino acids downstream. This alteration is then predicted to lead to a truncated or absent protein. Heterozygous loss of function of the MSH6 gene is an established disease mechanism in individuals with Lynch syndrome. In summary, this variant meets criteria to be classified as likely pathogenic for autosomal dominant Lynch syndrome based upon the predicted impact to the protein and absence from the general population. ACMG/AMP criteria applied: PVS1, PM2.
2:48030815:*>AA	*MSH6* Frameshift	p.Met1144LysfsX2	1	The p.Met1144LysfsX2 variant in MSH6 has not been previously reported in individuals with Lynch Syndrome and was absent from large population studies. This variant is predicted to cause a frameshift, which alters the protein's amino acid sequence beginning at position 1144 and leads to a premature termination codon 2 amino acids downstream. This alteration is then predicted to lead to a truncated or absent protein. Heterozygous loss of function of the MSH6 gene is an established disease mechanism in individuals with Lynch syndrome. In summary, this variant meets criteria to be classified as likely pathogenic for autosomal dominant Lynch syndrome. ACMG/AMP criteria applied: PVS1, PM2.
2:48032123:*>A	*MSH6* Frameshift	p.Arg1172LysfsX5	1	The p.Arg1172LysfsX5 variant in MSH6 has been previously reported in at least 12 individuals with Lynch syndrome or associated cancers, and segregated with disease in 3 affected relatives from 1 family (Overbeek 2007, Plaschke 2004, Sjursen 2010, Wijnen 1999, Haraldsdottir 2017, Nilbert 2009, van der Post 2010, Jenkins 2006, Steinke 2008, Song 2014, Woods 2010, Nilbert 2009) and in 1 individual with constitutive mismatch repair deficiency in the compound heterozygous state (Soplepmann 2016). It was identified in 1/35414 of Latino and in 2/129076 of European chromosomes by gnomAD (http://gnomad.broadinstitute.org). This variant was classified as Pathogenic on September 5, 2013 by the ClinGen-approved InSiGHT expert panel (Variation ID 89404). This variant is predicted to cause a frameshift, which alters the protein's amino acid sequence beginning at position 1172 and leads to a premature termination codon 5 amino acids downstream. This alteration is then predicted to lead to a truncated or absent protein. Heterozygous loss of function

				of the MSH6 gene is an established disease mechanism in individuals with Lynch syndrome. In summary, this variant meets criteria to be classified as pathogenic for autosomal dominant Lynch syndrome. ACMG/AMP criteria applied: PVS1, PS4_Moderate, PM2, PM3, PP1.
2:48033780:C>T	*MSH6* Nonsense	p.Arg1331X	1	The p.Arg1331X variant in MSH6 has been reported in the heterozygous state in 6 individuals with MSH6-associated cancers (Stormorken 2005, Sjursen 2010, Bonadona 2011, Susswein 2015, LMM data). This variant has also been reported in 2 individuals with clinical features of constitutional mismatch repair syndrome in the compound heterozygous state with another MSH6 variant (Plaschke 2006) or in the homozygous state (Lavoine 2015). In addition, The p.Arg1331X variant has been identified in 1/30646 South Asian chromosomes by gnomAD (http://gnomad.broadinstitute.org). This nonsense variant leads to a premature termination codon at position 1331. This alteration occurs within the terminal 50 bases of the second to last exon and is more likely to escape nonsense mediated decay (NMD) and result in a truncated protein. Studies have demonstrated that the variant results in skipping of exon 9, giving rise to a truncated mRNA leading to drastically reduced MSH6 protein levels, suggesting that this truncated protein is unstable (Plaschke 2006). Moreover, the p.Arg1331X variant has been classified as pathogenic on September 5, 2013 by the ClinGen-approved InSiGHT Expert Panel (ClinVar SCV000108181.2). In summary, this variant meets criteria to be classified as pathogenic for Lynch syndrome in an autosomal dominant manner. ACMG/AMP Criteria applied: PS4_Moderate, PM2, PVS1_Strong, PM3_Supporting.
3:37035120:C>*	*MLH1* Frameshift	p.Pro28GlnfsX8	1	The p.Pro28GlnfsX8 variant in MLH1 has not been previously been reported in individuals with MLH1-related cancers and was absent from large population studies. This variant is predicted to cause a frameshift, which alters the protein's amino acid sequence beginning at position 28 and leads to a premature termination codon 8 amino acids downstream. This alteration is then predicted to lead to a truncated or absent protein. Loss of function of the MLH1 gene is an established disease mechanism in autosomal dominant Lynch syndrome. In summary, this variant meets criteria to be classified as likely pathogenic for autosomal dominant Lynch syndrome. ACMG/AMP criteria applied: PVS1, PM2.
3:37038192:G>A	*MLH1* Missense	p.Gly67Arg	2	The p.Gly67Arg variant in MLH1 has been identified in a large number of individuals with Lynch syndrome and segregated with disease in at least 4 affected family members in 2 families (Tannergard 1995, Mitchell 2002, Alazzouzi 2005, Lagerstedt Robinson 2007, InSiGHT database: http://chromium.lovd.nl/LOVD2/colon_cancer/variants.php). This variant was absent from large population studies. Mice carrying the p.Gly67Arg variant have a strong cancer predisposition phenotype (Avdievich 2008). Additionally, this variant has been classified as pathogenic on Sep 5, 2013 by the ClinGen-approved InSiGHT panel (ClinVar SCV000106471.2). In summary, this variant meets criteria to be classified as pathogenic for Lynch syndrome in an autosomal dominant manner. ACMG/AMP criteria applied: PM2, PS4, PP1_Supporting, PS3.
3:37042536:C>T	*MLH1* Nonsense	p.Arg100X	3	The p.Arg100X variant in MLH1 has been reported in at least 10 individuals with Lynch syndrome and segregated with disease in at least 6 affected individuals from 2 families (Wang 1999, Samowitz 2001, Renknen 2003, Renkonen 2004, Taylor 2003 Mangold 2005, Choi 2009, Hampel 2005, Lagerstedt 2007, Peel 2000, Gylling 2007). It was absent from large population studies. This variant was classified as Pathogenic on September 5, 2013 by the ClinGen-approved InSiGHT expert panel (Variation ID 36550). This nonsense variant leads to a premature termination codon at position 100, which is predicted to lead to a truncated or absent protein. Loss of function of the MLH1 gene is an established disease mechanism in autosomal dominant Lynch syndrome. In summary, this variant meets criteria to be classified as pathogenic for autosomal dominant Lynch syndrome. ACMG/AMP Criteria applied: PVS1, PM2, PS4_Moderate, PP1_Moderate.
3:37053348:A>T	*MLH1* Nonsense	p.Lys195X	1	The p.Lys195X variant in MLH1 has been reported in 1 individual with colorectal cancer (Hinrichsen 2015). It was absent from large population studies and has been reported in ClinVar (Variation ID 218025). This nonsense variant leads to a premature termination codon at position 195, which is predicted to lead to a truncated or absent protein. Loss of function of the MLH1 gene is an established disease mechanism in autosomal dominant Lynch syndrome. In summary, although additional

				studies are required to fully establish its clinical significance, this variant meets criteria to be classified as pathogenic for autosomal dominant Lynch syndrome. ACMG/AMP Criteria applied: PVS1, PM2
3:37067499:G>A	*MLH1* splice site	c.1409+1G>A	1	The c.1409+1G>A variant in MLH1 has been previously reported in 1 individual suspected to have Lynch syndrome (Irmejs 2007) and was absent from large population studies. This variant was classified as Pathogenic by several clinical labs in ClinVar and as Likely pathogenic on September 5, 2013 by the ClinGen-approved InSiGHT expert panel (Variation ID 89718). This variant occurs within the canonical splice site (+/- 1,2) and is predicted to cause altered splicing leading to an abnormal or absent protein. Other variants involving this position (c.1409+1G>C, c.1409+1G>T) have been classified as likely pathogenic/Pathogenic in ClinVar. Heterozygous loss-of-function of the MLH1 gene is an established disease mechanism in individuals with Lynch syndrome. In summary, this variant meets criteria to be classified as pathogenic for autosomal dominant Lynch syndrome. ACMG/AMP criteria applied: PVS1, PM2, PM5.
3:37070316:A>*	*MLH1* Frameshift	p.Asp484ValfsX7	1	The p.Asp484ValfsX7 variant in MLH1 has been previously reported in at least 1 individual with Lynch syndrome (Sjursen 2016) and was absent from large population studies. It is predicted to cause a frameshift, which alters the protein's amino acid sequence beginning at position 484 and leads to a premature termination codon 7 amino acids downstream. This alteration is then predicted to lead to a truncated or absent protein. Heterozygous loss of function of the MLH1 gene is an established disease mechanism in individuals with Lynch syndrome. In summary, this variant meets criteria to be classified as pathogenic for autosomal dominant Lynch syndrome. ACMG/AMP Criteria applied: PVS1, PM2.
3:37083758:G>A	*MLH1* splice site	c.1668-1G>A	2	The c.1668-1G>A variant in MLH1 has been reported in at least 4 individuals with Lynch Syndrome (Lamberti 2006, Arnold 2009, Pérez-Carbonell 2012, Shirts 2016) and was found to segregate with disease in 2 additional affected individuals in one family (Arnold 2009). This variant was absent from large population studies. This variant occurs in the invariant region (+/- 1,2) of the splice consensus sequence and has been observed to cause altered splicing leading to an abnormal or absent protein (Arnold 2009, Pérez-Carbonell 2012). Heterozygous loss of function of the MLH1 gene is an established disease mechanism in Lynch Syndrome. Additionally, this variant has been classified as Likely pathogenic on Sep 5, 2013 by the ClinGen-approved InSiGHT panel (ClinVar SCV000106296.2). In summary, this variant meets criteria to be classified as pathogenic for Lynch Syndrome in an autosomal dominant manner. ACMG/AMP criteria applied: PM2, PS4_Supporting, PVS1.
3:37083822:G>A	*MLH1* splice site	p.Ser577Ser	6	The p.Ser577Ser variant in MLH1 has been previously reported in at least 17 individuals with Lynch syndrome (Auclair 2006, Tournier 2008, Bozzao 2011, Kurzawski 2006, Kohonen-Corish 1996, Mangold 2005, Hinrichsen 2014, Betz 2010, Simbolo 2015, Pagenstecher 2006, De Lellis 2013) and was absent from large population studies. This variant was classified as Pathogenic by several clinical labs in ClinVar and on September 5, 2013 by the ClinGen-approved InSiGHT expert panel (Variation ID 89857). This variant is located in the last three bases of the exon, which is part of the 5' splice region. Computational tools do not predict a splicing impact, though this information is not predictive enough to rule out pathogenicity. In vitro and in vivo functional studies support an impact on protein function, due to out-of-frame exon skipping (Auclair 2006, Tournier 2008, Kohonen-Corish 1996, Betz 2010, Pagenstecher 2006, De Lellis 2013). Heterozygous loss-of-function of the MLH1 gene is an established disease mechanism in individuals with Lynch syndrome. In summary, this variant meets criteria to be classified as pathogenic for autosomal dominant Lynch syndrome. ACMG/AMP criteria applied: PS3, PS4, PM2.
3:37089123:GAA>*	*MLH1* Deletion	p.Lys618del	1	The p.Lys618del (also known as p.Lys616del) variant in MLH1 has been reported at least 7 individuals affected with Lynch syndrome (including at least 1 family with Turcot syndrome) and segregated with disease in at least 16 affected family members from 4 families (Syngal 1999, Raevaara 2003, Takahashi 2007, Ricker 2017, Viel 1997, Wijnen 1996, Hamilton 1995). It has also been identified in 1/113698 of European chromosomes by gnomAD (http://gnomad.broadinstitute.org). This variant was classified as Pathogenic by several clinical labs in ClinVar and on September 5,

				2013 by the ClinGen-approved InSiGHT expert panel (Variation ID 17080). This variant is a deletion of 1 amino acid at position 618 and is not predicted to alter the protein reading-frame. In vitro and in vivo functional studies support an impact on protein function (Raevaara 2003, Schmutte 2001, Guerrette 1999, Kondo 2003, Takahashi 2007). In summary, this variant meets criteria to be classified as pathogenic for autosomal dominant Lynch syndrome. ACMG/AMP Criteria applied: PP1_Strong, PM2, PS3_Moderate, PS4_Moderate, PM4_Supporting.
3:37090464:C>T	*MLH1* Missense	p.Arg687Trp	1	The p.Arg687Trp variant in MLH1 has been previously reported in at least 16 individuals with Lynch syndrome (von Salome 2018, Jakubowska 2001, Furukawa 2002, Caldes 2002) and 3 homozygous siblings affected with constitutive mismatch repair deficiency (Durno 2012). It is considered a founder mutation for Lynch syndrome in Sweden (von Salome 2018). It has also been identified in 3/30612 of South Asian chromosomes by gnomAD (http://gnomad.broadinstitute.org). This variant was classified as Pathogenic by several clinical labs in ClinVar and on September 5, 2013 by the ClinGen-approved InSiGHT expert panel (Variation ID 90014). Computational prediction tools and conservation analyses suggest that this variant may impact the protein, though this information is not predictive enough to determine pathogenicity. In vitro functional studies provide some evidence that this variant mildly impacts protein function (Takahashi 2007, Christensen 2009, Kansikas 2011); however, these types of assays may not accurately represent biological function. In summary, this variant meets criteria to be classified as pathogenic for autosomal dominant Lynch syndrome. ACMG/AMP criteria applied: PS4, PP1_Strong, PM3, PM2_Supporting, PP4.
7:6029554:T>*	*PMS2* Frameshift	p.Arg341GlyfsX15	2	The p.Arg341GlyfsX15 variant in PMS2 has been identified in at least 1 individual with PMS2-associated cancer and segregated with the disease in 2 affected family members (one with endometrial cancer and one with colon polyps; Worthley 2005). This variant was absent from large population studies. This variant is predicted to cause a frameshift, which alters the protein's amino acid sequence beginning at position 341 and leads to a premature termination codon 15 amino acids downstream. This alteration is then predicted to lead to a truncated or absent protein. Heterozygous loss of function of function of the PMS2 gene is an established disease mechanism in Lynch syndrome. In summary, this variant meets criteria to be classified as pathogenic for Lynch syndrome in an autosomal dominant manner based upon predicted impact to the protein. ACMG/AMP Criteria applied: PM2, PVS1.
7:6035204:CTGT>*	*PMS2* Frameshift	p.Arg287SerfsX19	1	The p.Arg287SerfsX19 variant in PMS2 has been reported in at least 5 individuals with PMS2-associated cancers in the heterozygous state and segregated with disease in at least 1 affected relative (Rohlin 2017, Suerink 2016, Yurgelun 2015,Moline 2013, Senter 2008, Hendriks 2006). This variant has been identified in 1/24960 African and in 1/129158 European chromosomes by gnomAD (http://gnomad.broadinstitute.org) and was classified as pathogenic on September 5, 2013 by the ClinGen-approved inSIGHT expert panel (ClinVar ID 91375). This variant is predicted to cause a frameshift, which alters the protein's amino acid sequence beginning at position 287 and leads to a premature termination codon 19 amino acids downstream. This alteration is then predicted to lead to a truncated or absent protein. Loss of function of the PMS2 gene is an established disease mechanism in autosomal dominant Lynch syndrome. In summary, this variant meets criteria to be classified as pathogenic for autosomal dominant Lynch syndrome. ACMG/AMP Criteria applied: PVS1, PM2, PS4_Moderate.
7:6035245:G>A	*PMS2* Nonsense	p.Gln275X	2	The p.Gln275X variant in PMS2 has been reported in at least 4 heterozygous individuals with PMS2-associated cancers and in 1 compound heterozygous individual with constitutive mismatch repair deficiency (Espenschied 2017, Susswein 2016, Suerink 2016, Hansen 2014, Vaughn 2013, Yeung 2013). This variant has been identified in 2/34590 of Latino chromosomes by gnomAD (http://gnomad.broadinstitute.org) and has been reported in ClinVar (pathogenic by several clinical labs (ClinVar ID 127796). This nonsense variant leads to a premature termination codon at position 275, which is predicted to lead to a truncated or absent protein. Loss of function of the PMS2 gene is an established disease mechanism in autosomal dominant Lynch syndrome. In summary, this variant

				meets criteria to be classified as pathogenic for autosomal dominant Lynch syndrome. ACMG/AMP Criteria applied: PVS1, PM2, PS4_Supporting, PM3.
7:6038747:G>A	*PMS2* Nonsense	p.Gln233X	2	The p.Gln233X variant in PMS2 has been previously reported in >15 individuals with PMS2-associated cancers (Rossi 2017, van der Klift 2016, Susswein 2016, Suerink 2016, ten Broeke 2015, Niessen 2009) and has been detected in 3/129188 of European chromosomes by gnomAD (https://gnomad.broadinstitute.org). This variant was classified as Pathogenic on September 5, 2013 by the ClinGen-approved InSiGHT expert panel (Variation ID 91362). This nonsense variant leads to a premature termination codon at position 233, which is predicted to lead to a truncated or absent protein. Loss of function of the PMS2 gene is an established disease mechanism in autosomal dominant Lynch syndrome. In summary, this variant meets criteria to be classified as pathogenic for autosomal dominant Lynch syndrome. ACMG/AMP Criteria applied: PVS1, PM2, PS4.
7:6038884:*>C	*PMS2* Frameshift	p.Val187GlyfsX62	1	The p.Val187GlyfsX62 variant in PMS2 has not been previously reported in individuals with Lynch syndrome and was absent from large population studies. This variant is predicted to cause a frameshift, which alters the protein's amino acid sequence beginning at position 187 and leads to a premature termination codon 62 amino acids downstream. This alteration is then predicted to lead to a truncated or absent protein. Loss of function of the PMS2 gene is an established disease mechanism in autosomal dominant Lynch syndrome. In summary, although additional studies are required to fully establish its clinical significance, this variant meets criteria to be classified as likely pathogenic for autosomal dominant Lynch syndrome. ACMG/AMP Criteria applied: PVS1, PM2.

Supplementary Table 6: Baseline characteristics of the 48,812 UK Biobank participants

Characteristic	Total Subjects (N= 48,812)
Age at enrollment, mean (SD), y	57.1 (8.0)
Female sex, n (%)	26,597 (54.5)
Race, n (%)	
White	45,536 (93.3)
Black	999 (2)
Asian	1219 (2.5)
Other	1058 (2.2)
Prevalent disease at enrollment	
Coronary artery disease, n (%)	2074 (4.2)
Breast cancer, n (%) *	1348 (2.8)
Colorectal cancer, n (%)	315 (0.65)
Incident disease during follow-up	
Coronary artery disease, n (%)	1573 (3.4)
Breast cancer, n (%) *	564 (1.2)
Colorectal cancer, n (%)	361 (0.74)
Monogenic risk variant carriers	
FH variant, n (%)	130 (0.27)
HBOC variant, n (%) *	233 (0.48)
LS variant, n (%)	76 (0.15)

In the 26,597 female participants, there were 1337 (5.03%) prevalent breast cancer cases, 557 (2.2%) incident breast cancer cases, and 115 (0.43%) HBOC mutation carriers. Abbreviations: FH familial hypercholesterolemia, HBOC hereditary breast and ovarian cancer syndrome, LS Lynch syndrome.

Supplementary Table 7: Baseline characteristics of the 48,812 UK Biobank participants by coronary artery disease status

	With coronary artery disease (n=2,074)	Without coronary artery disease (n=46,738)
Age, mean (SD), y	62.48 (5.8)	56.83 (8.0)
Female sex, n (%)	554 (26.7)	26,043 (55.7)
Race, n (%)		
White	1926 (92.9)	43,610 (93.3)
Black	27 (1.3)	972 (2.1)
Asian	88 (4.2)	1131 (2.4)
Other	33 (1.6)	1020 (2.2)
Hypertension, n (%)	1546 (74.5)	14,573 (31.2)
Diabetes, n (%)	483 (23.3)	2969 (6.4)
Chronic kidney disease, n (%)	116 (5.6)	418 (0.9)
Current or former smoking, n (%)	1229 (59.6)	20,366 (43.7)
Body mass index, mean (SD), kg m^{-2}	29.30 (5.03)	27.33 (4.78)
Family history of heart disease, n (%)	677 (41.7)	8889 (23.8)

Shown above are characteristics of prevalent coronary artery disease cases at baseline vs. the remainder of the cohort.

Supplementary Table 8: Likelihood-ratio test for linearity assessment of polygenic score-disease relationships

Disease	Model 1	Model 2	p-value
Coronary artery disease	$CAD \sim PS_{CAD}$	$CAD \sim PS_{CAD} + (PS_{CAD})_2 + (PS_{CAD})_3$	0.98
Breast cancer	$BrCA \sim PS_{BrCA}$	$BrCA \sim PS_{BrCA} + (PS_{BrCA})_2 + (PS_{BrCA})_3$	0.07
Colorectal cancer	$CRC \sim PS_{CRC}$	$CRC \sim PS_{CRC} + (PS_{CRC})_2 + (S_{CRC})_3$	0.69

PS is the regression residual of regressing disease-specific polygenic score on the first four principal components of ancestry.
Abbreviations: CAD coronary artery disease, BrCA breast cancer, CRC colorectal cancer

Supplementary Table 9: Baseline characteristics of the 26,597 female UK Biobank participants by breast cancer status

	With breast cancer (n=1,337)	Without breast cancer (n=25,260)
Age, mean (SD), y	59.63 (6.8)	56.46 (8.0)
Female sex, n (%)	1,337 (100)	25,260 (100)
Race, n (%)		
White	1276 (95.4)	23,442 (92.8)
Black	12 (0.9)	612 (2.4)
Asian	25 (1.9)	597 (2.3)
Other	24 (1.8)	607 (2.4)
Current or former smoking, n (%)	573 (43.0)	9942 (39.5)
Body mass index, mean (SD), kg m^{-2}	26.70 (4.7)	27.08 (5.3)
Family history of breast cancer, n (%)	287 (21.5)	2975 (11.8)

Shown above are prevalent breast cancer cases at baseline vs. the remainder of the cohort.

Supplementary Table 10: Baseline characteristics of the 48,812 UK Biobank participants by colorectal cancer status

	With colorectal cancer (n=315)	Without colorectal cancer (n=48,497)
Age, mean (SD), y	61.69 (6.4)	57.04 (8.0)
Female sex, n (%)	132 (41.9)	26,465 (54.6)
Race, n (%)		
White	303 (96.2)	45,233 (93.3)
Black	6 (1.9)	993 (2.0)
Asian	3 (0.9)	1216 (2.5)
Other	3 (0.9)	1050 (2.2)
Current or former smoking, n (%)	170 (54.3)	21,425 (44.3)
Body mass index, mean (SD), kg m^{-2}	27.95 (4.7)	27.41 (4.81)
Family history of bowel cancer, n (%)	73 (23.2)	5814 (12.0)

Shown above are prevalent colorectal cancer cases at baseline vs. the remainder of the cohort.

Supplementary Table 11: Phenotype definitions of coronary artery disease, breast cancer, and colorectal cancer in the cohort study from the UK Biobank

**Coronary artery disease**		
**Data-field ID**	**Data-field description**	**Data-codings**
**Self-report**		
20002	Self-reported medical condition (non-cancer)	1075
20004	Self-reported previous operations or procedures	107,010,951,523
**Death records**		
40001	Primary cause of death (ICD10) from the death registry	I21,I210,I211,I212,I213,I214,I219,I22,I220,I221,I228,I229,I23,I230,I231,I232,I233,I234,I235,I236,I238,I24,I240,I241,I248,I249,I251,I252,I255,I256,I258,I259
40002	Secondary cause of death (ICD10) from the death registry	I21,I210,I211,I212,I213,I214,I219,I22,I220,I221,I228,I229,I23,I230,I231,I232,I233,I234,I235,I236,I238,I24,I240,I241,I248,I249,I251,I252,I255,I256,I258,I259
**Hospitalization diagnosis records**		
41202	Primary diagnoses (ICD10) from hospitalization records	I21,I210,I211,I212,I213,I214,I219,I22,I220,I221,I228,I229,I23,I230,I231,I232,I233,I234,I235,I236,I238,I24,I240,I241,I248,I249,I251,I252,I255,I256,I258,I259,I25,I250
41203	Primary diagnoses (ICD9) from hospitalization records	410,411,412,4119,4129,4140,4148,4149,4109
41204	Secondary diagnoses (ICD10) from hospitalization records	I21,I210,I211,I212,I213,I214,I219,I22,I220,I221,I228,I229,I23,I230,I231,I232,I233,I234,I235,I236,I238,I24,I240,I241,I248,I249,I251,I252,I255,I256,I258,I259,I25,I250
41205	Secondary diagnoses (ICD9) from hospitalization records	410,411,412,4119,4129,4140,4148,4149,4109
**Hospitalization procedural records**		

41200	Primary operation or procedure codes from hospitalization records	K40,K401,K402,K403,K404,K408,K409,K41,K411,K412,K413,K414,K418,K419,K42,K421,K422,K423,K424,K428,K429,K43,K431,K432,K433,K434,K438,K439,K44,K441,K442,K448,K449,K451,K452,K453,K454,K455,K456,K458,K459,K46,K461,K462,K463,K464,K465,K468,K469,K471,K491,K492,K493,K494,K498,K499,K501,K502,K504,K751,K752,K753,K754,K758,K759
41210	Secondary operation or procedure codes from hospitalization records	K40,K401,K402,K403,K404,K408,K409,K41,K411,K412,K413,K414,K418,K419,K42,K421,K422,K423,K424,K428,K429,K43,K431,K432,K433,K434,K438,K439,K44,K441,K442,K448,K449,K451,K452,K453,K454,K455,K456,K458,K459,K46,K461,K462,K463,K464,K465,K468,K469,K471,K491,K492,K493,K494,K498,K499,K501,K502,K504,K751,K752,K753,K754,K758,K759
**Breast cancer**		
**Data-field ID**	**Data-field description**	**Data-codings**
**Self-report**		
20001	Self-reported medical condition (cancer)	1002
**Death records**		
40001	Primary cause of death (ICD10) from the death registry	C50,C500,C501,C502,C503,C504,C505,C506,C508
40002	Secondary cause of death (ICD10) from the death registry	C50,C500,C501,C502,C503,C504,C505,C506,C508
**Cancer registry**		
40006	Type of cancer (ICD10) from the cancer registry	C50,C500,C501,C502,C503,C504,C506,C508,C509,D050,D051,D057,D059
40013	Type of cancer (ICD10) from the cancer registry	174,175,1740,1741,1742,1743,1744,1745,1746,1748,1749,2330,2383
**Hospitalization diagnosis records**		
41202	Primary diagnoses (ICD10) from hospitalization records	C50,C500,C501,C502,C503,C504,C505,C506,C508,C509,D05,D050,D051,D057,D059,D486,Z853
41203	Primary diagnoses (ICD9) from hospitalization records	174,174,1740,1743,1744,1745,1748,1749,2330,2383,2393
41204	Secondary diagnoses (ICD10) from hospitalization records	C50,C500,C501,C502,C503,C504,C505,C506,C508,D05,D050,D051,D057,D059,D486,Z853

41205	Secondary diagnoses (ICD9) from hospitalization records	174,1749,2330,V103
**Colorectal cancer**		
**Data field ID**	**Data field description**	**Coding ID's**
**Self-report**		
20001	Self-reported medical condition (cancer)	10,221,023
**Death records**		
40001	Primary cause of death (ICD10) from the death registry	C180,C182,C183,C184,C185,C186,C187,C188,C189
40002	Secondary cause of death (ICD10) from the death registry	C180,C182,C183,C184,C185,C186,C187,C188,C189
**Cancer registry**		
40006	Type of cancer (ICD10) from the cancer registry	C18,C180,C181,C182,C183,C184,C185,C186,C187,C188,C189,C19,C20,D010,D011,D012,D374,D375
40013	Type of cancer (ICD10) from the cancer registry	153,154,1530,1531,1532,1533,1534,1535,1536,1537,1538,1539,1540,1541,1548,2303,2304
**Hospitalization diagnosis records**		
41202	Primary diagnoses (ICD10) from hospitalization records	C18,C180,C182,C183,C184,C185,C186,C187,C188,C189,C19,C20,D010,D011,D012,D374,D375
41203	Primary diagnoses (ICD9) from hospitalization records	153,154,1530,1532,1533,1534,1536,1537,1539,1540,1541
41204	Secondary diagnoses (ICD10) from hospitalization records	C18,C180,C182,C183,C184,C185,C186,C187,C188,C189,C19,C20,D010,D011,D012,D374,D375
41205	Secondary diagnoses (ICD9) from hospitalization records	153,153,154,1532,1533,1541

Disease definitions were assessed based on hospital inpatient data dating back to 1977, cancer registry data dating back to 1957, and death registry data available from time of initial enrollment from 2006 onward. Data field IDs in the table are linked to the specific UK Biobank web pages.
