# Supplementary material for: Polygenic background modifies penetrance of monogenic variants for tier 1 genomic conditions
Source: Nat Commun. 2020 Aug 20;11:3635. doi: 10.1038/s41467-020-17374-3 (PMC7441381; doi:10.1038/s41467-020-17374-3)
Supplement: Supplementary file 2 — Reporting Summary [file 41467_2020_17374_MOESM2_ESM.pdf]

nature research

Corresponding author(s): Amit V. Khera

Last updated by author(s): 06/16/2020

Reporting Summary

Nature Research wishes to improve the reproducibility of the work that we publish. This form provides structure for consistency and transparency in reporting. For further information on Nature Research policies, see Authors & Referees and the Editorial Policy Checklist.

Statistics

For all statistical analyses, confirm that the following items are present in the figure legend, table legend, main text, or Methods section.

n/a	Confirmed	
☐	☒	The exact sample size (n) for each experimental group/condition, given as a discrete number and unit of measurement
☐	☒	A statement on whether measurements were taken from distinct samples or whether the same sample was measured repeatedly
☐	☒	The statistical test(s) used AND whether they are one- or two-sided<br>*Only common tests should be described solely by name; describe more complex techniques in the Methods section.*
☐	☒	A description of all covariates tested
☐	☒	A description of any assumptions or corrections, such as tests of normality and adjustment for multiple comparisons
☐	☒	A full description of the statistical parameters including central tendency (e.g. means) or other basic estimates (e.g. regression coefficient) AND variation (e.g. standard deviation) or associated estimates of uncertainty (e.g. confidence intervals)
☐	☒	For null hypothesis testing, the test statistic (e.g. F, t, r) with confidence intervals, effect sizes, degrees of freedom and P value noted<br>*Give P values as exact values whenever suitable.*
☒	☐	For Bayesian analysis, information on the choice of priors and Markov chain Monte Carlo settings
☒	☐	For hierarchical and complex designs, identification of the appropriate level for tests and full reporting of outcomes
☒	☐	Estimates of effect sizes (e.g. Cohen's d, Pearson's r), indicating how they were calculated

Our web collection on statistics for biologists contains articles on many of the points above.

Software and code

Policy information about availability of computer code

Data collection	No custom software or algorithms were developed or used for data collection.
Data analysis	Sequencing reads were aligned to the reference genome using the Burrows-Wheeler Aligner algorithm. Variants were called using the Genome Analysis Toolkit (GATK) version 3.4 and SAMtools version 1.8. The pathogenicity of observed genetic variants for each of three diseases was assesssed according to current American College of Medical Genetics and Genomics (ACMG)/Association of Molecular Pathology (AMP) criteria. Previously published polygenic scores for each of three diseases were calculated using PLINK version 2.0 using the 'SCORE' function. Statistical analyses were conducted using R software, version 3.5. The R package 'waffect' was used for power calculations. The R package 'survival' was used to estimate the probability of disease by age 75.

For manuscripts utilizing custom algorithms or software that are central to the research but not yet described in published literature, software must be made available to editors/reviewers. We strongly encourage code deposition in a community repository (e.g. GitHub). See the Nature Research guidelines for submitting code & software for further information.

Data

Policy information about availability of data

All manuscripts must include a data availability statement. This statement should provide the following information, where applicable:

- Accession codes, unique identifiers, or web links for publicly available datasets
- A list of figures that have associated raw data
- A description of any restrictions on data availability

Phenotypes derived as part of this manuscript – including calculated polygenic scores, monogenic variant carrier status, and disease status endpoints -- will be returned to the UK Biobank for dissemination to approved investigators. Further information on obtaining approval for access to the UK Biobank data is available at: https://www.ukbiobank.ac.uk/researchers. We also included Supplementary Table 11 with the codings of all phenotypes so that they can be replicated by other investigators from raw phenotypes in the UK Biobank. Criteria used to support pathogenicity assessment for monogenic risk variants are provided in Supplementary Tables 1 and 5. The raw weights for calculating the coronary artery disease polygenic score are available for download from the Broad Institute Cardiovascular

Disease Knowledge Portal http://www.broadcvdi.org. The raw weights for calculating the breast cancer and colorectal cancer polygenic scores are available from the original publications.21,22 Aggregate summaries of the Color Genomic data are available at https://data.color.com. The Genome Aggregation Database (gnoMAD) is publicly available at http://gnomad.broadinstitute.org.

Field-specific reporting

Please select the one below that is the best fit for your research. If you are not sure, read the appropriate sections before making your selection.

☒ Life sciences ☐ Behavioural & social sciences ☐ Ecological, evolutionary & environmental sciences

For a reference copy of the document with all sections, see nature.com/documents/nr-reporting-summary-flat.pdf

Life sciences study design

All studies must disclose on these points even when the disclosure is negative.

Sample size

We included all eligible participants in two case-control studies — one for coronary artery disease derived from the UK Biobank and one for breast cancer derived from the Color Genomics commercial laboratory — and additional participants of the UK Biobank with whole exome sequencing and genotyping array data available. The observed frequency of rare monogenic variants for CDC Tier 1 genomic conditions in those studies were consistent with prior reports (PMID 32347951, 28008009, 30646163, 24549055).

Data exclusions

Within the UK Biobank studies, individuals were excluded based on excessive DNA contamination, low target base coverage, putative sex chromosome aneupoloidy, outliers for heterozygosity, or low genotyping array call rate. For each pair of related individuals (second-degree or closer), one was removed. For the Color Genomics study, individuals were removed for low coverage of target bases or they failed the clinical genetics CLIA pipeline quality control criteria. These exclusions were prespecified prior to the analysis.

Samples that failed standard exome sequencing quality control, and one individual of a related pair (up to second degree) were excluded. Those exclusions are well-established in genomic studies to avoid bias. Additionally, participants who withdrew consent following initial enrollment in the UK Biobank were excluded.

Replication

We extended the findings in the two case-control studies to an independent cohort of 48,812 unrelated participants.

Randomization

No randomization was performed as part of this study. Group allocations were based on disease status, monogenic variant carrier status, and polygenic risk status. The covariates that were used are age, sex, and the first four principal components of genetic ancestry.

Blinding

Variants meeting clinical criteria of pathogenicity (pathogenic or likely pathogenic) based on American College of Medical Genetics and Genomics (ACMG)/Association of Molecular Pathology (AMP) criteria were identified by clinical geneticists blinded to the case/control status of the participant.

Reporting for specific materials, systems and methods

We require information from authors about some types of materials, experimental systems and methods used in many studies. Here, indicate whether each material, system or method listed is relevant to your study. If you are not sure if a list item applies to your research, read the appropriate section before selecting a response.

Materials & experimental systems

n/a	Involved in the study	
☒	☐	Antibodies
☒	☐	Eukaryotic cell lines
☒	☐	Palaeontology
☒	☐	Animals and other organisms
☐	☒	Human research participants
☒	☐	Clinical data

Methods

n/a	Involved in the study	
☒	☐	ChIP-seq
☒	☐	Flow cytometry
☒	☐	MRI-based neuroimaging

Human research participants

Policy information about studies involving human research participants

Population characteristics

In the coronary artery disease case-control study (N=12,852), cases and controls had similar age and sex. As expected, cases had higher rates of cardiac risk factors (hypertension, smoking, chronic kidney disease, and family history of heart disease). In the breast cancer case-control study (N=19,264), cases were older than controls, both groups had similar rates of family history, and there was enrichment for genetic variants in both cases and controls, which is expected given that participants were referred for genetic testing. The cohort study (N=48,812) consisted of a subset of the UK Biobank with available exome sequencing data. As reported previously and highlighted in the limitations of this manuscript, UK Biobank participants tended to have lower disease rates than the general population.

Recruitment

UK Biobank is a prospective national biobank study that enrolled middle-aged adult participants between 2006 and 2010. Case definitions were based on self-report, hospitalization records, cancer registry, and death registry records.
The breast cancer case-control study was derived from Color Genomics commercial testing laboratory and case definition was based on self-report at enrollment.

Ethics oversight

Informed consent was obtained from all participants. Analysis of UK Biobank data was performed under application number 7089 and approved by the Partners Healthcare institutional review board. The Color Genomics study was approved by the Western Institutional Review Board.

Note that full information on the approval of the study protocol must also be provided in the manuscript.
